# A Total Synthesis of (−)-Strychnine Using Photoredox Catalysis

**DOI:** 10.1021/jacsau.5c01709

**Published:** 2026-02-23

**Authors:** Rainer Wiechert, Leander Geske, Jasmin Hammes, Dogus Tuncer, Till Opatz

**Affiliations:** Department of Chemistry, 9182 Johannes Gutenberg University Mainz, Duesbergweg 10-14, Mainz 55128, Germany

**Keywords:** Alkaloids, Photoredox catalysis, Total synthesis, Cascade cyclization, Radical reactions

## Abstract

A concise route toward (−)-strychnine is presented. Key steps include the photoredox-catalytic C2-cyanomethylation of Boc-l-Trp-OMe and a condensation-electrocyclization cascade linking two fragments convergently to obtain an advanced intermediate. A photochemical decarboxylation provided convenient access to strychnofluorine, which could be transformed through the Wieland–Gumlich aldehyde to strychnine. All building blocks can be traced back to renewable sources.

## Graphical abstract

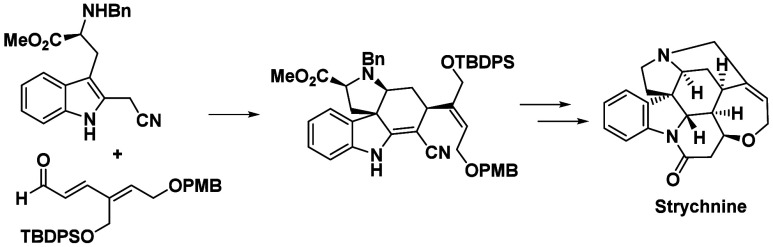

Since its landmark total synthesis by Woodward in 1954,^1^ strychnine (**1**) holds a special place in synthetic organic chemistry. Although applications of strychnine (**1**) in medicine, as a pharmacological tool compound or as a rodenticide, are rather limited at present,^2^ the molecule still represents a formidable synthetic target. The highly compact polycyclic structure and numerous stereocenters of this natural product motivated numerous groups to propose and develop routes based on innovative approaches.^3–35^ In more recent total syntheses of strychnine, the efficiency and practicality of novel methodologies have been evaluated by including them in the total synthesis of this complex molecule.^9,28,32,33,35^ Inspired by the possibilities of photoredox chemistry and the option of cascade reactions that could construct several stereocenters in a single transformation, we devised an enantioselective route toward (−)-strychnine (**1**) based on these two key transformations. Retrosynthetically, the natural product was traced back to Wieland–Gumlich aldehyde (**2**), a frequently employed synthetic intermediate. The latter could be obtained from a 6–5–6–5 tetracyclic precursor **3** through functional group manipulations and the latter could be assembled from the two main precursors **4** and **5** through a condensation sigmatropic rearrangement cascade first employed by Kuehne in his 1993 strychnine synthesis (Scheme 1). The inclusion of a side chain containing all carbon atoms of the eastern half of **1** in the cascade cyclization should result in a reduced overall step count and in a more convergent strategy, since the elongation of the formyl group on C-15 into the C-18 to C-21 eastern unit is avoided. The indole building block **4** could be readily available from l-tryptophan (**8**) through photochemically induced C2 indole alkylation. We hypothesized that a nitrile could be a good precursor to the aldehyde functionality in **2**. The dienal **5** could be conveniently generated by the Heck reaction to vinyl iodide **6** available from butynediol **7** (Scheme 2).

**Scheme 1:**
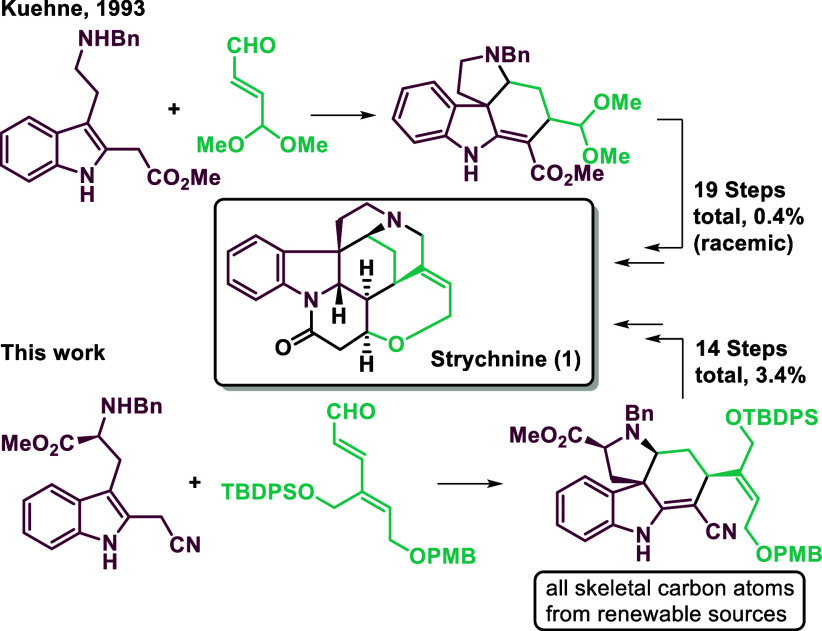
Previous Strategies for the Total Synthesis of Strychnine

**Scheme 2:**
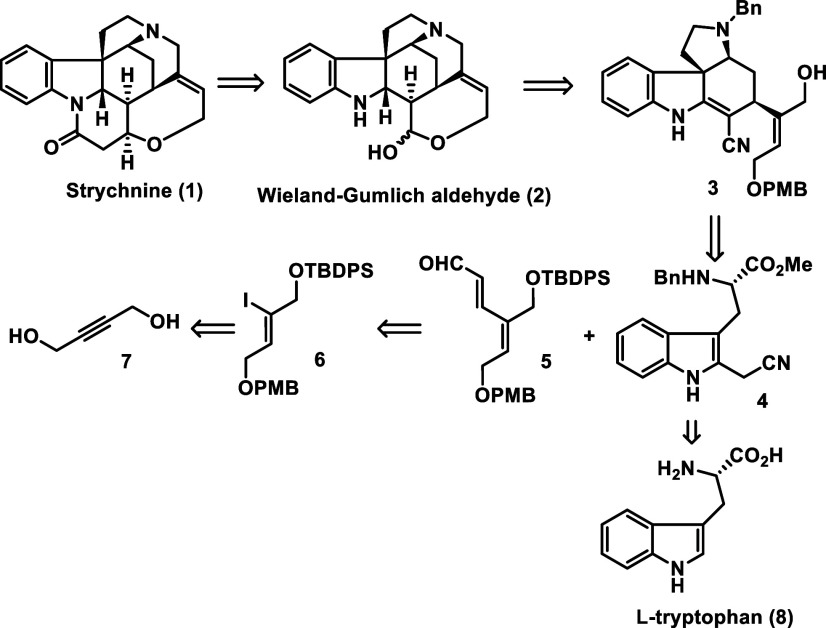
Retrosynthetic Analysis of (−)-Strychnine (**1**)

To obtain indole building block **4**, the photoredox-catalyzed C2 cyanomethylation of Bn-l-Trp-OMe was attempted under various conditions using catalysts containing or devoid of transition metals.^36–40^ The reactivity of the intermediate acetonitrile radical, paired with the nucleophilicity of the benzyl protected amine, resulted in side reactions, catalyst deactivation, and poor yields. We therefore utilized the Boc protected tryptophan methyl ester **9** and found that with [Ir­(dtbppy)_2_(dtbpy)]­PF_6_ and bromoacetonitrile under blue light irradiation cyanomethylation proceeded smoothly and delivered compound **10** in 76% yield on multigram scale. The reaction was also scalable to decagram amounts in one batch and utilizes only 0.1% of the iridium-catalyst. Deprotection and reductive amination with benzaldehyde produced the first key component **4** in 92% yield (67% from l-tryptophan (**8**) without loss of stereochemical integrity).^41^ For the second building block, TBDPS protected butynediol **12** was converted to the corresponding vinyl iodide **13** by treatment with Red-Al at cryogenic temperatures and subsequent trapping with NIS,^42,43^ followed by PMB protection to **6** in 97% yield. The synthesis of the second building block was finalized by ligand free Heck reaction^44^ of iodoolefin **6** with acrolein affording dienal **5** in 71% (Scheme 3).

**Scheme 3:**
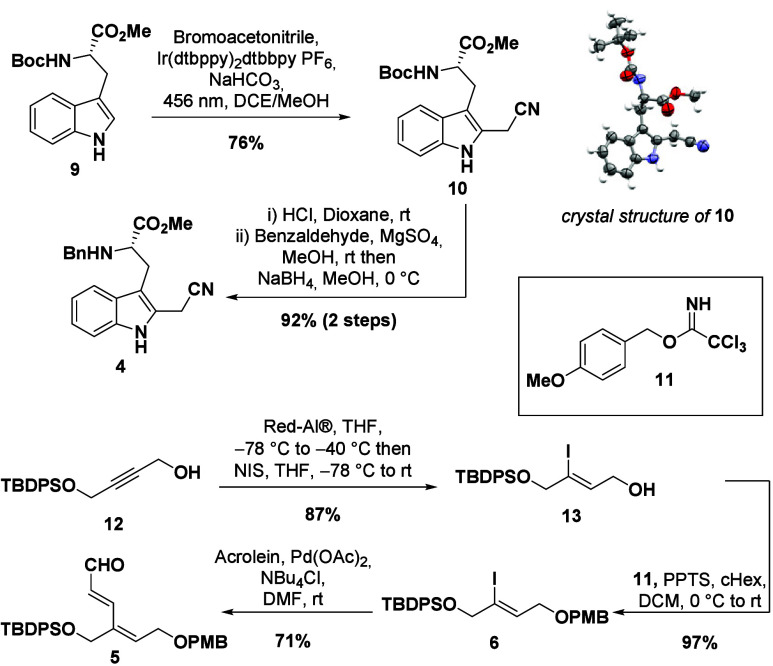
Preparation of the Indole and Unsaturated Aldehyde Building Blocks **4** and **5**

With both building blocks **4** and **5** in hand, we utilized reaction conditions by Kuehne^11,45^ to produce the tetracycle (*E*)-**14** in 49% yield and its double bond isomer (*Z*)-**14** in 14%, with full stereocontrol regarding positions 3, 7, and 15. Tetracycle (*E*)-**14** already comprises the full ABCE tetracyclic carbon framework and contains all necessary carbons to form the D- and F-rings of the final product. Attempts to further limit the production of undesired C19–C20 (*Z*)-isomer (*Z*)-**14** were unsuccessful. Based on control experiments, aldehyde **5** isomerizes to its (*Z*)-isomer by the addition of the indole through iminium formation and bond rotation. This process was more rapid than the subsequent cyclization cascade under all tested conditions, and the yield of (*Z*)-**14** reflects the efficiency of the chromatographic isolation rather than its formation in roughly equimolar amounts. Nevertheless, (*Z*)-**14** served as a perfect test substrate for further optimization studies (see the Supporting Information for details). The obtained conditions were transferred with minor changes to the desired (*E*)-isomer. Hydrolysis of the ester afforded acid **15** in 75% yield and photochemical decarboxylation under oxidative conditions gave compound **16** in 82% yield (Scheme 4).

**Scheme 4:**
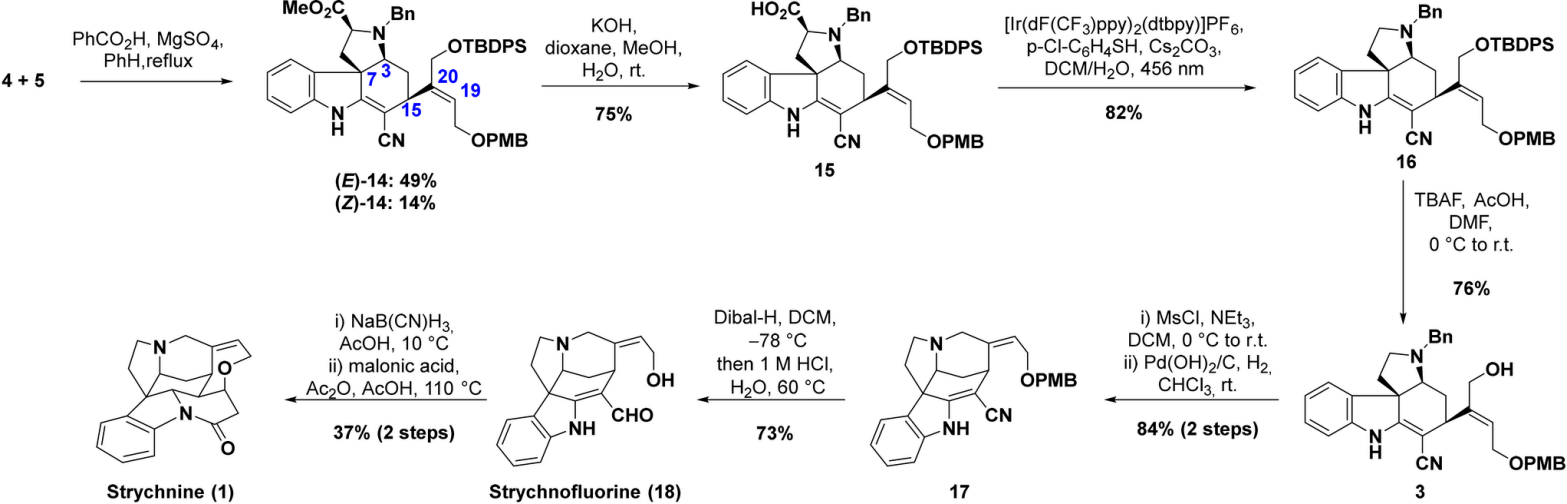
Completion of the Total Synthesis^a^

a Abbreviations: TBAF, tetrabutylammonium fluoride; Dibal-H, diisobutylaluminum hydride.

In order to close the D-ring, desilylation, followed by activation with MsCl produced the quaternary ammonium salt, which was debenzylated utilizing H_2_/Pd­(OH)_2_ in CHCl_3_. The selection of solvent and reaction time control in this transformation was crucial, since polar solvents or increased reaction times led to double bond reduction or even allylic ether cleavage. Finally, conversion of enamino nitrile **17** to γ-amino aldehyde **18** was attempted. A sequence by Qin et al.^30^ which reduces the enamine and Pinner reaction could yield a known strychnine precursor. In our case, the reduction only gave unsatisfactory yields, and the Pinner conditions only resulted in PMB deprotection without conversion of the nitrile. Instead, reduction of the nitrile and acidic cleavage of the PMB protection group gave strychnofluorine (**18**) in 73% yield.

We also hypothesized that the hemiacetal formation during enamine reduction toward the Wieland–Gumlich aldehyde (**2**) could suppress formation of the undesired C-16 epimer. Strychnofluorine (**18**) was reduced with sodium cyanoborohydride in acetic acid, yielding the Wieland–Gumlich aldehyde, contaminated with the diol overreduction product, which was not purified and was instead directly converted to (−)-strychnine by the method of Robinson.^46^

Overall, we report a concise total synthesis of strychnine. Starting from l-tryptophan, 14 linear steps were required, and the overall yield amounted to 3.4%. All skeletal atoms of the final product can be traced back to xylochemicals or other renewable resources, including the fermentation product l-tryptophan: bromoacetonitrile (available from acetic acid),^47,48^ butynediol (from biochar-derivable acetylene^49^ and formaldehyde),^50^ acrolein (glycerol dehydration),^51^ and malonic acid (fermentation).^52^ A combination of an optimized photochemical indole C2-cyanomethylation and Kuehne’s cascade-Mannich electrocyclization with an advanced aldehyde building block reduced the overall step count of the synthesis, while the light-driven steps were superior to more classical approaches in terms of step count and yield. We hope this report will inspire further works in the field of alkaloid total synthesis.

## Supplementary Material



## Notes

Detailed experimental procedures, spectra, and X-ray data (PDF)

## †

Bachem, 4416 Bubendorf, Switzerland

## Notes

Dedicated to Professor Albert (Al) Padwa, mentor and friend.

## Abbreviations

DCE: 1,2-dichloroethane
rt: room temperature
Red-Al: sodium bis­(2-methoxyethoxy)­aluminum hydride
NIS: *N*-iodosuccinimide
PPTS: pyridinium 4-methylbenzenesulfonate
cHex: cyclohexane

## References

[1] Woodward R. B., Cava M. P., Ollis W. D., Hunger A., Daeniker H. U., Schenker K. (1954). The Total Synthesis of Strychnine. J. Am. Chem. Soc..

[2] Philippe G., Angenot L., Tits M., Frédérich M. (2004). About the
Toxicity of Some Strychnos Species and Their Alkaloids. Toxicon.

[3] Woodward R. B., Cava M. P., Ollis W. D., Hunger A., Daeniker H. U., Schenker K. (1963). The Total Synthesis of Strychnine. Tetrahedron.

[4] Magnus P., Giles M., Bonnert R., Kim C. S., McQuire L., Merritt A., Vicker N. (1992). Synthesis of Strychnine
via the Wieland-Gumlich
Aldehyde. J. Am. Chem. Soc..

[5] Stork, G. Totalsynthese von Strychnin. In Ischia Porto Advanced School of Organic Chemistry, September; Italien, 1992.

[6] Magnus P., Giles M., Bonnert R., Johnson G., McQuire L., Deluca M., Merritt A., Kim C. S., Vicker N. (1993). Synthesis
of Strychnine and the Wieland-Gumlich Aldehyde. J. Am. Chem. Soc..

[7] Knight S. D., Overman L. E., Pairaudeau G. (1993). Enantioselective
Total Synthesis
of (−)-Strychnine. J. Am. Chem. Soc..

[8] Knight S. D., Overman L. E., Pairaudeau G. (1995). Asymmetric
Total Syntheses of (−)-
and (+)-Strychnine and the Wieland—Gumlich Aldehyde. J. Am. Chem. Soc..

[9] Kuehne M. E., Xu F. (1993). Total Synthesis of
Strychnan and Aspidospermatan Alkaloids. 3. The
Total Synthesis of (±)-Strychnine. J. Org.
Chem..

[10] Rawal V. H., Iwasa S. (1994). A Short, Stereocontrolled Synthesis of Strychnine. J. Org. Chem..

[11] Kuehne M. E., Xu F. (1998). Syntheses of Strychnan-
and Aspidospermatan-Type Alkaloids. 10. An
Enantioselective Synthesis of (−)-Strychnine through the Wieland-Gumlich
Aldehyde. J. Org. Chem..

[12] Solé D., Bonjoch J., García-Rubio S., Peidró E., Bosch J. (1999). Total Synthesis of (−)-Strychnine
via the Wieland-Gumlich
Aldehyde. Angew. Chem., Int. Ed..

[13] Sole D., Bonjoch J., García-Rubio S., Peidro E., Bosch J. (2000). Enantioselective
Total Synthesis of Wieland-Gumlich Aldehyde and (−)-Strychnine. Chem. - Eur. J..

[14] Eichberg M. J., Dorta R. L., Lamottke K., Vollhardt K. P. C. (2000). The
Formal Total Synthesis of (±)-Strychnine via a Cobalt-Mediated
[2 + 2 + 2]­Cycloaddition. Org. Lett..

[15] Ito M., Clark C. W., Mortimore M., Goh J. B., Martin S. F. (2001). Biogenetically
Inspired Approach to the Strychnos Alkaloids. Concise Syntheses of
(±)-Akuammicine and (±)-Strychnine. J. Am. Chem. Soc..

[16] Eichberg M. J., Dorta R. L., Grotjahn D. B., Lamottke K., Schmidt M., Vollhardt K. P. C. (2001). Approaches
to the Synthesis of (±)-Strychnine
via the Cobalt-Mediated [2 + 2 + 2] Cycloaddition: Rapid Assembly
of a Classic Framework. J. Am. Chem. Soc..

[17] Nakanishi M., Mori M. (2002). Total Synthesis
of (−)-Strychnine. Angew.
Chem., Int. Ed..

[18] Bodwell G. J., Li J. (2002). A Concise Formal Total
Synthesis of (AE)-Strychnine by Using a Transannular
Inverse-Electron-Demand Diels ± Alder Reaction of a [3]­(1,3)­Indolo[3]­(3,6)­Pyridazinophane**. Angew. Chem., Int. Ed..

[19] Ohshima T., Xu Y., Takita R., Shimizu S., Zhong D., Shibasaki M. (2002). Enantioselective
Total Synthesis of (−)-Strychnine Using the Catalytic Asymmetric
Michael Reaction and Tandem Cyclization. J.
Am. Chem. Soc..

[20] Mori M., Nakanishi M., Kajishima D., Sato Y. (2003). A Novel and General
Synthetic Pathway to Strychnos Indole Alkaloids: Total Syntheses of
(−)-Tubifoline, (−)-Dehydrotubifoline, and (−)-Strychnine
Using Palladium-Catalyzed Asymmetric Allylic Substitution. J. Am. Chem. Soc..

[21] Kaburagi Y., Tokuyama H., Fukuyama T. (2004). Total Synthesis
of (−)-Strychnine. J. Am. Chem. Soc..

[22] Zhang H., Boonsombat J., Padwa A. (2007). Total Synthesis of (±)-Strychnine
via a [4 + 2]-Cycloaddition/ Rearrangement Cascade. Org. Lett..

[23] Sirasani G., Paul T., Dougherty W., Kassel S., Andrade R. B. (2010). Concise
Total Syntheses of (±)-Strychnine and (±)-Akuammicine. J. Org. Chem..

[24] Beemelmanns C., Reissig H. U. (2010). A Short Formal Total
Synthesis of Strychnine with a
Samarium Diiodide Induced Cascade Reaction as the Key Step. Angew. Chem., Int. Ed..

[25] Martin D. B. C., Vanderwal C. D. (2011). A Synthesis
of Strychnine by a Longest Linear Sequence
of Six Steps. Chem. Sci..

[26] Jones S. B., Simmons B., Mastracchio A., MacMillan D. W. C. (2011). Collective
Synthesis of Natural Products by Means of Organocascade Catalysis. Nature.

[27] Jacquemot G., Maertens G., Canesi S. (2015). Isostrychnine
Synthesis Mediated
by Hypervalent Iodine Reagent. Chem. - Eur.
J..

[28] Feng L. W., Ren H., Xiong H., Wang P., Wang L., Tang Y. (2017). Reaction of
Donor-Acceptor Cyclobutanes with Indoles: A General Protocol for the
Formal Total Synthesis of (±)-Strychnine and the Total Synthesis
of (±)-Akuammicine. Angew. Chem., Int.
Ed..

[29] Lee G. S., Namkoong G., Park J., Chen D. Y. K. (2017). Total Synthesis
of Strychnine. Chem. - Eur. J..

[30] He L., Wang X., Wu X., Meng Z., Peng X., Liu X. Y., Qin Y. (2019). Asymmetric
Total Synthesis of (+)-Strychnine. Org. Lett..

[31] Hutchings-Goetz L. S., Yang C., Fyfe J. W. B., Snaddon T. N. (2020). Enantioselective
Syntheses of Strychnos and Chelidonium Alkaloids through Regio- and
Stereocontrolled Cooperative Catalysis. Angew.
Chem., Int. Ed..

[32] Wang P., Chen J., He W., Song J., Song H., Wei H., Xie W. (2021). An Asymmetric
Synthesis of (+)-Isostrychnine Based
on Catalytic Asymmetric Tandem Double Michael Addition. Org. Lett..

[33] Liu X., Lou M., Bai S., Sun G., Qi X. (2022). Asymmetric Total Syntheses
of Strychnos Alkaloids via Selective Fischer Indolization. J. Org. Chem..

[34] Zhao L. P., Zhang S. Y., Liu H. K., Cheng Y. J., Liu Z. P., Wang L., Tang Y. (2023). Insights into
Stereoselectivity Switch
in Michael Addition-Initiated Tandem Mannich Cyclizations and Their
Extension from Enamines to Vinyl Ethers. J.
Am. Chem. Soc..

[35] Zhou W., Xi S., Chen H., Jiang D., Yang J., Liu S., He L., Qiu H., Lan Y., Zhang M. (2023). A Bridged Backbone
Strategy Enables Collective Synthesis of Strychnan Alkaloids. Nat. Chem..

[36] Nebe M. M., Loeper D., Fürmeyer F., Opatz T. (2018). Visible-Light Organophotoredox-Catalyzed
Synthesis of Precursors for Horner-Type Olefinations. Eur. J. Org. Chem..

[37] Furst L., Matsuura B. S., Narayanam J. M. R., Tucker J. W., Stephenson C. R. J. (2010). Visible
Light-Mediated Intermolecular C-H Functionalization of Electron-Rich
Heterocycles with Malonates. Org. Lett..

[38] O’Brien C. J., Droege D. G., Jiu A. Y., Gandhi S. S., Paras N. A., Olson S. H., Conrad J. (2018). Photoredox Cyanomethylation of Indoles:
Catalyst Modification and Mechanism. J. Org.
Chem..

[39] Laroche B., Tang X., Archer G., Di Sanza R., Melchiorre P. (2021). Photochemical
Chemoselective Alkylation of Tryptophan-Containing Peptides. Org. Lett..

[40] Liu Z.-Q., Li Z. (2016). Radical-Promoted Site-Specific Cross
Dehydrogenative Coupling of
Heterocycles with Nitriles. Chem. Commun..

[41] Deposition Number 2481414 contains the supplementary crystallographic data for this paper. These data can be obtained free of charge via the joint Cambridge Crystallographic Data Centre (CCDC) and Fachinformationszentrum Karlsruhe Access Structures service.

[42] Overman L. E., Rosen M. D. (2000). Total Synthesis of (−)-Spirotryprostatin B and
Three Stereoisomers. Angew. Chem., Int. Ed..

[43] Corey E. J., Katzenellenbogen J. A., Posner G. H. (1967). New Stereospecific Synthesis of Trisubstituted
Olefins. Stereospecific Synthesis of Farnesol. J. Am. Chem. Soc..

[44] Jeffery T. (1984). Palladium-Catalysed
Vinylation of Organic Halides under Solid-Liquid Phase Transfer Conditions. J. Chem. Soc., Chem. Commun..

[45] Kuehne M. E., Xu F. (1997). Syntheses of Strychnan-
and Aspidospermatan-Type Alkaloids. 9. ^1^ The Enantioselective
Generation of Tetracyclic ABCE Intermediates
by a Tandem Condensation, [3,3]-Sigmatropic Rearrangement, and Cyclization
Sequence. J. Org. Chem..

[46] Robinson R., Anet F. A. L. (1953). Conversion of
the Wieland-Gumlich Aldehyde into Strychnine. Chem. Ind..

[47] Guoguo, S. ; Xicai, L. ; Peng, L. ; Bin, W. ; Dapeng, C. Preparation Method of Bromoacetonitrile. CN114591199A, 2022.

[48] Galanov S.
I., Sidorova O. I., Gavrilenko M. A. (2014). The Process of Acetonitrile Synthesis
over γ-Al2O3 Promoted by Phosphoric Acid Catalysts. Procedia Chem..

[49] Jiang P., Zhao G., Zhang H., Ji T., Mu L., Lu X., Zhu J. (2024). Towards Carbon Neutrality of Calcium
Carbide-Based
Acetylene Production with Sustainable Biomass Resources. Green Energy & Environment.

[50] Said A. E.-A. A., Goda M. N. (2019). Synthesis, Characterization
and Catalytic Activity
of Nanocrystalline Ce2­(MoO4)­3/SiO2 as a Novel Catalyst for the Selective
Production of Anhydrous Formaldehyde from Methanol. Catal. Lett..

[51] Corma A., Huber G., Sauvanaud L., Oconnor P. (2008). Biomass to Chemicals:
Catalytic Conversion of Glycerol/Water Mixtures into Acrolein, Reaction
Network. J. Catal..

[52] Turner W. A., Hartman A. M. (1925). The Non-Volatile
Organic Acids of Alfalfa. J. Am. Chem. Soc..

