# Supplementary material for: A Total Synthesis of (−)-Strychnine Using Photoredox Catalysis
Source: JACS Au. 2026 Feb 23;6(3):1822–6. doi: 10.1021/jacsau.5c01709 (PMC13014235; doi:10.1021/jacsau.5c01709)
Supplement: Supplementary file 1 [file au5c01709_si_001.pdf]

Supporting Information

A Total Synthesis of (−)-Strychnine Using Photoredox Catalysis

Rainer Wiechert, Leander Geske, Jasmin Hammes, Dogus Tuncer, Till Opatz*

Department of Chemistry, Johannes Gutenberg University Mainz, Duesbergweg 10–14, Mainz, Germany

† Present address: Bachem, 4416 Bubendorf, Switzerland

Table of Contents

General Information S3

Reported total syntheses S5

Additional Information & reaction optimizations S6

Optimization of reaction conditions with undesired isomer S9

Debenzylation............ S12

Schemes of unsuccessful attempts S12

Endgame............ S13

Procedures and Compound Characterizations............ S14

Synthesis of **10**............ S14

Synthesis of **4**............ S15

Synthesis of **12**............ S15

Synthesis of **13**............ S16

Synthesis of **6**............ S17

Synthesis of compound **5** S18

Synthesis of **14**............ S19

Synthesis of **15**............ S21

Synthesis of **16**............ S22

Synthesis of **3**............ S23

Synthesis of **17**............ S24

Synthesis of **18**............ S25

Table NMR comparison of Strychnofluorine (**18**)............ S25

Synthesis of strychnine (**1**) S26

Synthesis of **S3**............ S30

Synthesis of **S4**............ S31

Synthesis of **S7b**............ S32

Synthesis of **S5**............ S33

Synthesis of **S6**............ S34

Synthesis of S7............ S34

Synthesis of **S8**............ S35

Synthesis of **S9**............ S36

Synthesis of **S10**............ S37

Synthesis of **S11** and **S12**............ S38

Synthesis of **S15**............ S39

References............ S41

Spectra............ S46

General Information

Solvents and reagents

Unless stated otherwise, all solvents and reagents were obtained from commercial suppliers and used without prior purification. Solvents used for photoreactions were degassed for 10 minutes by sparging with argon. Anhydrous THF, *N*,*N*-dimethylacetamide, MeOH, DCE and EtOH were purchased from commercial suppliers as extra dry solvents stored over 3 Å molecular sieves and used without additional purification. Anhydrous dichloromethane, THF, and MeCN were used from an SPS5 solvent purification system (MBRAUN, Garching, Germany). Solvent mixtures of acetonitrile/water were removed by freeze-drying on an Alpha 2-4 LDplus freeze-drying system (Martin Christ, Osterode am Harz, Germany).

General performance of reactions

Water and air sensitive reactions were performed under nitrogen or argon atmosphere in pre-dried glassware utilizing standard Schlenk techniques. Liquid reagents or solvents were added through septa.

Cooled reactions were cooled with standard cooling mixtures (ice + water (1:1) for 0 °C; acetone or iPrOH + dry ice for −78 °C) or an Immersion cooler with temperature probe FT902 (Julabo, Seelbach, Germany) was used.

All reactions that required heating were heated with an aluminum heating mantle placed on a stir plate.

The stated temperatures in the procedure sections correspond to the temperature of the heating mantle or cooling solution. In case reflux is stated, the temperature is adjusted so that boiling and condensation of the solvent is observed.

Reaction control was performed utilizing TLC, HPLC/MS or ^{1}H-NMR spectroscopy.

Chromatography

Preparative column chromatography was performed either on silica gel (35–70 µm, Acros Organics) using an overpressure of 0.5–0.6 bar nitrogen with the stated solvent mixtures as v/v rations.

Thin-layer chromatography (TLC) was carried out on silica plates (TLC Silica 60 F254 by Merck KGaA). Visualization of the compounds was accomplished by illumination with UV-light of the developed plates and by staining with $KMnO_4$ or Seebach´s reagent.

NMR spectra

NMR spectra were recorded on a Bruker Avance-III HD (^{1}H-NMR: 300 MHz, ^{13}C-NMR: 75.5 MHz), a Bruker Avance-II (^{1}H-NMR: 400 MHz, ^{13}C-NMR: 100.6 MHz) or a Avance III 600 (^{1}H-NMR: 600 MHz, ^{13}C-NMR: 150.9 MHz) spectrometer. Chemical shifts are referenced to residual solvent

signals ($CDCl_3$: 7.26 ppm and 77.16 ppm, DMSO-d_6: 2.50 ppm and 39.52 ppm acetonitrile-d_3: 1.94 ppm, 118.7 ppm for ^{1}H-NMR and ^{13}C-NMR, respectively) and reported in parts per million (ppm) relative to tetramethylsilane (TMS). Multiplicities of NMR signals are abbreviated as follows: br = broad, s = singlet, d = doublet, t = triplet, q = quartet, m = multiplet and combinations thereof, app = apparent.

Mass spectra

Electrospray ionization (ESI) mass spectra were recorded on a 1200-series HPLC- system (Agilent-Technologies) with binary pump and integrated diode array detector coupled to an Agilent Infinity Lab 6100 Series LCMSD (G6125B). High resolution mass spectra were recorded on an Agilent G6545A Q-Tof with ESI, APCI, or APPI source coupled with an Agilent 1260 Infinity II HPLC system.

Analytical and preparative HPLC

For analytical HPLC separation, an Agilent Technologies 1260 Infinity II system including a Macherey-Nagel (MN) Nucleodur C_{18}-HTEC-column (particle size: 5 µm, length: 150 mm, diameter: 4.6 mm, column temperature: 40 °C, flow rate: 1 mL/min) or an Avantor ACE 3 18-PFP (particle size: 3 µm, length: 150 mm, diameter: 4.6 mm, column temperature: 40 °C, flow rate: 1 mL/min) with UV-DAD detection was used.

Preparative HPLC was performed on an Agilent Technologies 1290 Infinity II system with two high-pressure gradient K-1800 pumps and an S-260-UV-DAD detector. The separation took place on a MN Nucleodur C_{18}-HTEC (particle size: 5 µm, length: 150 mm, diameter: 32 mm, flow rate: 42.5 mL/min)

For both analytical and preparative HPLC, the eluent mixtures of solvents A: H_2O (LCMS grade or Milli-Q filtered) + 0.1% formic acid (LCMS grade) and B: MeCN (HPLC and LCMS grade) were given as a v/v ratio.

Chiral analytical HPLC was performed on an Agilent Technologies 1290 Infinity II system with isocratic pump, degasser, automatic liquid injector and diode array detector. The measurements were performed at 40 °C with a flowrate of 1 mL/min of an isocratic mixture of *n*-hexane (HPLC grade) and EtOH (HPLC grade). As stationary phase, a Daicel Chiralpak® IF-3 column (particle size: 3 µm, dimensions: 250 mm x 4.6 mm) was used.

IR-Spectra

IR spectra were recorded on a Tensor 27 spectrometer with a diamond ATR unit from Bruker. The measured spectra were analyzed using the Opus 7.2 software from Bruker. In the following, only the ten most intense bands are given together with characteristic bands.

Polarimetry

Optical rotation measurements were accomplished with a Perkin-Elmer 241 MC polarimeter at λ = 589 nm in a quartz cell (length 10 cm, V = 1 mL). A solvent-filled cuvette was used for instrument calibration.[1]

Melting Points

Melting ranges were determined in open glass capillaries on a melting point measuring device type MP30 from Mettler Toledo.

Photochemical Setup

Reactions were performed using the Kessil (Richmond, CA 94804) *PR160L-456nm* lamp (maximum at 456 nm) and cooling was employed using a fan. The lamps were used at the stated intensity setting (25%, 50%, 75% or full intensity).

The set-up was as shown, the reaction vessel was placed at a distance of approx. 2 cm from both light sources:

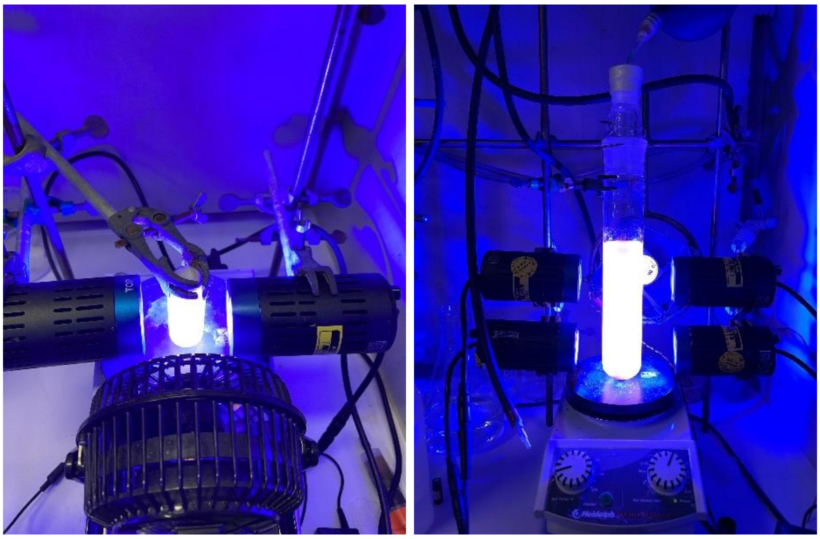

Figure S1: Example reaction set-up for the photochemical reactions (10 mmol and 100 mmol scale).

Reported total syntheses

Table S 1: Overview of reported total syntheses of strychnine.

N#	Name	Year	Target	Precursor	Steps	Yield[a]	Sequence
1	Woodward[2,3]	1954 /1963	(−)	Iso	29	<0.01%	A-B-C-G-E-D-F
2	Magnus[4]	1992	(±)	WG	28	0.03%	AB-D-CE-F-G
3	Stork[5]	1992	(±)	WG	14	nr	AB-CE-D-F-G
4	Magnus[6]	1993	(−)	WG	28	0.03%	AB-D-CE-F-G
5	Overman[7,8]	1993<br>1995	(−)<br>(−) & (+)	WG	24<br>24	2.4%<br>4% & nr	A-D-CE-B-F-G
6	Kuehne[9]	1993	(±)	Iso	19	0.4%	AB-CE-D-G-F
7[‡]	Rawal[10]	1994	(±)	Iso	12(+1)	35% (10%)	A-C-E-G-D-F
8	Kuehne[11]	1998	(−)	WG	22	3%	AB-CE-D-F-G
9	Bonjoch[12,13]	1999	(−)	WG	15	0.2%	AE-C-D-B-F-G
10[‡]	Vollhardt[14,15]	2000	(±)	Iso	13 (+1)	2.8% (0.6%)	AB-EG-C-D-F

11‡	Martin[16]	2001	(±)	WG	12 (+4)	2.8% (1%)	AB-D-CE-F-G
12	Mori[17,18]	2002	(−)	Iso	22	0.07%	E-A-B-C-G-D-F
13‡	Bodwell[19]	2002	(±)	Iso	8(+4)	16% (3%)	AB-CEG-D-F
14	Shibasaki[20]	2002	(−)	WG	31	0.3%	E-A-BD-C-F-G
15	Fukayama[21]	2004	(−)	WG	25	1%	A-B-D-CE-F-G
16	Padwa[22]	2007	(±)	WG	17	2%	AB-CE-D-F-G
17	Andrade[23]	2010	(±)	WG	13	5%	AB-CE-D-F-G
18‡	Reissig[24]	2010	(±)	Iso	8(+2)	15% (4%)	AB-EG-C-D-F
19‡	Vanderwal[25]	2011	(±)	WG	5(+1)	4% (3%)	AB-CE-DF-G
20	MacMillan[26]	2011	(−)	WG	12	7%	AB-CE-DF-G
21‡	Canesi[27]	2015	(±)	Iso	10(+1)	1.6% (0.4%)	AE-G-B-C-D-F
22‡	Wang/Tang[28]	2017	(±)	WG	12(+4)	4.6% (1.1%)	AB-E-C-DF-G
23	Chen[29]	2017	(−)	WG	10	3.5%	C-A-B-E-D-F-G
24	Qin[30]	2018	(+)	WG	17	0.1%	A-C-BD-F-G
25	Snaddon[31]	2020	(−)	WG	9	4.3%	AB-CE-D-F-G
26‡	Xie/Wei[32]	2021	(−)	Iso	9(+1)	16% (4.5%)	AB-C-E-G-D-F
27	Qi[33]	2022	(−)	Iso	13 (+4)	2.1% (0.1%)	EC-AB-G-D-F
28‡	Tang/Wang[34]	2023	(+)	WG	6(+3)	27% (7%)	AB-CE-DF-G
29	Zhang/Lan[35]	2023	(−)	WG	13	2.3%	E-D-AB-C-F-G
30	Wiechert/Opatz	2025	(−)	WG	14	3.4%	AB-CE-D-F-G

‡/a formal total synthesis, steps and yield towards strychnine in brackets. WG: Wieland-Gumlich aldehyde. Iso: Isostrychnine. nr: not reported.

Additional Information & reaction optimizations

Optimization of photoreaction

Table S 2: Optimization of photochemical 2-cyanomethylation of Boc-Trp-OMe.

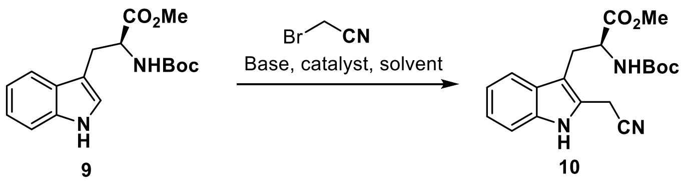

N#	Scale/ mmol	Catalyst (mol%)	Base	Solvent	C / mol/L	t/h	Lamps[a]	Yield (% conv.[b])
1	0.17	Eosin Y (5)	2,6-Lutidine	DCM	0.06	48	P=34, 1	(33)
2	0.17	Eosin Y (5)	$NaHCO_3$	DMSO	0.17	24	P=34, 1	traces
3	0.35	[Ir(dF(CF$_3$)ppy$_2$dtbbpy]PF$_6$ (2)	$NaHCO_3$	DMSO	0.17	24	P=34, 1	(53)
4	0.35	[Ir(dF(CF$_3$)ppy$_2$dtbbpy]PF$_6$ (2)	$NaHCO_3$	DMSO	2	24	P=34, 1	(71)

5	0.35	$[Ir(dmppy)_2dtbbpy]PF_6$ (2)	$NaHCO_3$	DMSO	2	48	P=34, 1	78
6	0.35	$[Ir(dtbppy)_2dtbbpy]PF_6$ (2)	$NaHCO_3$	DMSO	2	24	P=34, 1	83
7	1.00	$[Ir(dtbppy)_2dtbbpy]PF_6$ (2)	$NaHCO_3$	DMSO	0.5	22	P=34, 2	34
8	1.00	$[Ir(dtbppy)_2dtbbpy]PF_6$ (2)	Na_2HPO_4	DMSO	0.5	18	P=50, 2,25%	31
9	1.00	$[Ir(dtbppy)_2dtbbpy]PF_6$ (2)	$NaHCO_3$	DCE/MeOH (1:1)	0.5	19	P=50, 2,25%	58
10	1.00	--	$NaHCO_3$	DCE/MeOH 1:1	0.5	72	P=50, 2, 25%	traces
11	1.00	$[Ir(dtbppy)_2dtbbpy]PF_6$ (2)	$NaHCO_3$	DCE/MeOH	0.5	72	--	(0)
12	2.80	$[Ir(dtbppy)_2dtbbpy]PF_6$ (2)	$NaHCO_3$	DCE/MeOH 1:1	0.5	72	P=50, 2, 25%	10
13	10.0	$[Ir(dtbppy)_2dtbbpy]PF_6$ (2)	$NaHCO_3$	DCE/MeOH 1:1	0.5	50	P=50, 2, 25%	52
14	10.0	$[Ir(dtbppy)_2dtbbpy]PF_6$ (1)	$NaHCO_3$	″	0.5	48	P=50, 2, 25%	48
15	10.0	$[Ir(dtbppy)_2dtbbpy]PF_6$ (0.1)	$NaHCO_3$	″	0.5	62	P=50, 2, 25%	54
16[c]	10.0	$[Ir(dtbppy)_2dtbbpy]PF_6$ (0.1)	$NaHCO_3$	″	0.5	72	P=50, 2, 25%	63
17[d]	10.0	$[Ir(dtbppy)_2dtbbpy]PF_6$ (0.1)	$NaHCO_3$	″	0.5	72	P=50, 2, 25%	74
18	100	$[Ir(dtbppy)_2dtbbpy]PF_6$ (0.1)	$NaHCO_3$	″	0.5	96	P=50, 4, 25%	51

[a] Power of lamps in W, number of lamps used, utilized power setting of lamps (% of max intensity) [b] conversion based on integral rations of HPLC chromatogram (λ=254 nm). [b] 3 eq. of bromoacetonitrile. [c] sequential addition of bromoacetonitrile (3 eq.).

<u>Cascade reaction</u>

Whilst performing the condensation cyclization reaction, we obtained two products of the same mass. NMR analysis showed that these products were C19-C20 *E/Z* isomers. To get further insights, the reaction conditions were varied. It was observed that compound **5** isomerized to its isomer **S1** already at room temperature in the presence of the indole building block. Formation of the desired cyclization product was however only observed at elevated temperatures. Treatment of the undesired isomer (*Z*)-**14** under the reaction conditions did not result in conversion to compound (*E*)-**14**. At even higher reaction temperatures, further side products such as compound **S2**, a *C*-3a,*C*-11b isomer, which was isolated after TBDPS deprotection of the diastereomeric mixture, were observed.

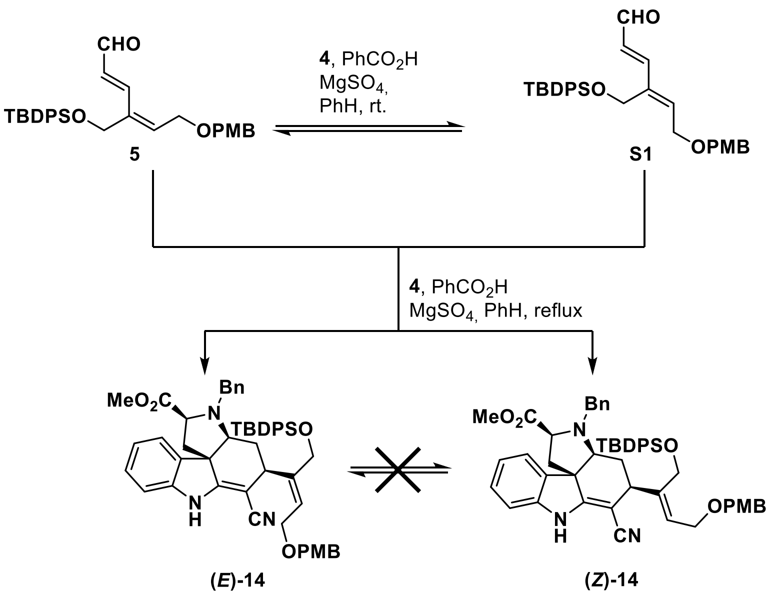

CHO
TBDPSO
OPMB
5
4, PhCO2H
MgSO4,
PhH, rt.
CHO
TBDPSO
OPMB
S1
4, PhCO2H
MgSO4, PhH, reflux
MeO2C
Bn
N
TBDPSO
N
H
CN
OPMB
(E)-14
MeO2C
Bn
N
TBDPSO
OPMB
N
H
CN
(Z)-14

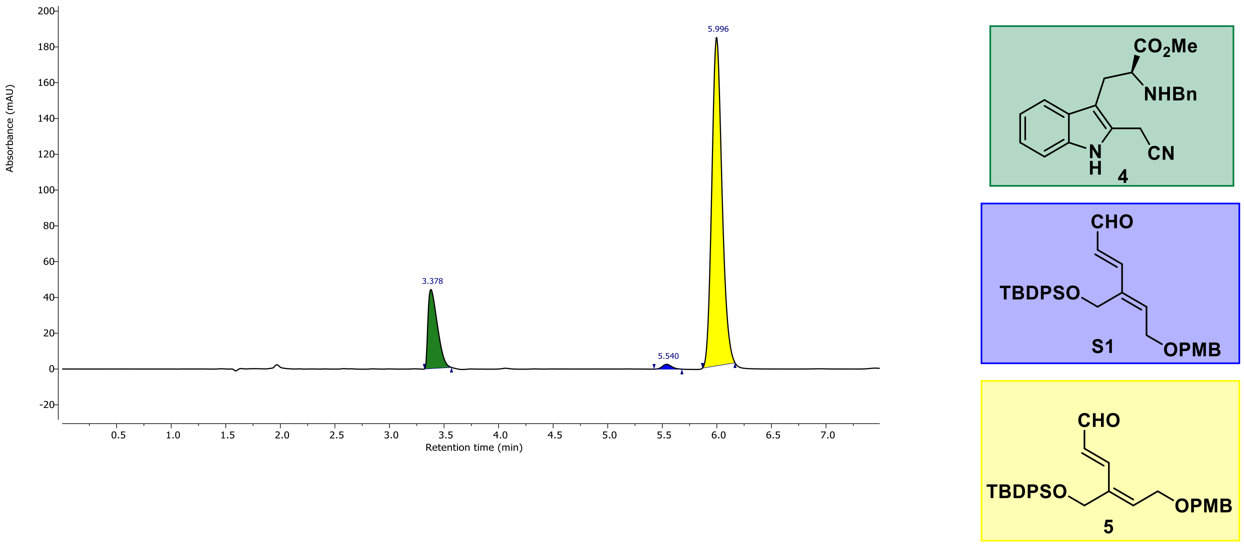

Absorbance (mAU)
Retention time (min)
3.378
5.540
5.996
CO2Me
NHBn
N
H
CN
4
CHO
TBDPSO
S1
OPMB
CHO
TBDPSO
OPMB
5

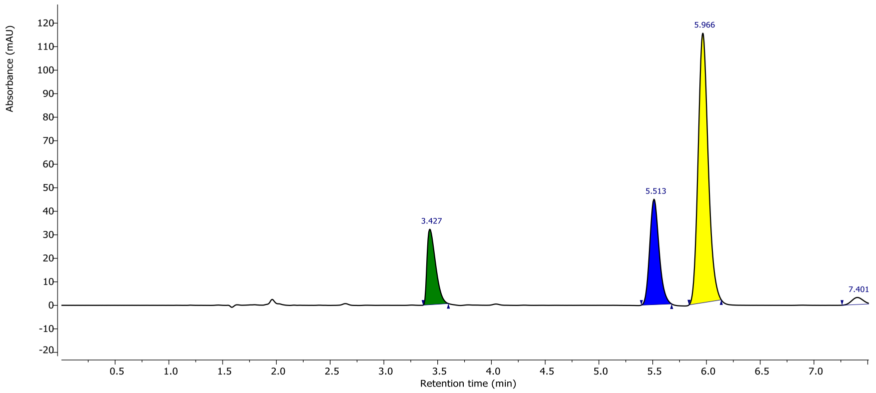

Figure S 1: Analysis of isomerization reaction of aldehyde **5** to compound **S1** in the presence of indole **4**. Conditions: indole (1 eq.), $PhCO_2H$ (1 eq.), $MgSO_4$ (2 eq.), PhH, rt. Samples after 1 h and 21 h. Absorbance at 254 nm, ACE3 C18-PFP (3 µm, 150x4.6), MeCN/H_2O 80:20.

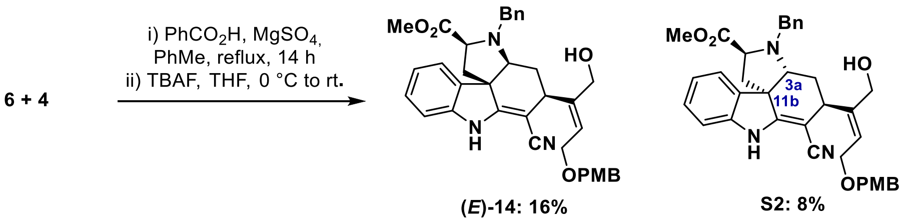

Optimization of reaction conditions with undesired isomer

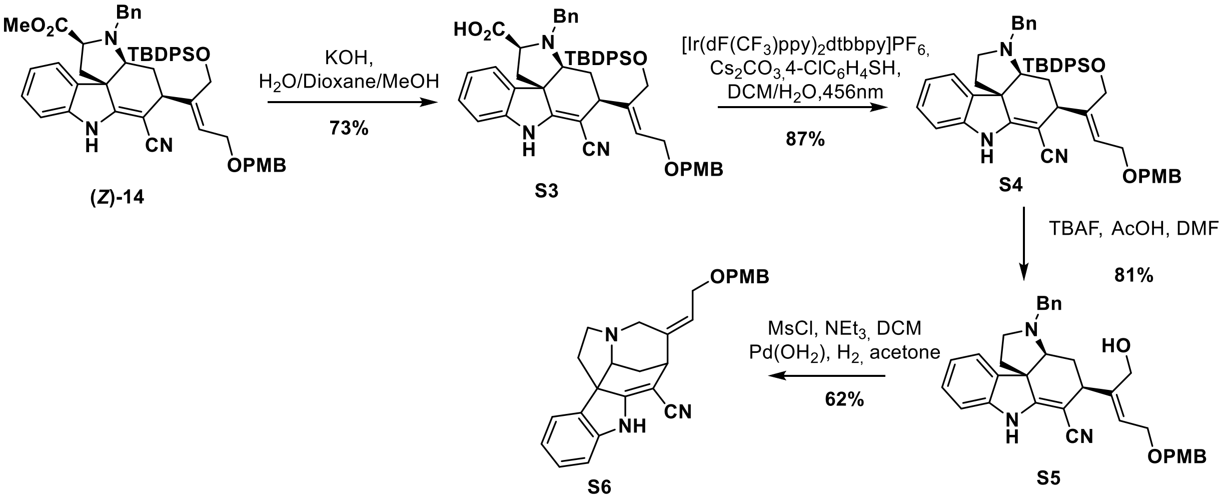

To test conditions for downstream reactions, we employed the obtained *C*-19/*C*-20 double bond isomer of the obtained tetracycle (*E*)**-14**. The orientation of the double bond should not have a large impact on the outcome of the centers of the reaction and therefore serves a test substrate. This should reduce the overall amount of substrate required for the optimizations.

Ester hydrolysis was achieved by utilizing a modified version of Tesser´s base (KOH in a ternary mixture of dioxane, MeOH and H_2O).[36] This mixture allowed full dissolution of the starting material and reagent. 2.5 equivalents of KOH were sufficient to cleave the ester selectively, whilst a large access also hydrolyzed the nitrile to its corresponding amide.

For hydrodecarboxylation, a variety of literature reported protocols were employed. Methods that directly decarboxylate carboxylic acids by excited state photocatalyst and an H-atom transfer reagent mostly gave the desired compound **S4**. Photocatalysts with very high oxidation potential led to decomposition or also cleaved the *N*-benzyl group. It was also attempted to convert the acid to redox active esters (NHPI and Barton) to decarboxylate by reductive methodologies. In most cases, this led to overreduced products or decomposition as was judged by LCMS analysis.

Silyl ether cleavage was achieved by the addition of TBAF. Buffered solutions with AcOH proved to be beneficial, whilst HF was ineffective in desilylation of the alcohol.

Treatment of alcohol **S5** with MsCl gave the tetraalkylammonium salt with closed D ring, which was reductively debenzylated with Pearlman's catalyst. In the debenzylation the choice of solvent was of importance since MeOH and AcOH showed less activity and more undesired hydrogenated side products were observed. Pearlman's catalyst was more active than Pd/C and non protic solvents increased conversion. THF and EtOAc could not be utilized since the solubility of the starting material was insufficient. Quaternization and subsequent hydrogenation was achieved in 62% yield.

Transfer of the so obtained conditions to the desired double bond isomer **(*E*)-14** was attempted. Ester hydrolysis conditions could be employed without changes. For the decarboxylation reduced reaction times were necessary. Instead of requiring 24 h, full conversion was observed after just 1 h and prolonged reaction times lead to decomposition of the formed product.

For construction of the D ring and debenzylation, the earlier obtained conditions were also effective. Finally, changing the solvent in the reductive debenzylation to chloroform further increased the yield.

Table S 3: Photochemical Decarboxylation based on literature conditions.

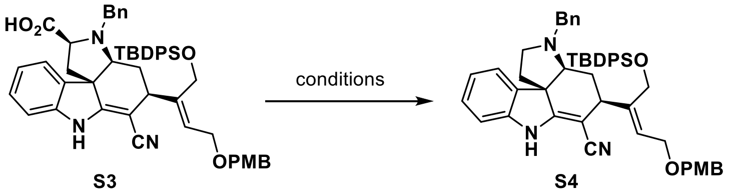

S3 S4

N#	Conditions	Result	Scale
1	[Me-Mes-Acr]BF_4, PhS-SPh, 2,6-Lutidine, DCE, 456 nm, 16 h[37]	traces	53 µmol
2	Cs_2CO_3, [Ir(dF(CF_3)ppy_2dtbbpy]PF_6, *t*BuSH, MeCN/H_2O, 456 nm, 2 h	14%	61 µmol
3	Fe(NO_3)$_3$, di(2-picolyl)amine, (TRIPS)$_2$, Na_2CO_3, DCE/H_2O, 390 nm, 20 h[38]	33%	61 µmol
4	PrPPT, K_3PO_4, MeCN/H_2O, 390 nm, 24 h[39]	decomposition	10 µmol
5	Phen, 1,4-Dicyanobenzene, *t*BuSH, MeCN/H_2O, UV-B, 4 h[40,41]	Traces, Bn deprotection	10 µmol
6	Cs_2CO_3, [Ir(dF(CF_3)ppy_2dtbbpy]PF_6, *t*BuSH, MeCN/H_2O, 456 nm, 4 h	54%	61 µmol
7	[Mes-(*t*Bu)$_2$Acr-Ph]BF_4, (PHS)$_2$, DIPEA, TFE, 456 nm, 18 h[42]	60%*	52 µmol
8	Mes-(*t*Bu)$_2$Acr, *p*ClC_4H_6SH, DCM, 390 nm[43]	42%	52 µmol
9	Cs_2CO_3[Ir(dF(CF_3)ppy_2dtbbpy]PF_6, *t*BuSH, DCM/H_2O, 456 nm, 24 h[44]	57%	50 µmol
10	Cs_2CO_3, [Ir(dF(CF_3)ppy_2dtbbpy]PF_6, *p*ClPhSH, DCM/H_2O, 456 nm, 24 h[44]	62%	50 µmol

11	Cs_2CO_3, [Ir(dF(CF_3)ppy_2dtbbpy]PF_6, *pClC₄H₆SH*, DCM/H_2O, 456 nm, 24 h[44]	88%	500 µmol
12	Cs_2CO_3, [Ir(dF(CF_3)ppy_2dtbbpy]PF_6, *pClC₄H₆SH*, DCM/H_2O, 456 nm, 24 h[44]	87%	1 mmol

* Based on NMR integrals of not fully purified product. Abbreviations: [Me-Mes-Acr]BF_4 : 9-Mesityl-10-methylacridinium tetrafluoroborate, $(TRIPS)_2$: Bis(2,4,6-triisopropylphenyl) disulfide, PrPTT: 2,4,6,8-Tetraoxo-1,3,7,9-tetrapropyl-1,2,3,4,6,7,8,9-octahydropyrimido[5,4-g]pteridine, Phen: Phenanthrene, [Mes-(*t*Bu)$_2$Acr-Ph]BF_4: 9-Mesityl-3,6-di-*tert*-butyl-10-phenylacridinium tetrafluoroborate, Mes-(*t*Bu)$_2$Acr: 9-Mesityl-3,6-di-tert-butyl acridine.

Table S 4: Photochemical Decarboxylation based on literature reported conditions.

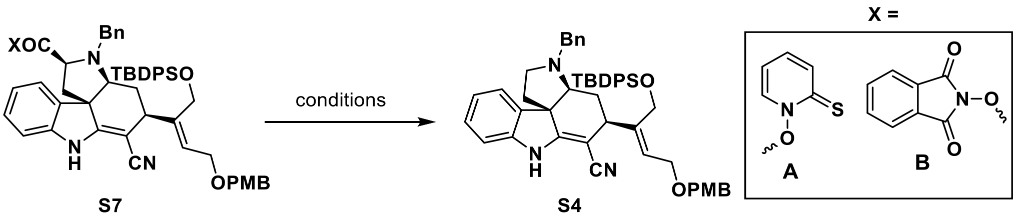

N#	Substrate	Conditions	Result	Scale
1	A*	*n*Bu_3SnH, AIBN, benzene, 80 °C, 2 h[45]	traces, decomposition	17 µmol
2	A*	Zn-tetraphenylporphyrin MeCN, *t*-dodecanethiol, red LED, 15 min[46]	19%	60 µmol
3	B	Ru$(bpy)_3Cl_2$, BNAH, *t*BuSH, MeCN/H_2O, 467 nm, 3 h[47]	traces	10 µmol
4	B	Mn, Hantzsch ester, DMF, 50 °C, 16 h[48]	n.d. (overreduced products)	10 µmol
5	B	EtOCSSK, γ-terpinene, DMSO, 456 nm, 24 h[49]	decomposition	10 µmol
6	B	fac-Ir$(ppy)_3$, Mg$(ClO_4)_2$, NMP, visible light, 24 h[50]	decomposition	10 µmol
7	B	$PhSiH_3$, Zn, $NiCl_2$, dtbbpy, THF/DMF/iPrOH, 40 °C, 24 h[51]	traces	10 µmol

* not isolated but subjected to decarboxylation conditions directly. n.d.: not detected. Abbreviations: BNAH: 1-Benzyl-1,4-dihydronicotinamide.

Table S 5: Optimization of TBDPS deprotection.

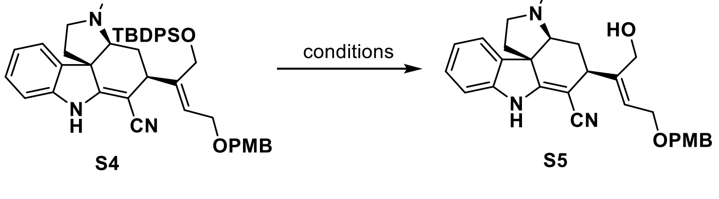

N#	Conditions (eq.)	Result	Scale
1	TBAF (3), THF, r.t 2 h	44%	0.13 mmol
2	TBAF (1), AcOH (1), DMF, 0 °C to rt	54%	0.03 mmol
3	HF-pyridine (2), THF, 0 °C to rt	0%, traces	0.1 mmol

4	HF-pyridine (10), pyr., r.t, 24 h	traces	0.1 mmol
5	TBAF (1), AcOH (3), DMF, 0 °C to rt, 6 h	61%	0.1 mmol
6	TBAF (1), AcOH (3), DMF, 0 °C zu rt, 3 h	81%	0.3 mmol

Debenzylation

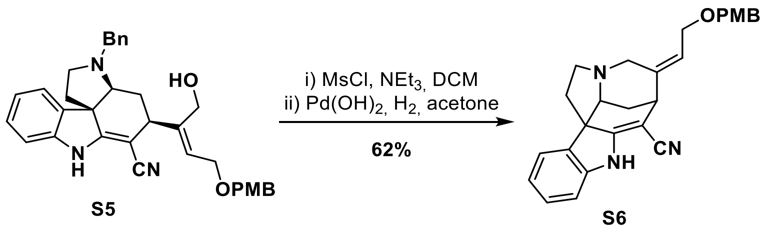

Schemes of unsuccessful attempts

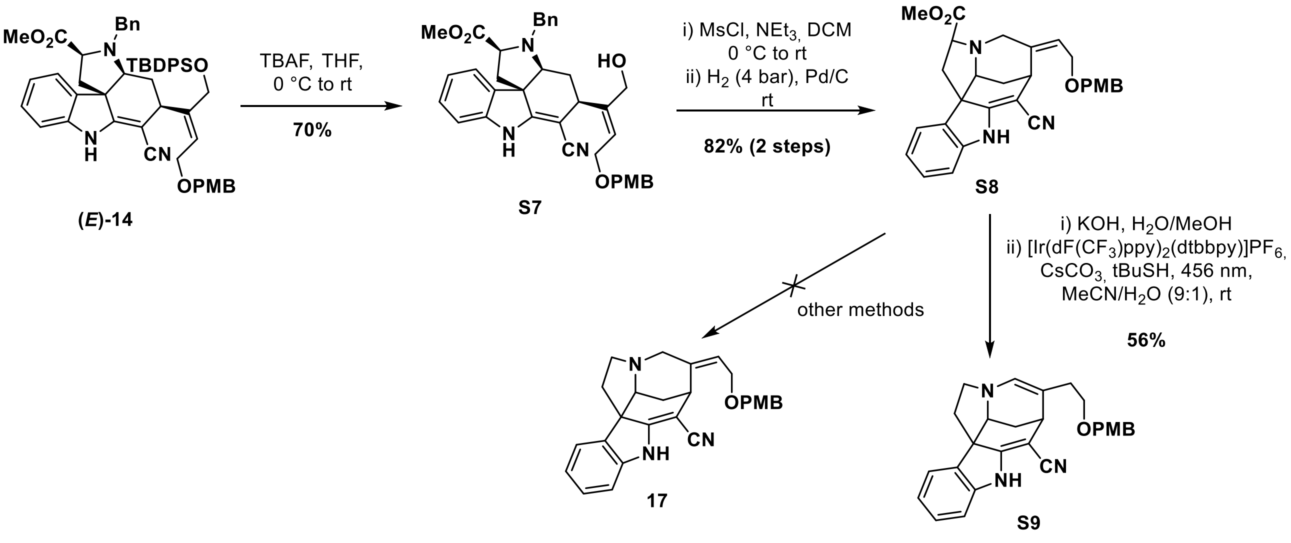

For closure of the D-ring and decarboxylation/debenzylation, different pathways were possible. In a first attempt, the D-ring was closed by deprotection of the silyl ether (*E*)-**14** and activation of the resulting alcohol **S7**. It was then possible to debenzylate the quaternary ammonium salt by hydrogenolysis. Decarboxylation attempts of this substrate failed under various utilized strategies.

Under iridium photocatalytic decarboxylation conditions, compound **S9** was isolated in 56% yield, which possesses a double bond shifted to the D-ring.

Attempts of upscaling the reaction towards **S8** failed and only the inner salt **S10** was obtained. This on the other hand could have allowed the decarboxylation under the previous conditions since quaternization of the nitrogen should limit the double bond isomerization. Unfortunately, all tested conditions failed to deliver the decarboxylated compound **17**. Furthermore, it was also not possible to remove the benzyl protecting group from this substrate.

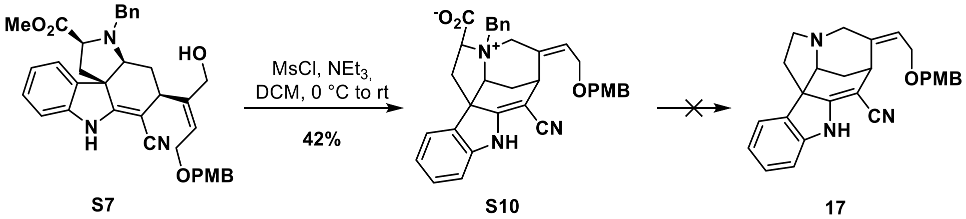

Endgame

Following the successful route towards compound **17**, conversion to the Wieland-Gumlich aldehyde was attempted. Previously, Qin et al.[30] had demonstrated a reduction of the enaminonitrile and subsequent Pinner reaction which cleaves the protecting group and converts the aldehyde to the methyl ester. This common intermediate was then converted to strychnine by a well-known sequence.

In our attempt to reduce the enaminonitrile, we were able to isolate the desired compound **S11** in 26% yield, as well as the fragmented product **S12** in 30% yield, greatly diminishing the overall efficiency. This type of stemmadenine-type fragmentation was observed in several occasions before.[7,52–55]

Treatment of compound **S11** with HCl in MeOH resulted only in PMB ether cleavage and at prolonged reaction times only decomposition was observed.

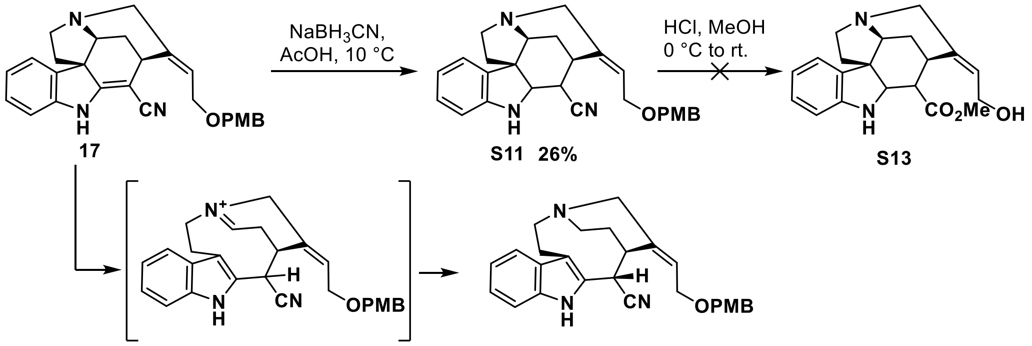

To limit the amount of fragmentation product **S12**, compound **S14** was employed. Quaternization of the nitrogen hinders the formation of fragmentation. Indeed, the reaction only produced only product **S15** in 30% yield. Due to tedious purification and no enhanced yield this attempt was not pursued further.

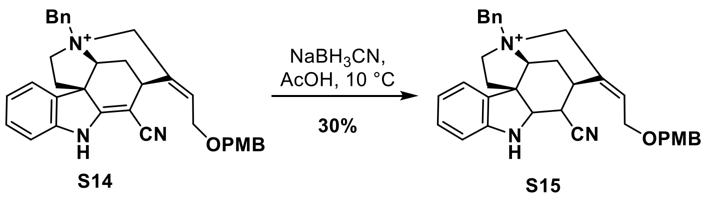

Procedures and Compound Characterizations

Synthesis of 10

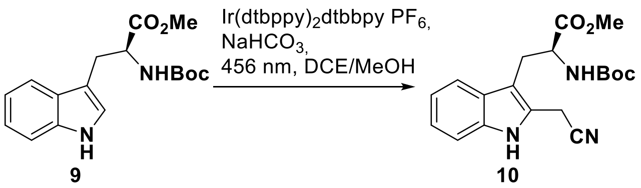

In a dried 20 mL microwave reaction vessel, Boc-Trp-OMe (3.18 g, 10.0 mmol, 1.0 eq.), $[Ir(dtbppy)_2(dtbbpy)]PF_6$ (11.4 mg, 10 µmol, 0.1 mol%) and $NaHCO_3$ (2.52 g, 30.0 mmol, 3.0 eq.) were dissolved in dry and degassed DCE/MeOH (1:1, 20 mL). Bromoacetonitrile (0.7 mL, 10 mmol, 1.0 eq.) was added and the mixture was irradiated with two Kessil LED-spotlights (456 nm, 50 W, 25% intensity) for 72 h. In this time, four additional portions of bromacetonitrile (4 x 0.35 mL, 5.0 mmol, 0.5 eq.) were added every 16 h. The reaction mixture was added to a sat. $NaHCO_3$ solution and was extracted three times with EtOAc. The combined organic extracts were dried over $MgSO_4$ and the solvent was removed. The crude was purified by column chromatography (silica, cHex/EtOAc 3:1) and the product was obtained as a colorless solid (2.64 g, 7.39 mmol, 74%).

$\boldsymbol{R_f}$ = 0.13 (cHex/EtOAc 3:1)

^{1}H-NMR, COSY (300 MHz, $CDCl_3$) δ = 8.47 (s, 1H, NH), 7.49 (d, *J* = 7.8 Hz, 1H, H-4_{Ind}), 7.37 – 7.28 (m, 1H, H-7_{Ind}), 7.20 (ddd, *J* = 8.2, 7.0, 1.3 Hz, 1H, H-6_{Ind}), 7.12 (ddd, *J* = 8.0, 7.1, 1.2 Hz, 1H, H-5_{Ind}), 5.18 (d, *J* = 8.0 Hz, 1H, CON*H*), 4.71 – 4.53 (m, 1H, C-α), 3.88 (s, 2H, CH_2CN), 3.65 (s, 3H, CO_2CH_3), 3.25 (d, *J* = 5.7 Hz, 2H, C-β), 1.44 (s, 9H, 3 x CH_3^{Boc}) ppm.

^{13}C-NMR, HSQC, HMBC (75 MHz, $CDCl_3$) δ 172.3 (*C*O_2), 155.1 (CO_2^{Boc}), 135.7 (C-$7a^{Ind}$), 128.1 (C-$3a^{Ind}$), 123.7 (C-2^{Ind}), 122.9 (C-6^{Ind}), 120.2 (C-5^{Ind}), 118.8 (C-4^{Ind}), 116.5 (*C*≡N), 111.1 (C-7^{Ind}), 108.8 (C-3^{Ind}), 80.2 (Cq^{Boc}), 54.1 (C-α), 52.6 (CO_2CH_3), 28.3 (3 C, CH_3^{Boc}), 27.1 (C-β), 15.6 (*C*H_2CN) ppm.

IR (ATR): $\tilde{\nu}$ [cm^{-1}] = 3358, 2978, 2253, 1736, 1694, 1502, 1367, 1281, 1165, 734.

ESI-MS (+ / *m/z* in %): 380.2 (100) [M + Na]+, 258.1 (76) $[M – Boc +H]^+$.

HRMS (ESI): found 380.1574 $[M + Na]^+$ (calc. for $[C_{19}H_{23}N_3NaO_4]^+$: 380.1581).

Melting range: 62.6-64-1 °C (DCM).

$[\alpha]_D^{21}$ = –3.9 (c=1.0 in MeOH)

Synthesis of 4

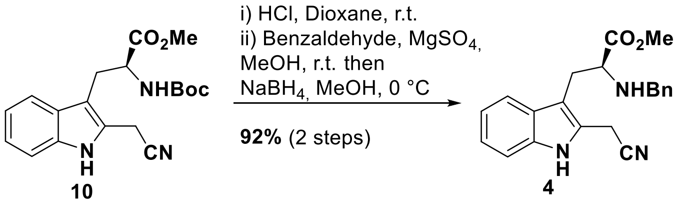

To compound **10** (1.64 g, 4.59 mmol, 1.0 eq.) was added a solution of HCl in dry 1,4-dioxane (4 M, 35 mL). The reaction mixture was stirred for 1 h at rt. The precipitated hydrochloride was filtered off and washed with small portions of dioxane. The solid was suspended in a $NaHCO_3$ solution (50% aq., 100 mL) and stirred for 10 min at rt. and extracted three times with EtOAc. The combined organics were washed with brine and dried over Na_2SO_4 and the solvent was removed under reduced pressure.

The residue was dissolved in dry MeOH (15 mL) and $MgSO_4$ (1.10 g, 9.18 mmol, 2.0 eq.) and benzaldehyde (510 µL, 5.05 mmol, 1.1 eq.) were added. The suspension was stirred for 20 h at rt and was cooled to 0 °C before $NaBH_4$ (261 mg, 6.9 mmol, 1.5 eq.) was added in small portions. The solution was stirred for 1 h at 0 °C and was quenched by addition of a $NaHCO_3$ solution. The mixture was extracted three times with EtOAc and the combined organics were washed with brine, dried over Na_2SO_4 and the solvent was removed under reduced pressure. The crude was purified by column chromatography (silica, cHex/EtOAc 2:1) and the product was obtained as a yellow oil (1.46 g, 4.20 mmol, 92%).

R_f = 0.21 (cHex/EtOAc 2:1) [UV]

^{1}H-NMR, COSY (400 MHz, $CDCl_3$) δ 8.29 (s, 1H, N*H*), 7.47 (dd, *J* = 7.9, 1.1 Hz, 1H, H-4^{Ind}), 7.33 (d, *J* = 8.1 Hz, 1H, H-7^{Ind}), 7.29 – 7.21 (m, 5H, H-2^{Bn}-H-6^{Bn}), 7.21 – 7.18 (m, 1H, H-6^{Ind}), 7.11 (ddd, *J* = 8.0, 7.1, 1.1 Hz, 1H, H-5^{Ind}), 4.06 – 3.87 (m, 2H, C*H*2CN), 3.81 (d, *J* = 13.1 Hz, 1H, C*H*'$_2$Ph), 3.65 (d, *J* = 13.0 Hz, 1H, C*H*''$_2$Ph), 3.63 (s, 3H, CO_2CH_3), 3.59 (t, *J* = 6.5 Hz, 1H, C*H*-α), 3.17 – 3.02 (m, 2H, CH_2-β) ppm.

^{13}C-NMR, HSQC, HMBC (101 MHz, $CDCl_3$) δ 175.1 (CO_2), 139.3 (C-1'^{Ph}), 135.7 (C-7a^{Ind}), 128.4 (2C, C-3'^{Ph}, C-5'^{Ph}), 128.1 (2C, C-2'^{Ph}, C-6'^{Ph}), 127.9 (C-3a^{Ind}), 127.2 (C-4'^{Ph}), 123.6 (C-2^{Ind}), 122.8 (C-6^{Ind}), 120.1 (C-5^{Ind}), 118.7 (C-4^{Ind}), 116.7 (*C*≡N), 111.0 (C-7^{Ind}), 109.8 (C-3^{Ind}), 60.9 (C-α), 52.3 (CH_2Ph), 52.0 (CO_2CH_3), 28.4 (C-β), 15.9 (CH_2CN) ppm.

IR (ATR): $\tilde{\nu}$ [cm^{-1}] = 3374, 2950, 2252, 1727, 1456, 1199, 1173, 910, 734, 699.

ESI-MS (+ / *m/z* in %): 348.2 (100) $[M+H]^+$.

HRMS (ESI): found 370.1521 $[M + Na]^+$ (calc. for $[C_{21}H_{21}N_3NaO_2]^+$: 370.1526).

$[\alpha]_D^{21}$ = −4.7 (c=0.57 in MeOH)

Synthesis of 12

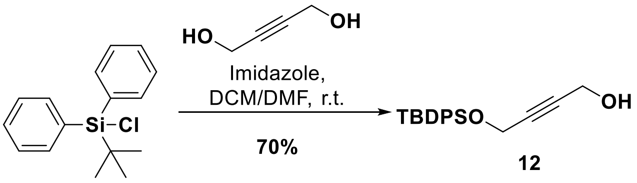

2-Butyne-1,4-diol (7.32 g, 85.0 mmol, 2.0 eq.) and imidazole (3.47 g, 51.0 mmol, 1.2 eq.) were dissolved in dry DCM/DMF (12:1, 300 mL). In one portion TBDPSCl (10.9 mL, 42.5 mmol, 1.0 eq.) was added and the mixture was stirred for 3 h at rt. The reaction mixture was diluted with Et_2O (500 mL) and washed with H_2O and brine. The organic phase was dried over Na_2SO_4 and the solvent was removed under reduced pressure. The residue was purified by column chromatography (silica, cHex/EtOAc 10:1 to 2:1). The product was obtained as a colorless oil (9.63 g, 29.7 mmol, 70%).

R R_f = 0.18 (cHex/EtOAc 10:1).

^{1}H-NMR, COSY (300 MHz, $CDCl_3$) δ [ppm] = 7.78 – 7.67 (m, 4H, H_{ortho}), 7.51 – 7.34 (m, 6H, $H_{meta,para}$), 4.37 (t, *J* = 1.8 Hz, 2H, *H*-4), 4.20 (dt, *J* = 6.1, 1.8 Hz, 2H, *H*-1), 1.43 (t, *J* = 6.1 Hz, 1H, O*H*), 1.07 (s, 9H, $C_q(CH_3)_3$) ppm.

^{13}C-NMR, HSQC, HMBC (75 MHz, $CDCl_3$) δ [ppm] = 135.7 (C_{ortho}), 133.1 (C_{ipso}), 129.8 (C_{para}), 127.7 (C_{meta}), 84.2 (*C*-2), 83.5 (*C*-3), 52.6 (*C*-4), 51.2 (*C*-1), 26.7 ($C_q(CH_3)_3$), 19.1 ($C_q(CH_3)_3$) ppm.

IR (ATR): $\tilde{\nu}$ [cm^{-1}] = 3361, 3071, 3958, 2931, 2858, 1472, 1427, 1372, 1134, 1111, 1074, 1010, 823, 721, 739, 702, 613, 505.

ESI-MS (+ / *m/z* in %): 247.1 (100) $[M + Na]^+$.

Analytical data are in accordance with the literature.[56,57]

Synthesis of 13

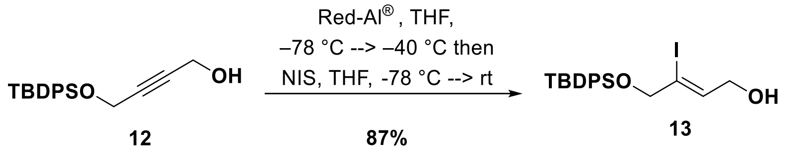

To a solution of **12** (9.00 g, 27.8 mmol, 1.0 eq.) in dry THF (140 mL) at −78 °C was added Red-Al® (60% in toluene, 15.4 mL, 47.2 mmol, 1.7 eq.) added dropwise. The solution was warmed to −40 °C and stirred at that temperature for 3 h. The solution was cooled to −78 °C and NIS (11.3 g, 50.0 mmol, 1.8 eq.) in dry THF (25 mL) was added slowly. The reaction mixture was stirred at −78 °C for 30 min and afterwards for 1 h at rt. The mixture was poured into a solution of $Na_2S_2O_3$/K-Na-tartrate (1:1, sat., 400 mL) and was extracted three times with Et_2O. The organics were dried over Na_2SO_4 and the solvent was removed under reduced pressure. The residue was purified by column chromatography (silica, cHex/EtOAc 3:1) and the product was obtained as a colorless oil (10.9 g, 24.2 mmol, 87%).

R_f = 0.47 (cHex/EtOAc 3:1).

^{1}H-NMR, COSY (300 MHz, $CDCl_3$) δ [ppm] = 7.73 – 7.63 (m, 4H, *H*-2,6^{TBDPS}), 7.52 – 7.34 (m, 6H, *H*-3,4,5^{TBDPS}), 6.32 (tt, *J* = 6.3, 1.8 Hz, 1H, *H*-2), 4.30 – 4.24 (m, 4H, *H*-1, *H*-4), 1.55 (t, *J* = 5.9 Hz, 1H, O*H*), 1.10 (s, 9H, 3xCH_3^{tBu}) ppm.

^{13}C-NMR, HSQC, HMBC (75 MHz, $CDCl_3$) δ [ppm] = 135.5 (4C, *C*-2,6^{TBDPS}), 133.0 (2C, *C*-1^{TBDPS}), 132.4 (*C*-2), 129.9 (2C, *C*-4^{TBDPS}), 127.8 (4C, *C*-3,5^{TBDPS}), 107.2 (*C*-3), 71.4 (*C*-4), 66.5 (*C*-1), 26.8 (3C, 3x*C*H_3^{tBu}), 19.3 (C_q^{tBu}) ppm.

IR (ATR): $\tilde{\nu}$ [cm^{-1}] = 3327, 2931, 2857, 1427, 1112, 1050, 1007, 933, 823, 701.

ESI-MS (+ / *m/z* in %): 475.0 (48) [M+Na]$^+$, 435.1 (37) [M-OH]$^+$.

The analytical data is in accordance with the literature.[58]

Synthesis of **6**

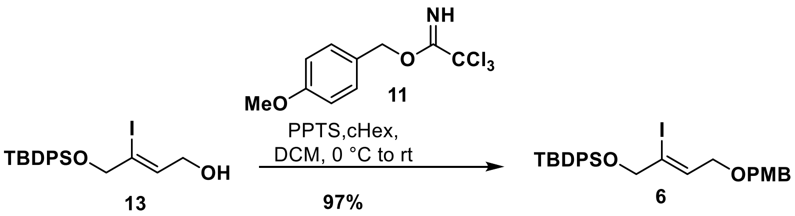

To a solution of **13** (3.00 g, 6.63 mmol, 1.0 eq.) and PPTS (167 mg, 0.663 mmol, 0.1 eq.) in cHex (26 mL) and DCM (13 mL) at 0 °C was added dropwise **11** (2.81 g, 9.95 mmol, 1.5 eq.). The reaction mixture was warmed to rt. and stirred for 18 h. The reaction was quenched with aqueous $NaHCO_3$ solution and the mixture was extracted three times with DCM. The combined organics were dried over $MgSO_4$ and the solvent was removed under reduced pressure. The residue was purified by column chromatography (silica, cHex/EtOAc 50:1) and the product was obtained as a colorless oil (3.68 g, 6.43 mmol, 97%).

R_f = 0.23 (^{c}Hex/EtOAc 50:1).

^{1}H-NMR, COSY (300 MHz, $CDCl_3$) δ [ppm] = 7.73 – 7.62 (m, 4H, *H*-2^{TBDPS}, *H*-6^{TBDPS}), 7.48 – 7.35 (m, 6H, *H*-3,4,5^{TBDPS}), 7.32 – 7.26 (m, 2H, *H*-2^{PMB}, *H*-6^{PMB}), 6.95 – 6.81 (m, 2H, *H*-3^{PMB}, *H*-5^{PMB}), 6.34 (tt, *J* = 5.7, 1.7 Hz, 1H, *H*-3), 4.46 (s, 2H, CH_2^{PMB}), 4.28 (q, *J* = 1.6 Hz, 2H, H_2-1), 4.15 (dt, *J* = 5.7, 1.5 Hz, 2H, H_2-4), 3.81 (s, 3H, CH_3^{PMB}), 1.09 (s, 9H, 3xCH_3) ppm.

^{13}C-NMR, HSQC, HMBC (75 MHz, $CDCl_3$) δ [ppm] = 159.3 (*C*-4^{PMB}), 135.5 (4C, *C*-2,6^{TBDPS}), 133.0 (2C, *C*-1^{TBDPS}), 130.8 (*C*-3), 130.1 (*C*-1^{PMB}), 129.9 (2C, *C*-4^{TBDPS}), 129.5 (2C, *C*-2,6^{PMB}), 127.8 (4C, *C*-3,5^{TBDPS}), 113.8 (2C, *C*-3,5^{PMB}), 107.4 (*C*-2), 73.4 (*C*-4), 72.1 (*C*H_2^{PMB}), 71.5 (*C*-1), 55.3 (*C*H_3^{PMB}), 26.8 (3C, 3xCH_3^{tBu}), 19.3 (C_q^{tBu}) ppm.

IR (ATR): $\tilde{\nu}$ [cm^{-1}] = 2895, 2856, 1613, 1513, 1248, 1112, 1039, 822, 702, 504.

ESI-MS (+ / *m/z* in %): 435.1 (47) [M-OPMB]$^+$, 595,2 (23) [M+Na]$^+$

HRMS (ESI): found 595.1123 [M + Na]$^+$ (calc for $[C_{28}H_{33}INaO_3Si]^+$: 595.1136).

Synthesis of compound **5**

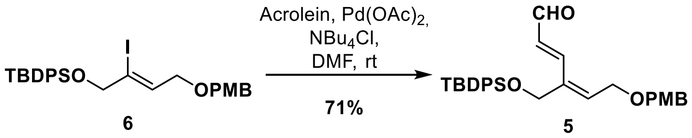

Based on a method of Jeffery.[59]

To a solution of **6** (2.2.4 g, 3.91 mmol, 1.0 eq.), Bu_4NCl (1.09 g, 3.91 mmol, 1.0 eq.) and $NaHCO_3$ (656 mg, 7.82 mmol, 2.0 eq.) in dry DMF (40 mL) was added $Pd(OAc)_2$ (34 mg, 0.15 mmol, 4 mol%) and freshly distilled acrolein (0.9 mL, 19.3 mmol, 4.9 eq.). The reaction mixture was stirred for 48 h at rt. Water (100 mL) was added and the mixture was extracted four times with Et_2O. The combined organics were dried over $MgSO_4$ and the solvent was removed under reduced pressure. The residue was purified by column chromatography (silica, cHex/EtOAc 8:1) and the product was obtained as a yellowish oil (1.39 g, 2.78 mmol, 71%).

$\boldsymbol{R_f}$ = 0.26 [CyH/EE 8:1 [UV])

^{1}H-NMR, COSY (300 MHz, $CDCl_3$) δ 9.53 (d, *J* = 7.7 Hz, 1H, C*H*O), 7.68 (dt, *J* = 6.4, 1.7 Hz, 4H, H-2^{Ph}, H-6^{Ph}), 7.50 – 7.35 (m, 7H, H-3, H-3^{Ph}H-4^{Ph}, H-5^{Ph}), 7.33 – 7.28 (m, 2H, H-2^{PMB}, H-6^{PMB}), 7.04 – 6.83 (m, 2H, H-3^{PMB}, H-5^{PMB}), 6.36 (t, *J* = 6.6 Hz, 1H, H-5), 6.13 (dd, *J* = 16.0, 7.7 Hz, 1H, H-2), 4.51 (s, 2H, C*H*$_2$-^{PMB}), 4.37 (d, *J* = 1.4 Hz, 2H, H$_2$-1'), 4.31 (dd, *J* = 6.6, 1.3 Hz, 2H, H$_2$-6), 3.84 (s, 3H, OC*H*$_3$), 1.09 (s, 9H, C(C*H*$_3$)$_3$) ppm.

^{13}C-NMR, HSQC, HMBC (75 MHz, $CDCl_3$) δ 194.2 (*C*HO), 159.4 (C-4^{PMB}), 145.6 (C-3), 136.4 (C-4), 135.5 (4C, C-2^{Ph}, C-6^{Ph}), 134.6 (C-5), 133.0 (2C, C-1^{Ph}), 129.9 (2C, C-4^{Ph}), 129.7 (C-1^{PMB}), 129.6 (2C, C-2^{PMB}, C-6^{PMB}), 129.2 (C-2), 127.8 (4C, C-3^{Ph}, C-5^{Ph}), 113.9 (2C, C-3^{PMB}, C-5^{PMB}), 72.2 (C*H*$_2$-^{PMB}), 65.2 (C-6), 63.7 (C-1'), 55.3 (O*C*H$_3$), 26.8 (3C, C(*C*H$_3$)$_3$), 19.3 (*C*(CH$_3$)$_3$) ppm.

IR (ATR): $\tilde{\nu}$ [cm^{-1}] = 2955, 2932, 2857, 1683, 1612, 1513, 1463, 1428, 1249, 1174.

ESI-MS (+ / *m/z* in %): 523.3 (100) $[M+H]^+$

HRMS (ESI): found 523.2273 $[M + Na]^+$ (calc for $[C_{31}H_{36}O_4SiNa]^+$: 523.2275).

<u>Characterization of S1</u>

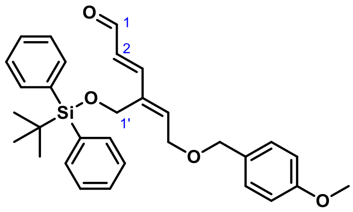

$\boldsymbol{R_f}$ = 0.13 (cHex/EtOAc 8:1).

^{1}H-NMR, COSY (600 MHz, $CDCl_3$): δ = 9.56 (d, 1H, *J* = 7.8 Hz, CHO), 7.65–7.61 (m, 4H, 2 x H-2,6^{TBDPS}), 7.47–7.42 (m, 2H, 2 x H-4^{TBDPS}), 7.41–7.36 (m, 4H, 2 x H-3,5^{TBDPS}), 7.19–7.15 (m, 2H, H-2,6^{PMB}), 7.01 (d, 1H, *J* = 15.8 Hz, H-3), 6.87–6.83 (m, 2H, H-3,5^{PMB}), 6.33 (dd, 1H, *J* = 15.8 Hz, 7.8 Hz, H-2), 6.14 (t, J = 6.0

Hz, H-5), 4.32 (s, 2H, H_2-1'), 4.29 (s, 2H, CH_2^{PMB}), 3.92 (d, J = 6.0 Hz, H_2-6), 3.80 (s, 3H, OCH_3), 1.01 (s, 9H, $C_q(CH_3)_3$) ppm.

^{13}C-NMR, HSQC, HMBC (151 MHz, CDCl3): δ = 194.5 (CHO), 159.5 (C-4^{PMB}), 154.0 (C-3), 140.7 (C-5), 137.3 (C-4), 135.8 (4C, 2 x C-2,6^{TBDPS}), 133.0 (2C, 2 x C-1^{TBDPS}), 130.1 (2C, 2 x C-4^{TBDPS}), 129.8 (C-1^{PMB}), 129.6 (2C, C-2,6^{PMB}), 129.5 (C-2), 128.0 (4C, 2 x C-3,5^{TBDPS}), 114.0 (2C, C-3,5^{PMB}), 72.6 (CH_2^{PMB}), 66.4 (C-6), 59.1 (H_2-1'), 55.4 (OCH_3), 26.9 (3C, $C_q(CH_3)_3$), 19.3 (C_q(CH3)3) ppm.

IR (ATR): $\tilde{\nu}$ [cm^{-1}] = 3072, 2999, 2857, 1680, 1611, 1513, 1248, 1111, 823, 704.

ESI-MS (+ / *m/z* in %): 523.2 (100) $[M+Na]^+$

HRMS (ESI): found 523.2260 $[M + Na]^+$, (calc for $[C_{31}H_{36}NaO_4Si]^+$: 523.2275).

Synthesis of **14**

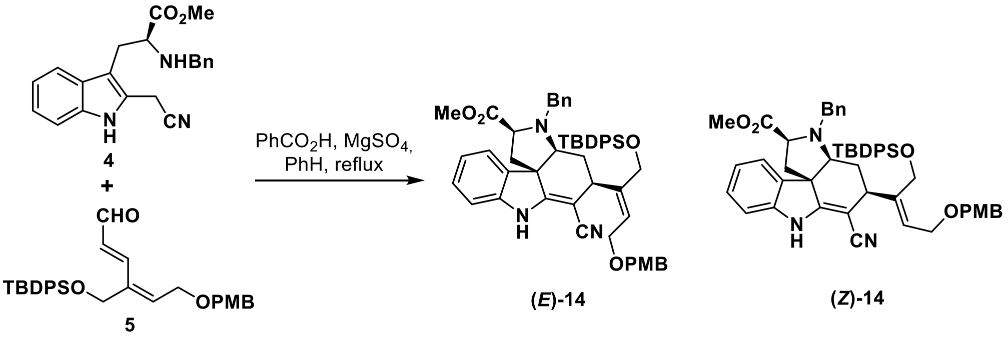

Based on a method by Kuehne.[11,60]

In a three-necked flask equipped with a Dean-Stark trap, a mixture of indole **4** (9.94 g, 28.6 mmol, 1.0 eq.), $PhCO_2H$ (3.49 g, 28.6 mmol, 1.0 eq.) and $MgSO_4$ (6.88 g, 57.2 mmol, 2.0 eq.) in dry benzene (200 mL) were refluxed under argon. Over 4 h, a solution of aldehyde (**5**, 15.8 g, 31.6 mmol, 1.1 eq.) in dry benzene (50 mL) was added dropwise to the refluxing solution. After the addition was completed the reaction mixture was refluxed for 14 h and afterwards quenched by addition of a aq. $NaHCO_3$ solution. The phases were separated and the aqueous layer was extracted twice with EtOAc. The combined organic layers were washed with H_2O and brine, dried over $MgSO_4$ and the solvent was removed under reduced pressure. The residue was purified by column chromatography (silica, cHex/EtOAc 6:1) to give (*E*)-**14** (11.5 g, 13.9 mmol, 49%) and (*Z*)-**15** (3.21 g, 3.87 mmol, 14%) as slight yellow solids.

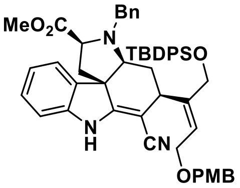

(*E*)-14

R_f = 0.47 (cHex/EtOAc 3:1) [Vanillin]

^{1}H-NMR, COSY (600 MHz, CD_3CN) δ 7.73 (s, 1H, H-7), 7.70 (app. td, *J* = 8.2, 1.5 Hz, 4H, H-2,6 ^{TBDPS}, H-2,6$^{TBDPS'}$), 7.48 – 7.43 (m, 2H, H-4 ^{TBDPS}, H-4 $^{TBDPS'}$), 7.42 (m, 4H, H-3,5 ^{TBDPS}, H-3,5 $^{TBDPS'}$), 7.39 – 7.36 (m, 2H, H-2,6^{Bn}), 7.34 – 7.29 (m, 2H, H-3,5^{Bn}), 7.28 (d, *J* = 7.5 Hz, 1H, H-11), 7.27 – 7.24 (m, 1H, H-4^{Bn}), 7.22

(d, *J* = 6.6 Hz, 2H, H-2,6^{PMB}), 7.16 (td, *J* = 7.7, 1.2 Hz, 1H, H-9), 6.89 (td, *J* = 7.5, 1.0 Hz, 1H, H-10), 6.85 (d, *J* = 7.3 Hz, 1H, H-8), 6.85 – 6.82 (m, 2H, H-3,5^{PMB}), 5.94 (tt, *J* = 6.6, 1.6 Hz, 1H, H-3'), 4.42 (d, *J* = 11.5 Hz, 1H, H_a-CH_2^{PMB}), 4.37 (d, *J* = 11.5 Hz, 1H, H_b-CH_2^{PMB}), 4.27 (d, *J* = 15.2 Hz, 1H, H_a-1'), 4.24 (d, *J* = 14.1 Hz, 1H, H_b-1'), 4.17 – 4.11 (m, 1H, H_a-4'), 4.08 (ddt, *J* = 12.4, 6.5, 1.3 Hz, 1H, H_b-4'), 4.05 (s, 2H, CH_2^{Bn}), 3.88 (dd, *J* = 9.8, 7.2 Hz, 1H, H-2), 3.70 (s, 3H, OCH_3^{PMB}), 3.47 (s, 3H, CO_2CH_3), 3.44 (dd, *J* = 10.2, 6.9 Hz, 1H, H-5), 3.27 (dd, *J* = 9.6, 6.2 Hz, 1H, H-3a), 2.23 (dd, *J* = 12.5, 9.8 Hz, 1H, H_a-1), 2.01 – 1.95 (m, 1H, CH_2-4[partially behind solvent signal]), 1.88 (dd, *J* = 12.4, 7.2 Hz, 1H, H_b-1), 1.06 (s, 9H, $C(CH_3)_3^{TBDPS}$) ppm.

^{13}C-NMR, HSQC, HMBC (151 MHz, CD_3CN) δ 173.0 (CO_2Me), 162.2 (C-6a), 158.3 (C-4^{PMB}), 142.8 (C-7a), 140.5 (C-2'), 138.4 (C-1^{Bn}), 134.8 (C-11a), 134.6 (4C, C-2,6^{TBDPS}, C-2,6$^{TBDPS'}$), 132.5 (C-1^{TBDPS}), 132.5 (C-1$^{TBDPS'}$), 129.8 (C-1^{PMB}), 129.0 (2C, C-4^{TBDPS}, C-4$^{TBDPS'}$), 128.6 (2C, C-2,6^{PMB}), 127.9 (2C, C-2,6^{Bn}), 127.4 (3C, C-3,5^{Bn}, C-9), 127.0 (4C, C-3,5^{TBDPS}, C-3,5$^{TBDPS'}$), 126.3 (C-4^{Bn}), 123.4 (C-3'), 121.8 (C-11), 120.0 (C-10), 117.1 (*C*N), 112.8 (2C, C-3,5^{PMB}), 108.7 (C-8), 77.2 (C-6), 70.5 (CH_2^{PMB}), 64.0 (C-4'), 63.9 (C-1'), 61.6 (C-3a), 61.2 (C-2), 53.9 (OCH_3^{PMB}), 52.9 (CH_2^{Bn}), 52.4 (C-11b), 50.6 (CO_2CH_3), 44.3 (C-1), 35.4 (C-5), 31.3 (C-4), 25.4 (3C, $C(CH_3)_3$), 18.0 ($C(CH_3)_3$) ppm.

IR (ATR): $\tilde{\nu}$ [cm^{-1}] = 2951, 2930, 2856, 2194, 1735, 1644, 1613, 1513, 1465, 1359.

HRMS (ESI): found 830.3978, calc for $[C_{52}H_{56}N_3O_5Si]^+$: 830.3984.

Melting range: 61.3–62.8 °C [cHex/EtOAc]

$[\alpha]_D^{21} = -129.0$ (c = 0.1) [MeOH].

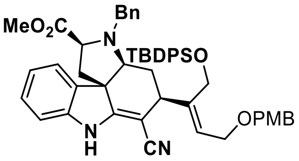

(*Z*)-14

R_f = 0.42 (cHex/EtOAc 3:1) [Vanillin]

^{1}H-NMR, COSY (600 MHz, CD_3CN) δ 8.10 (s, 1H, NH), 7.78 – 7.73 (m, 2H, H-2,6$^{TBDPS'}$), 7.72 – 7.66 (m, 2H, H-2,6^{TBDPS}), 7.51 – 7.40 (m, 6H, H-3,4,5^{TBDPS}, H-3,4,5$^{TBDPS'}$), 7.28 (ddt, *J* = 5.6, 3.9, 2.1 Hz, 3H, H-3,4,5^{Bn}), 7.25 – 7.20 (m, 2H, H-2,6^{Bn}), 7.18 – 7.14 (m, 2H, H-2,6^{PMB}), 7.12 (td, *J* = 7.7, 1.2 Hz, 1H, H-9), 6.84 (d, *J* = 7.4 Hz, 1H, H-8), 6.84 – 6.80 (m, 2H, H-3,5^{PMB}), 6.72 (td, *J* = 7.5, 1.0 Hz, 1H, H-10), 6.45 (d, *J* = 7.4 Hz, 1H, H-11), 5.47 (tdt, *J* = 7.0, 5.2, 1.9 Hz, 1H, H-3'), 4.50 (d, *J* = 12.5 Hz, 1H, H_a-1'), 4.46 (d, *J* = 12.5 Hz, 1H, H_b-1'), 4.28 (d, *J* = 11.5 Hz, 1H, H_a-CH_2^{PMB}), 4.23 (d, *J* = 11.5 Hz, 1H, H_b-CH_2^{PMB}), 3.86 (ddd, *J* = 12.6, 7.0, 1.8 Hz, 1H, H_a-4'), 3.79 (d, *J* = 13.5 Hz, 1H, H_a-CH_2^{Bn}), 3.75 (s, 3H, OCH_3^{PMB}), 3.76 – 3.72 (m, 1H, H_b-4'), 3.73 (d, *J* = 13.6 Hz, 1H, H_b-CH_2^{Bn}), 3.63 (dt, *J* = 3.7, 2.0 Hz, 1H, H-5), 3.57 (dd, *J* = 11.5, 5.6 Hz, 1H, H-2), 3.47 (dd, *J* = 5.7, 1.8 Hz, 1H, H-3a), 3.32 (s, 3H, CO_2CH_3), 2.35 (t, *J* = 11.6 Hz, 1H, H_a-1), 2.30 (ddd, *J* = 14.6, 3.1, 1.9 Hz, 1H, H_a-4), 1.58 (dd, *J* = 11.7, 5.6 Hz, 1H, H_b-1), 1.49 (dt, *J* = 14.7, 5.8 Hz, 1H, H_b-4), 1.05 (s, 9H, $C(CH_3)_3$) ppm.

^{13}C-NMR, HSQC, HMBC (151 MHz, CD_3CN) δ 173.3 (CO_2), 166.3 (C-6a), 160.1 (C-4^{PMB}), 143.9 (C-7a), 143.1 (C-2'), 137.9 (C-11a), 136.9 (C-1^{Bn}), 136.6 (2C, C-2,6$^{TBDPS'}$), 136.5 (2C, C-2,6^{TBDPS}), 134.4 (C-1^{TBDPS}), 134.4 (C-1$^{TBDPS'}$), 131.6 (C-1^{PMB}), 131.2 (2C, C-2,6^{Bn}), 130.9 (C-4^{TBDPS}), 130.9 (C-4$^{TBDPS'}$), 130.3 (2C, C-2, 6^{PMB}), 129.1 (2C, C-3,5^{Bn}), 129.1 (C-9), 128.9 (2C, C-3,5$^{TPBDS'}$), 128.8 (2C, C-3,5^{TBDPS}), 128.4 (C-4^{Bn}), 125.9 (C-3'), 122.5 (C-11), 121.6 (C-10), 120.2 (*C*N), 114.5 (2C, C-3,5^{PMB}), 110.4 (C-8), 77.1 (C-6), 72.6 (CH_2^{PMB}),

67.0 (C-4’), 65.6 (C-3a), 63.5 (C-2), 61.6 (C-1’), 56.2 (CH_2^{Bn}), 55.8 (OCH_3^{PMB}), 54.4 (C-11b), 52.2 (CO_2CH_3), 46.8 (C-1), 41.0 (C-5), 31.3 (C-4), 27.3 (3C, $C(CH_3)_3$), 19.8 ($C(CH_3)_3$) ppm.

IR (ATR): $\tilde{\nu}$ [cm^{-1}] = 3277, 2951, 2931, 2856, 2191, 1746, 1643, 1614, 1513, 1466.

ESI-MS (+ / *m/z* in %): 574.3 (100) [M−OTBDPS]$^+$.

HRMS (ESI): found 852.3780, calc for $[C_{52}H_{55}N_3NaO_5Si]^+$: 852.3803.

Melting range: 70.0–73.3 °C [cHex/EtOAc]

$[\alpha]_D^{21}$ = −73.3 (c = 0.1) [MeOH]

Synthesis of 15

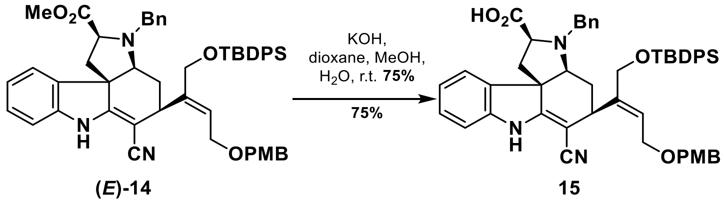

To a solution of (**E**)**-14** (955 mg, 1.15 mmol, 1.0 eq.) in dioxane/MeOH (1:1, 10 mL) was added KOH (10wt% in H_2O, 1.6 mL, 2.9 mmol, 2.5 eq.) dropwise. The solution was stirred at rt for 18 h. The reaction mixture was acidified with 1 M HCl to pH=2 and extracted three times with EtOAc. The combined organic layers were dried over $MgSO_4$ and the solvent was removed under reduced pressure. The residue was purified by column chromatography (silica, cHex/acetone 3:1) and the product was obtained as a yellow foam (705 mg, 0.865 mmol) in a yield of 75%.

R_f = 0.27 (cHex/acetone 3:1) [Seebach]

^{1}H-NMR, COSY (600 MHz, $CDCl_3$) δ 7.69 – 7.65 (m, 4H, H-2,6^{TBDPS}, H-2,6$^{TBDPS'}$), 7.43 – 7.40 (m, 2H, H-4^{TBDPS}, H-4$^{TBDPS'}$), 7.40 – 7.32 (m, 7H, H-3,5^{TBDPS}, H-3,5$^{TBDPS'}$, H-3,4,5^{Bn}), 7.30 (dd, *J* = 8.0, 1.4 Hz, 2H, H-2,6^{Bn}), 7.24 – 7.18 (m, 3H, H-2,6^{PMB}, H-9), 7.00 (d, *J* = 7.5 Hz, 1H, H-11), 6.90 (td, *J* = 7.5, 1.0 Hz, 1H, H-10), 6.85 – 6.79 (m, 3H, H-3,5^{PMB}, H-8), 6.71 (s, 1H, H-7), 5.96 (t, *J* = 6.5 Hz, 1H, H-3‘), 4.39 (s, 2H, CH_2^{PMB}), 4.18 (dd, *J* = 13.7, 1.7 Hz, 1H, H_a-1‘), 4.10 (d, *J* = 12.8 Hz, 1H, H_b-1‘), 4.09 – 4.05 (m, 3H, Ha-CH_2^{Bn}, CH_2-4‘), 3.99 (d, *J* = 13.0 Hz, 1H, H_b-CH_2^{Bn}), 3.94 (dd, *J* = 11.1, 6.7 Hz, 1H, H-2), 3.75 (s, 3H, CH_3^{PMB}), 3.40 (dd, *J* = 9.9, 6.2 Hz, 1H, H-5), 3.36 (dd, *J* = 9.5, 6.0 Hz, 1H, H-3a), 2.19 (dd, *J* = 12.6, 11.1 Hz, 1H, H_a-1), 2.08 (dd, *J* = 12.6, 6.7 Hz, 1H, H_b-1), 1.86 – 1.73 (m, 2H, H_2-4), 1.05 (s, 9H, $C(CH_3)_3^{TBDPS}$) ppm.

^{13}C-NMR, HSQC, HMBC (151 MHz, $CDCl_3$) δ 172.9 (CO), 162.4 (C-6a), 159.2 (C-4^{PMB}), 143.0 (C-7a), 140.3 (C-2‘), 136.5 (C-1^{Bn}), 135.6 (2C, C-2,6^{TBDPS}), 135.6 (2C, C-2,6$^{TBDPS'}$), 134.4 (C-11a), 133.1 (C-1^{TBDPS}), 133.0 (C-1$^{TBDPS'}$), 130.1 (C-1^{PMB}), 129.8 (C-4^{TBDPS}), 129.8 (C-4$^{TBDPS'}$), 129.6 (2C, C-2,6^{PMB}), 129.2 (2C, C-2,6^{Bn}), 129.1 (C-9), 129.0 (2C, C-3,5^{Bn}), 128.3 (C-4^{Bn}), 127.8 (2C, C-3,5^{TBDPS}), 127.8 (2C, C-3,5$^{TBDPS'}$), 125.8 (C-3‘), 122.4 (C-11), 121.3 (C-10), 117.6 (*C*N), 113.8 (2C, C-3,5^{PMB}), 110.0 (C-8), 80.6 (C-6), 71.9 (CH_2^{PMB}), 65.2 (C-1‘), 64.9 (C-4‘), 64.4 (C-2), 63.1 (C-3a), 57.3 (CH_2^{Bn}), 55.2 (CH_3^{PMB}), 54.6 (C-11b), 44.8 (C-1), 36.3 (C-5), 34.8 (C-4), 26.8 (3C, $C(CH_3)_3^{TBDPS}$), 19.2 (C_q^{TBDPS}) ppm.

IR (ATR): $\tilde{\nu}$ [cm^{-1}] = 2931, 2855, 2192, 1722, 1643, 1613, 1512, 1465, 1247, 1108.

ESI-MS (+ / *m/z* in %): 816.4 (100) $[M+H]^+$

HRMS (ESI): found 816.3825, calc for $[C_{51}H_{54}N_3O_5Si]^+$: 816.3827

Melting range: 66.3-68.3 (MeCN/H_2O)

$[\boldsymbol{\alpha}]_{\boldsymbol{D}}^{\mathbf{21}} = -189{,}7$ (c=0.33, $CHCl_3$)

Synthesis of 16

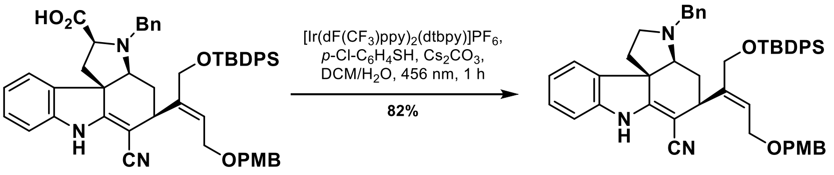

15 16

In a 20 mL microwave reaction vessel **15** (1.46 g, 1.79 mmol, 1.0 eq.), $[Ir(dF(CF_3)ppy)_2(dtbpy)]PF_6$ (40 mg, 0.036 mmol, 2 mol%), Cs_2CO_3 (826 mg, 2.14 mmol, 1.2 eq.) and 4-chlorothiophenol (26 mg, 0.18 mmol, 0.1 eq.) were dissolved in DCM/H_2O (9:1, 17.8 mL) and degassed by sparging with argon for 5 min. The tube was sealed and the mixture was irradiated with a Kessil Spotlight (456 nm, 25%-intensity) under vigorous stirring for 1 h. The mixture was diluted with H_2O and extracted three times with EtOAc. The combined organics were dried over Na_2SO_4 and the solvent was removed under reduced pressure. The residue was purified by column chromatography (silica, cHex/EtOAc 6:1) and the product was obtained as a colorless foam (1.13 g, 1.46 mmol) in a yield of 82%.

$\boldsymbol{R_f}$ = 0.41 (cHex/EtOAc 4:1) [Seebach]

^{1}H-NMR, COSY (600 MHz, $CDCl_3$) δ 7.70 (ddd, *J* = 8.0, 2.4, 1.5 Hz, 4H, H-2,6^{TBDPS}, H-2,6$^{TBDPS'}$), 7.62 – 7.58 (m, 1H, H-11), 7.44 – 7.41 (m, 2H, H-4^{TBDPS}, H-4$^{TBDPS'}$), 7.41 – 7.36 (m, 5H, H-2,6^{Bn}, H-3,5^{TBDPS}, H-3,5$^{TBDPS'}$), 7.35 – 7.32 (m, 2H, H-3,5^{Bn}), 7.27 (d, *J* = 2.6 Hz, 2H, H-2,6^{PMB}), 7.26 (s, 1H, H-4^{Bn}), 7.14 (td, *J* = 7.7, 1.3 Hz, 1H, H-9), 6.92 (td, *J* = 7.5, 1.0 Hz, 1H, H-10), 6.86 – 6.82 (m, 2H, H-3,5^{PMB}), 6.75 (dd, *J* = 7.7, 0.9 Hz, 1H, H-8), 6.65 (s, 1H, NH), 6.09 – 6.03 (m, 1H, H-3'), 4.50 (d, *J* = 11.5 Hz, 1H, H_a-CH_2^{PMB}), 4.45 (d, *J* = 11.5 Hz, 1H, H_b-CH_2^{PMB}), 4.27 (dd, *J* = 13.9, 1.8 Hz, 1H, H_a-1'), 4.18 (dd, *J* = 12.9, 1.5 Hz, 2H, H_b-1'), 4.17 – 4.15 (m, 2H, CH_2-4'), 3.85 (d, *J* = 13.2 Hz, 1H, H_a-CH_2^{Bn}), 3.74 (s, 3H, OCH_3^{PMB}), 3.72 (d, *J* = 13.2 Hz, 1H, H_b-CH_2^{Bn}), 3.50 (dd, *J* = 10.8, 6.3 Hz, 1H, H-5), 3.25 (dd, *J* = 11.0, 4.7 Hz, 1H, H-3a), 3.04 – 2.97 (m, 1H, H_a-2), 2.50 (td, *J* = 10.1, 3.2 Hz, 1H, H_b-2), 2.03 – 1.97 (m, 1H, H_a-1), 1.89 (ddd, *J* = 13.8, 6.3, 4.7 Hz, 1H, H_a-4), 1.68 (ddd, *J* = 12.3, 8.6, 3.2 Hz, 1H, H_b-1), 1.60 (m, 1H, H_b-4 [behind residual water]), 1.09 (s, 9H, $C(CH_3)_3$) ppm.

^{13}C-NMR, HSQC, HMBC (151 MHz, $CDCl_3$) δ 165.0 (C-6a), 159.1 (C-4^{PMB}), 142.6 (C-7a), 141.7 (C-2'), 139.2 (C-1^{Bn}), 137.1 (C-11a), 135.6 (2C, C-2,6^{TBDPS}), 135.6 (2C, C-2,6$^{TBDPS'}$), 133.4 (C-1^{TBDPS}), 133.2 (C-1$^{TBDPS'}$), 130.3 (C-1^{PMB}), 129.8 (C-4^{TBDPS}), 129.8 (C-4$^{TBDPS'}$), 129.5 (2C, C-2,6^{PMB}), 128.4 (2C, C-3,5^{Bn}), 128.3 (2C, C-2,6^{Bn}), 127.9 (C-9), 127.8 (2C, C-3,5^{TBDPS}), 127.7 (2C, C-3,5$^{TBDPS'}$), 127.0 (C-4^{Bn}), 124.9 (C-3'), 123.5 (C-11), 121.5 (C-10), 118.3 (*C*N), 113.8 (2C, C-3,5^{PMB}), 109.1 (C-8), 77.1 (C-6), 71.7 (CH_2^{PMB}), 65.0 (C-4'), 64.7 (C-1'), 62.2 (C-3a), 55.2 (OCH_3^{PMB}), 54.7 (CH_2^{Bn}), 53.1 (C-11b), 47.7 (C-2), 40.1 (C-1), 36.2 (C-5), 26.9 (3C, $C(CH_3)_3$), 25.8 (C-4), 19.3 ($C(CH_3)_3$) ppm.

IR (ATR): $\tilde{\nu}$ [cm^{-1}] = 2932, 2856, 2193, 1644, 1614, 1513, 1465, 1248, 1162, 1107.

HRMS (ESI): found 772.3932, calc for $[C_{50}H_{54}N_3O_3Si]^+$: 772.3929.

Melting range: 60.3-63.3 °C [cHex/EtOAc]

$[\boldsymbol{\alpha}]_{\boldsymbol{D}}^{\boldsymbol{21}} = -250.8$ (c=0.24) [$CHCl_3$]

Synthesis of 3

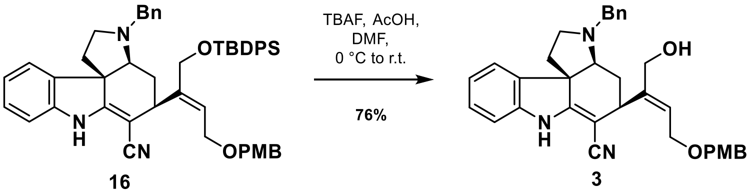

To a solution of **16** (220 mg, 0.285 mmol, 1.0 eq.) in dry DMF (2.8 mL) at 0 °C was added AcOH (48 µL, 0.85 mmol, 3.0 eq.) and dropwise TBAF (1 m in THF, 285 µL, 0.285 mmol, 1.0 eq.). The solution was stirred for 1 h at 0 °C and 2 h at rt. Water was added and the mixture was extracted three times with EtOAc. The combined organics were dried over Na_2SO_4 and the solvent was removed under reduced pressure. The residue was purified by column chromatography (silica, cHex/acetone 2:1) The product was obtained as a yellow foam (106 mg, 0.198 mmol, 70%) contaminated with 10% of an impurity that could not be removed by chromatography.

(The impurity is probably the amide derived from nitrile hydrolysis) $\boldsymbol{R_f}$ = 0.23 (cHex/EtOAc 2:1)[Seebach]

^{1}H-NMR, COSY (400 MHz, $CDCl_3$) δ 7.45 (dd, *J* = 7.5, 1.3 Hz, 1H, H-11), 7.42 – 7.31 (m, 4H, H-2,3,5,6^{Bn}), 7.30 (m, 1H, H-4^{Bn}), 7.29 – 7.22 (m, 2H, H-2,6^{PMB}), 7.16 (td, *J* = 7.7, 1.3 Hz, 1H, H-9), 7.01 (s, 1H, N*H*), 6.92 (td, *J* = 7.5, 1.0 Hz, 1H, H-10), 6.86 – 6.82 (m, 2H, H-3,5^{PMB}), 6.81 (d, *J* = 7.9 Hz, 1H, H-8), 5.96 (t, *J* = 6.4 Hz, 1H, H-3’), 4.54 – 4.43 (m, 2H, Ha-CH_2^{PMB}), 4.27 – 4.18 (m, 2H, H_2-1’), 4.15 (dd, *J* = 6.4, 1.9 Hz, 2H, H_2-4’), 3.86 (s, 2H, CH_2^{Bn}), 3.74 (s, 3H, CH_3), 3.59 (t, *J* = 7.6 Hz, 1H, H-5), 3.35 (t, *J* = 6.9 Hz, 1H, H-3a), 3.02 (dt, *J* = 9.5, 8.1 Hz, 1H, H_a-2), 2.80 (td, *J* = 9.5, 2.6 Hz, 1H, H_b-2), 2.37 (dt, *J* = 12.5, 9.1 Hz, 1H, H_a-1), 1.90 (t, *J* = 7.3 Hz, 2H, H_2-4), 1.80 (ddd, *J* = 12.4, 7.7, 2.5 Hz, 1H, H_b-1) ppm.

^{13}C-NMR, HSQC, HMBC (101 MHz, $CDCl_3$) δ 166.1 (C-6a), 159.2 (C-4^{PMB}), 142.8 (C-2’), 142.6 (C-7a), 138.2 (C-1^{Bn}), 137.0 (C-11a), 130.1 (C-1^{PMB}), 129.5 (2C, C-2,6^{PMB}), 128.8 (2C, C-2,6^{Bn}), 128.5 (2C, C-3,5^{Bn}), 128.0 (C-9), 127.5 (C-3’), 127.3 (C-4^{Bn}), 123.0 (C-11), 121.5 (C-10), 118.7 (*C*N), 113.8 (2C, C-3,5^{PMB}), 109.3 (C-8), 76.7 (C-6), 72.1 (*C*H_2^{PMB}), 65.2 (C-4’), 65.0 (C-1’), 63.0 (C-3a), 55.7 (CH_2^{Bn}), 55.2 (CH_3), 53.5 (C-11b), 48.6 (C-2), 40.8 (C-1), 36.7 (C-5), 27.2 (C-4) ppm.

IR (ATR): $\tilde{\nu}$ [cm^{-1}] = 3302, 2909, 2836, 2192, 1642, 1612, 1513, 1464, 1349, 1247.

ESI-MS (+ / *m/z* in %): 534.3 (100) $[M+H]^+$.

HRMS (ESI): found 534.2753, calc for $[C_{34}H_{36}N_3O_3]^+$: 534.2751.

$[\boldsymbol{\alpha}]_{\boldsymbol{D}}^{\boldsymbol{21}} = -340$ (c = 0.54, $CHCl_3$)

Synthesis of **17**

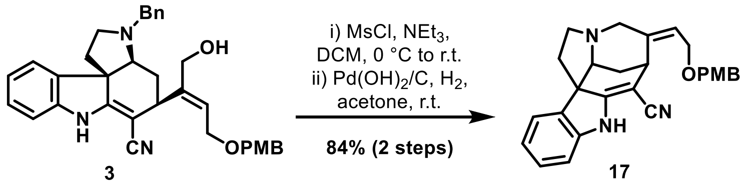

To a solution of **3** (100 mg, 0.187 mmol, 1.0 eq.) in dry DCM (1.9 mL) was added at 0 °C NEt_3 (51 µL, 0.38 mmol, 2.0 eq.) and MsCl (29 µL, 0.38 mmol, 2.0 eq.). The solution was stirred for 1 h at 0 °C and 2 h at rt. The reaction was quenched with water and the mixture was extracted three times with DCM. The combined organics were dried over $MgSO_4$ and the solvent was removed under reduced pressure. The residue was dissolved in degassed $CHCl_3$ (13 mL) and Pearlman´s catalyst (20 wt% on C, 127 mg) was added. The reaction mixture was set under an atmosphere of hydrogen (double-walled balloon) and was vigorously stirred for 30 min. The mixture was filtered through a pad of Celite which was rinsed with $CHCl_3$ and MeOH (+10% NH_4OH_{aq}) and the solvent was removed under reduced pressure. The residue was purified by column chromatography (silica, DCM/MeOH (+10% NH_4OH_{aq}) 95:5). The product was obtained as a yellowish oil (67 mg, 0.16 mmol) in a yield of 84% over two steps.

$\boldsymbol{R_f}$ = 0.23 (DCM/MeOH(+10% NH_4OH) 95:5)

^{1}H-NMR, COSY (600 MHz, $CDCl_3$) δ 7.33 – 7.30 (m, 2H, H-2',6'), 7.23 (dd, *J* = 7.5, 1.2 Hz, 1H, H-9), 7.18 (td, *J* = 7.7, 1.2 Hz, 1H, H-11), 6.94 (td, *J* = 7.5, 0.9 Hz, 1H, H-10), 6.92 (s, 1H, N*H*), 6.90 – 6.87 (m, 2H, H-3',5'), 6.84 (dt, *J* = 7.9, 0.8 Hz, 1H, H-12), 5.59 (tt, *J* = 5.4, 2.3 Hz, 1H, H-19), 4.52 (d, *J* = 1.3 Hz, 2H, CH_2^{PMB}), 4.29 (ddd, *J* = 12.4, 7.5, 1.7 Hz, 1H, H_a-18), 4.19 (ddt, *J* = 12.3, 5.3, 1.5 Hz, 1H, H_b-18), 4.15 (d, *J* = 3.9 Hz, 1H, H-3), 3.89 (dt, *J* = 15.6, 1.8 Hz, 1H, H_b-21), 3.80 (s, 3H, CH_3^{PMB}), 3.50 (s, 1H, H_b-15), 3.21 (ddd, *J* = 11.6, 9.2, 6.0 Hz, 1H, H_b-5), 3.02 (d, *J* = 15.8 Hz, 1H, H_a-21), 2.93 (ddd, *J* = 11.4, 6.4, 4.6 Hz, 1H, H_a-5), 2.37 (dt, *J* = 13.8, 3.3 Hz, 1H, H_a-14), 2.24 (ddd, *J* = 12.6, 9.2, 6.4 Hz, 1H, H_a-6), 1.94 (ddd, *J* = 12.6, 6.0, 4.6 Hz, 1H, H_b-6), 1.40 (dt, *J* = 13.8, 2.8 Hz, 1H, H_b-14) ppm.

^{13}C-NMR, HSQC, HMBC (151 MHz, $CDCl_3$) δ 168.6 (C-2), 159.2 (C-4'), 143.1 (C-13), 138.5 (C-20), 135.0 (C-8), 130.2 (C-1'), 129.8 (2C, C-2',6'), 128.3 (C-11), 125.5 (C-19), 121.6 (C-10), 121.3 (C-9), 119.2 (C-17), 113.8 (2C, C-3',5'), 109.7 (C-12), 81.4 (C-16), 72.7 ($\mathit{C}H_2^{PMB}$), 66.2 (C-18), 60.0 (C-3), 56.8 (C-7), 55.3 (CH_3^{PMB}), 54.5 (C-21), 54.3 (C-5), 45.1 (C-6), 32.4 (C-15), 29.2 (C-14) ppm.

IR (ATR): $\tilde{\nu}$ [cm^{-1}] = 3292, 2923, 2852, 2186, 1636, 1612, 1513, 1465, 1355, 1247.

ESI-MS (+ / *m/z* in %): 426.3 (100) $[M+H]^+$

HRMS (ESI): found 426.2172, calcd for $[C_{27}H_{28}N_3O_2]^+$: 426.2176.

$[\boldsymbol{\alpha}]_{\boldsymbol{D}}^{\boldsymbol{21}} = -425.3$ (c=0.43, $CHCl_3$)

Synthesis of 18

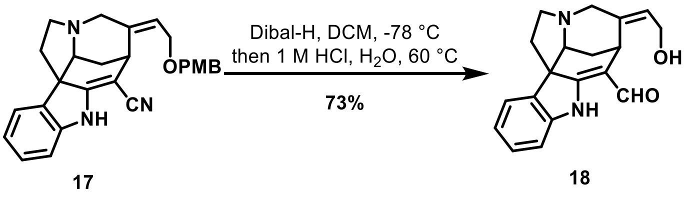

To a solution of **17** (10 mg, 0.024 mmol, 1.0 eq.) in dry DCM (0.5 mL) was added Dibal-H (1 M in toluene, 55 µL, 0.055 mmol, 2.3 eq.) at −78 °C. The bright yellow solution was stirred at −78 °C for 2 h and was quenched at that temperature by the addition of aqueous 1 M HCl (2 mL). The solution was warmed to rt. and stirred for 16 h and afterwards for 5 h at 60 °C. At rt. a conc. NH_4OH solution was added and the mixture was extracted three times with DCM. The combined organics were dried over $MgSO_4$ and the solvent was removed under reduced pressure. The residue was purified by column chromatography (silica, DCM/MeOH (+10% NH_4OH_{aq}) 9:1). The product was obtained as a yellow solid in 73% yield.

RR_f = 0.26 (DCM/MeOH (+10% NH_4OH_{aq}) 9:1)

^{1}H-NMR, COSY (600 MHz, $CDCl_3$) δ 10.37 (s, 1H, NH), 9.32 (s, 1H, H-17), 7.30 (dd, *J* = 7.5, 1.1 Hz, 1H, H-9), 7.21 (td, *J* = 7.7, 1.2 Hz, 1H, H-11), 7.00 (td, *J* = 7.5, 1.0 Hz, 1H, H-10), 6.93 (d, *J* = 7.8 Hz, 1H, H-12), 5.53 (tt, *J* = 6.2, 2.0 Hz, 1H, H-19), 4.29 – 4.20 (m, 2H, H-18), 4.11 (dt, *J* = 3.8, 2.0 Hz, 1H, H-3), 4.03 (dt, *J* = 15.8, 2.0 Hz, 1H, H_a-21), 3.78 (s, 1H, H-15), 3.33 (td, *J* = 12.5, 5.4 Hz, 1H, H_a-5), 3.10 (dd, *J* = 12.4, 6.5 Hz, 1H, H_b-5), 3.00 (d, *J* = 15.7 Hz, 1H, H_b-21), 2.58 (dt, *J* = 13.7, 4.0 Hz, 1H, H_a-14), 2.39 (td, *J* = 12.5, 6.6 Hz, 1H, H_a-6), 1.86 (dd, *J* = 12.5, 5.4 Hz, 1H, H_b-6), 1.29 (ddd, *J* = 13.7, 3.7, 2.1 Hz, 1H, H_b-14) ppm.

^{13}C-NMR, HSQC, HMBC (151 MHz, $CDCl_3$) δ 188.2 (C-17), 169.6 (C-2), 142.8 (C-13), 141.8 (C-20), 137.0 (C-8), 128.3 (C-11), 125.9 (C-19), 122.6 (C-10), 121.3 (C-9), 111.5 (C-16), 110.8 (C-12), 61.8 (C-3), 58.7 (C-18), 58.6 (C-7), 56.9 (C-5), 56.8 (C-21), 46.5 (C-6), 31.4 (C-15), 31.1 (C-14) ppm.

IR (ATR): $\tilde{\nu}$ [cm^{-1}] = 3298, 2943, 2870, 1641, 1607, 1588, 1550, 1463, 1377, 1186.

ESI-MS (+ / *m/z* in %): 309.1 (100) $[M+H]^+$

HRMS (ESI): found 309.1590, calc for $[C_{19}H_{21}N_2O_2]^+$: 309.1598.

$[\alpha]_D^{22} = -1282.9$ (c=0.38, MeOH)

Table NMR comparison of Strychnofluorine (18)

Table 1: Comparison of NMR Data of Strychnofluorine (**18**) (Lit. Angenot 1992[61]).

Position	^{13}C Lit.[61] (100 MHz, MeOH-d_4^{a})	^{13}C (151 MHz, $CDCl_3$)	^{1}H Lit.[61] (400 MHz, $CDCl_3$)	^{1}H (600 MHz, $CDCl_3$)
1(NH)			10.37br s	10.37 (s)
2	169.7	169.6		
3	61.8	61.8	4.09 s	4.11 (dt, *J* = 3.8, 2.0 Hz)
5	56.8	56.9		
5a			3.32 td(12.4, 12.4, 5.2)	3.33 (td, *J* = 12.5, 5.4 Hz)

5b			3.09 dd (12.4, 6.5)	3.10 (dd, *J* = 12.4, 6.5 Hz)
6	46.6	46.5		
6a			2.41 td (12.4, 6.5, 12.4)	2.39 (td, *J* = 12.5, 6.6 Hz)
6b			1.86 dd (5.2, 12.4)	1.86 (dd, *J* = 12.5, 5.4 Hz)
7	--*	58.6		
8	137.1	137.0		
9	121.3	121.3	7.30 d (7.4)	7.30 (dt, *J* = 7.5, 1.1 Hz)
10	122.6	122.6	7.00 t (7.4)	7.00 (td, *J* = 7.5, 1.0 Hz)
11	131.1	128.3	7.21 td (7.4, 7.7)	7.21 (td, *J* = 7.7, 1.2 Hz)
12	110.9	110.8	6.93 d (7.7)	6.93 (d, *J* = 7.8)
13	142.9	142.8		
14	31.1	31.1		
14a			2.57 dq (13.6, 2.8, 2.1)	2.58 (ddd, *J* = 13.7, 4.0, 2.2 Hz)
14b			1.29 dd (13.6)	1.29 (ddd, *J* = 13.7, 3.7, 2.1 Hz)
15	31.5	31.4	3.78 br s	3.78 (s)
16	111.4	111.5		
17	188.2	188.2	9.32 s	9.32 (s)
18	58.6	58.7	4.24 d (6.2)	4.29 – 4.20 (m, 2H)
19	125.9	125.9	5.53 t (6.2)	5.59 – 5.49 (tt, 6.2, 2.0 Hz)
20	141.8	141.8		
21	56.9	56.8		
21a			4.00 d (15.8)	4.03 (dt, *J* = 15.8, 2.0 Hz)
21b			2.97 d (15.8)	3.00 (d, *J* = 15.7 Hz)
OH			2.07 br s	-#

[a] we suspect the authors measured in $CDCl_3$ but wrongly showed MeOH-d_4 * The authors have not reported a signal for C-7. #labile OH was not detected

Synthesis of strychnine (**1**)

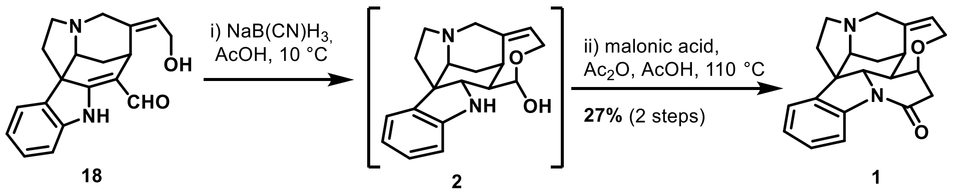

Based on a method of Levy.[62]

To a solution of strychnofluorine (**18**, 9.0 mg, 0.029 mmol) in AcOH (0.3 mL) at 15 °C was added $NaBH_3CN$ (3.7 mg, 0.058 mmol, 4.0 eq.). The solution was stirred for 30 min at 15 °C and quenched with conc. NH_4OH_{aq}. The mixture was extracted three times with DCM and the combined organic extracts were dried over Na_2SO_4. The solvent was removed under reduced pressure. The crude Wieland-Gumlich aldehyde was used without further purification in the next step.

(Note: NMR and HPLC/MS analysis of the crude extract showed Wieland Gumlich aldehyde **(2)** *contaminated with the diol overreduction product.* ^{1}*H NMR (***2***: 5.84 ppm,* ***diol****: 5.50 ppm) and LCMS ([M+H] +* ***2****: 311,* ***diol****: 313) which was observed earlier.* [29]*)*

Based on a method of Robinson.[63]

The crude was dissolved in AcOH (0.6 mL) and malonic acid (20 mg), NaOAc (40 mg) and Ac_2O (6.6 µL) were added. The mixture was stirred at 110 °C for 2 h and afterwards cooled to rt. 50% NaOH was added and the basified mixture was extracted four times with DCM. The combined organics were dried over Na_2SO_4 and the solvent was removed under reduced pressure. The residue was purified by column chromatography (silica, DCM/NEt_3 100:5). The product was obtained as a colourless amorphous solid (3.6 mg, 0.018 mmol) in a yield of 37% over two steps.

$\boldsymbol{R_f}$ = 0.42 (DCM/NEt_3 100:5)

^{1}H-NMR, COSY (600 MHz, $CDCl_3$) δ 8.09 (d, *J* = 8.0 Hz, 1H, H-12), 7.25 (ddd, *J* = 8.0, 7.4, 1.4 Hz, 1H, H-11), 7.16 (dd, *J* = 7.6, 1.3 Hz, 1H, H-9), 7.10 (td, *J* = 7.4, 1.1 Hz, 1H, H-10), 5.90 (tt, *J* = 6.8, 2.5 Hz, 1H, H-19), 4.28 (dt, *J* = 8.4, 3.3 Hz, 1H, H-17), 4.14 (dd, *J* = 13.8, 7.0 Hz, 1H, H_a-18), 4.06 (ddd, *J* = 14.0, 6.2, 1.0 Hz, 1H, H_b-18), 3.94 (dd, *J* = 4.1, 2.2 Hz, 1H, H-3), 3.85 (d, *J* = 10.5 Hz, 1H, H-2), 3.70 (dq, *J* = 14.8, 1.7 Hz, 1H, H_a-21), 3.29 – 3.17 (m, 1H, H_a-5), 3.15 (s, 1H, H-15), 3.13 (dd, *J* = 17.3, 8.5 Hz, 1H, H_a-23), 2.87 (dd, *J* = 19.2, 10.4 Hz, 1H, H_b-5), 2.73 (d, *J* = 14.8 Hz, 1H, H_b-21), 2.67 (dd, *J* = 17.4, 3.3 Hz, 1H, H_b-23), 2.36 (dt, *J* = 14.4, 4.4 Hz, 1H, H_a-14), 1.91 – 1.87 (m, 2H, H_2-6), 1.46 (d, *J* = 14.4 Hz, 1H, H_b-14), 1.27 (dt, *J* = 10.5, 3.2 Hz, 1H, H-16) ppm.

^{13}C-NMR, HSQC, HMBC (151 MHz, $CDCl_3$) δ 169.3 (C-24), 142.2 (C-13), 140.5 (C-20), 132.7 (C-8), 128.6 (C-11), 127.4 (C-19), 124.2 (C-10), 122.3 (C-9), 116.2 (C-12), 77.6 (C-17), 64.6 (C-18), 60.2 (C-3), 60.1 (C-2), 52.6 (C-21), 51.9 (C-7), 50.3 (C-5), 48.2 (C-16), 42.8 (C-6), 42.5 (C-23), 31.6 (C-15), 26.8 (C-14) ppm.

IR (ATR): $\tilde{\nu}$ [cm^{-1}] = 2925, 2854, 1668, 1478, 1460, 1391, 1289, 1108, 1097, 755.

ESI-MS (+ / *m/z* in %): 355.2 (100) $[M+H]^+$

HRMS (ESI): found 335.1753, calc for $[C_{21}H_{23}N_2O_2]^+$: 335.1754.

Optical rotation $[\alpha]_D^{30} = -129.6$ (c=0.27, $CHCl_3$).

NMR and MS data matched the ones of other synthetic procedures.

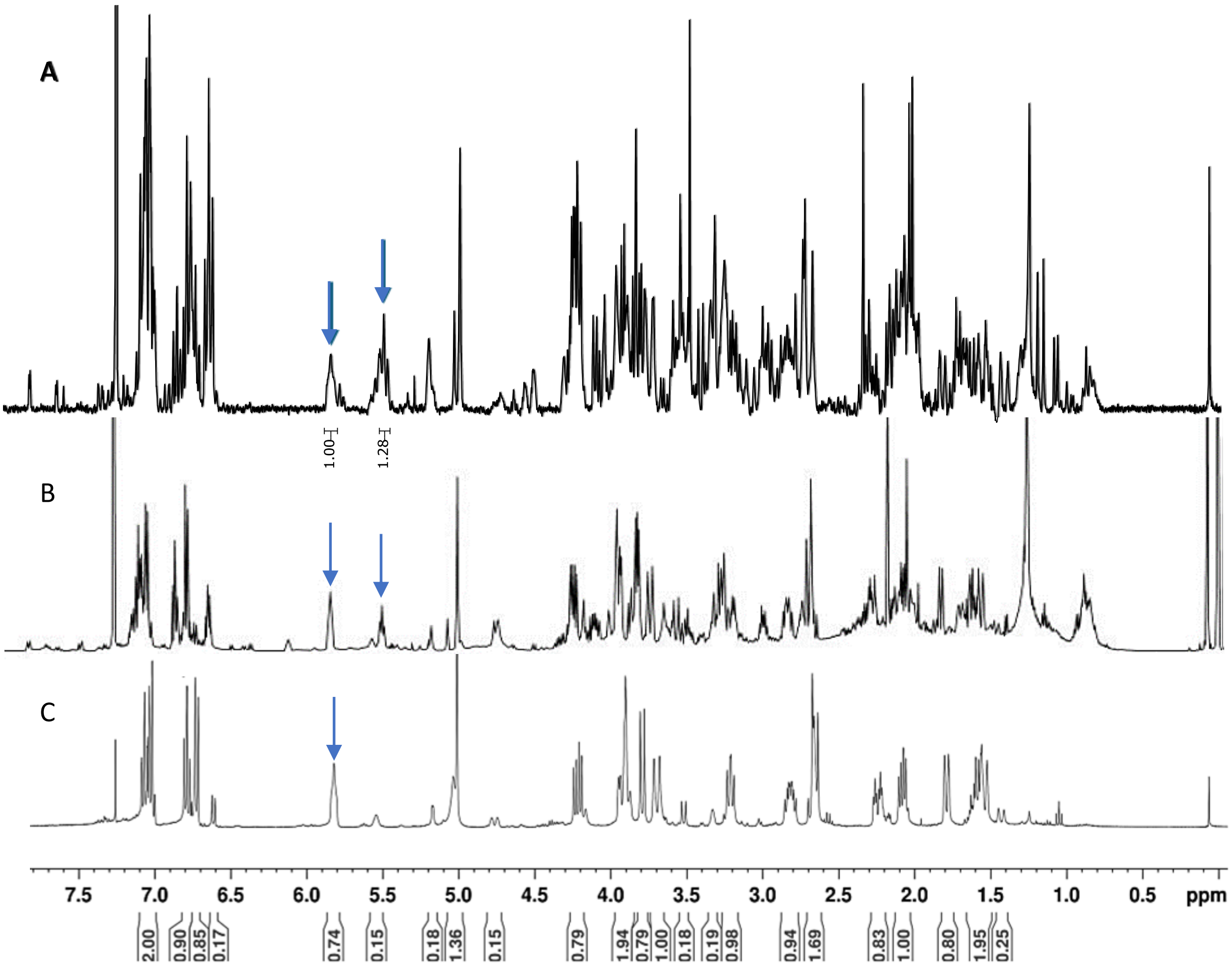

Figure 2: Comparison of Wieland-Gumlich aldehyde (**2**) containing NMR-spectra. A: own reaction mixture; B: reaction mixture of Chen et al.[29]; C: purified Wieland Gumlich aldehyde by O´Connor et al.[64] Significant signals for Wieland Gumlich aldehyde (**2**) and Wieland Gumlich Diol are highlighted.

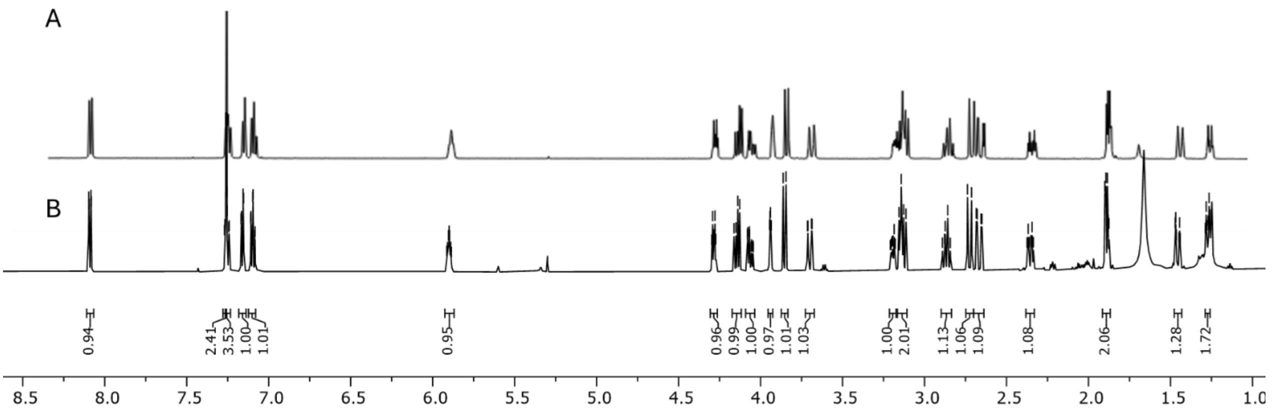

Figure 3: comparison of ^{1}H NMR spectra. A: MacMillan's synthetic strychnine.[26] B: our synthetic strychnine.

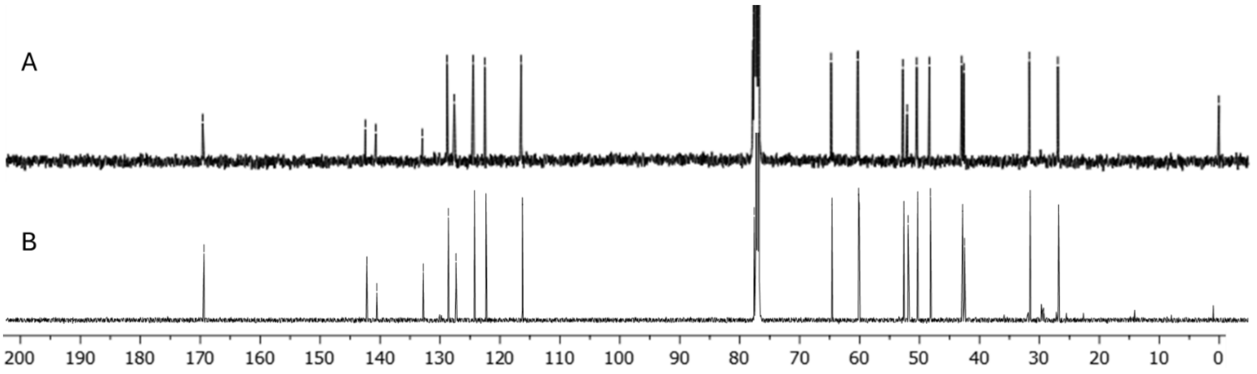

Figure 4: Comparison of ^{13}C NMR spectra. A: Lan/Zhang's synthetic strychnine.[35] B: our synthetic strychnine.

Characterization for S2

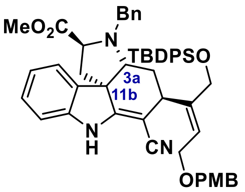

S2

Compound **S2** was obtained as a side product of the condensation-electrocyclization in toluene and subsequent deprotection with TBAF in THF. The compound was isolated in 8% yield over two steps by column chromatography (silica, cHex/EtOAc 3:2) as a colorless oil (110 mg, 186 µmol).

$\boldsymbol{R_f}$ = 0.34 (cHex/EtOAc 1:1).

^{1}H-NMR, COSY (600 MHz, 323 K, CD_3CN): d = 7.89 (s, 1H, NH), 7.52 (d, 1H, J =7.5 Hz, H-11), 7.39–7.36 (m, 2H, H-2', H-6'), 7.32–7.28 (m, 2H, H-3'–H-5'), 7.26–7.24 (m, 2H, H-2''', H-6'''), 7.24–7.22 (m, 1H, H-4'), 7.14 (td, 1H, J = 7.7 Hz, 1.3 Hz, H-9), 6.90 (td, 1H, J = 7.7 Hz, 1.3 Hz, H-10), 6.85 (dt, 1H, J = 7.7 Hz, 1.0 Hz, H-8), 6.84–6.80 (m, 2H, H-3''', H-5'''), 5.87 (tt, 1H,J = 6.8 Hz, 1.5 Hz, H-3''), 4.46 (d, 1H, J = 11.6 Hz, Ha-1'''a), 4.43 (d, 1H, J = 11.4 Hz, Hb-1'''a), 4.12 (d, 2H, J = 6.8 Hz, H2-4''), 4.07 (d, 1H, J = 13.8 Hz, Ha-1'a), 4.04 (d, 1H, J = 13.8 Hz, Hb-1'a), 4.02 (d, 1H, J = 14.6 Hz, Ha-1''), 3.71 (s, 3H, 4'''-OCH3), 3.70–3.69 (m, 1H, H-3a), 3.67 (dd, 1H, J = 9.4 Hz, 3.0 Hz, H-2), 3.60 (s, 3H, CO2CH3), 3.59–3.55 (m, 1H, H-5),

2.74 (s, 1H, OH), 2.41 (dd, 1H, J =13.4 Hz, 9.4 Hz, Ha-1), 2.05 (dd, 1H, J = 13.4 Hz, 3.0 Hz, Hb-1), 1.98 (ddd, 1H, J = 13.9 Hz, 3.8 Hz, 2.4 Hz, Ha-4), 1.26 (ddd, 1H, J = 13.9 Hz, 10.8 Hz, 5.5 Hz, Hb-4) ppm.

^{13}C-NMR, HSQC, HMBC (151 MHz, 323 K, CD_3CN): d = 174.9 (CO2CH3), 168.1 (Cq-6a), 160.4 (Cq-4'''), 144.8 (Cq-7a), 143.5 (Cq-2''), 140.7 (Cq-1'), 137.6 (Cq-11a), 132.0 (Cq-1'''), 130.5 (2C, C-2''', C-6'''), 129.7 (2C, C-2', C-6'), 129.4 (2C, C-3', C-5'), 129.1 (C-9), 128.1 (C-4'), 126.1 (C-3''), 124.5 (C-11), 121.9 (C-10), 119.0 (CN), 114.8 (2C, C-3''', C-5'''), 110.4 (C-8), 79.4 (Cq-6), 72.6 (C-1'''a), 66.6 (C-4''), 64.2 (C-1''), 63.9 (C-3a), 62.8 (C-2), 56.0 (4'''-OCH3), 55.4 (Cq-11b), 52.7 (C-1'a), 52.3 (CO2CH3), 47.9 (C-1), 33.4 (C-5), 30.7 (C-4) ppm.

IR (ATR): $\tilde{\nu}$ [cm^{-1}] = 3295, 3000, 2949, 2855, 2190, 1733, 1636, 1613, 1467, 1247, 1156.

ESI-MS (+ / *m/z* in %): 592.3 (100) $[M+H]^+$

HRMS (ESI): found 592.2806, calc for $[C_{36}H_{38}N_3O_5]^+$: 592.2806.

$[\alpha]_D^{21}$ = +85.7 (c=0.3 in MeOH)

Synthesis of S3

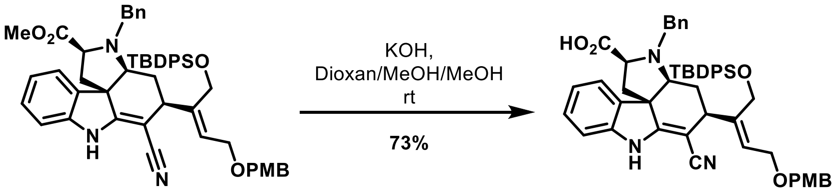

(*Z*)-14 S3

To a solution of **(*z*)-14** (2.94 g, 3.54 mmol, 1.0 eq.) in dioxane (14 mL)/MeOH (14 mL) and H_2O (2 mL) was added a solution of KOH (10% in H_2O, 5.0 mL, 8.8 mmol, 2.5 eq.). The solution was stirred for 18 h at rt and was acidified with 1 M HCl and extracted three times with EtOAc. The combined organics were dried over $MgSO_4$ and the solvent was removed under reduced pressure. The crude was purified by column chromatography (silica, cHex/acetone 4:1) and the product was obtained as a colorless solid (2.12 g, 2.60 mmol, 73%)

R_f = 0.23 (cHex:acetone = 3:1)

^{1}H-NMR, COSY (400 MHz, $CDCl_3$) δ 7.77 – 7.72 (m, 2H, H-2^{TBDPS}, H-6^{TBDPS}), 7.69 – 7.64 (m, 2H, H-$2'^{TBDPS}$, H-$6'^{TBDPS}$), 7.47 – 7.37 (m, NH, H-3^{TBDPS}, H-$3'^{TBDPS}$, H-4^{TBDPS}, H-$4'^{TBDPS}$, H-5^{TBDPS}, H-$5'^{TBDPS}$), 7.36 (dd, *J* = 5.6, 1.6 Hz, 3H, H-3^{Bn}, H-4^{Bn}, H-5^{Bn}), 7.17 (d, *J* = 8.7 Hz, 2H, H-2^{PMB}, H-6^{PMB}), 7.18 – 7.13 (m, 2H, H-2^{Bn}, H-6^{Bn}), 7.10 (td, *J* = 7.7, 1.1 Hz, 1H, H-9), 6.83 – 6.77 (m, 3H, H-3^{PMB}, H-5^{PMB}, H-8), 6.70 (td, *J* = 7.6, 1.0 Hz, 1H, H-10), 6.18 (dd, *J* = 7.6, 1.1 Hz, 1H, H-11), 5.74 – 5.58 (m, 1H, H-3'), 4.51 (d, *J* = 12.7 Hz, 1H, H_a-1'), 4.32 (d, *J* = 11.4 Hz, 1H, H_a-CH_2^{PMB}), 4.25 (d, *J* = 12.2 Hz, 1H, H_b-1'), 4.25 (d, *J* = 11.3 Hz, 1H, H_b-CH_2^{PMB}), 3.95 – 3.80 (m, 2H, H_2-4'), 3.77 (s, 3H, CH_3^{PMB}), 3.76 – 3.73 (m, 1H, H-5), 3.69 (d, *J* = 13.7 Hz, 1H, H_a-CH_2^{Bn}), 3.65 (dd, *J* = 11.7, 5.6 Hz, 1H, H-2), 3.61 (d, *J* = 13.7 Hz, 1H, H_b-CH_2^{Bn}), 3.58 (dd, *J* = 5.7, 1.6 Hz, 1H, H-3a), 2.27 (t, *J* = 12.0 Hz, 1H, H_a-1), 2.21 (dt, *J* = 14.7, 2.1 Hz, 1H, H_a-4), 1.84 (dd, *J* = 12.0, 5.6 Hz, 1H, H_b-1), 1.47 (dt, *J* = 14.7, 5.9 Hz, 1H, H_b-4), 1.08 (s, 9H, $3xCH_3^{tBu}$) ppm.

^{13}C-NMR, HSQC, HMBC (101 MHz, $CDCl_3$) δ 172.1 (*C*O_2H), 165.9 (C-6a), 159.1 (C-4^{PMB}), 142.1 (C-7a), 141.8 (C-2'), 135.8 (C-11a), 135.8 (2C, C-2^{TBDPS}, C-6^{TBDPS}), 135.6 (2C, C-$2'^{TBDPS}$, $6'^{TBDPS}$), 133.3 (C-1^{Bn}), 133.1

(C-1 ^{TBDPS}), 132.8 (C-1$^{'TBDPS}$), 130.6 (2C, C-2^{Bn}, C-6^{Bn}), 130.0 (C-1^{PMB}), 130.0 (C-4 ^{TBDPS}), 130.0 (C-4$^{'TBDPS}$), 129.6 (2C, C-2^{PMB}, C-6^{PMB}), 128.8 (2C, C-3^{Bn}, C-5^{Bn}), 128.6 (C-9), 128.3 (C-4^{Bn}), 128.0 (2C, C-3 ^{TBDPS}, C-5 ^{TBDPS}), 127.9 (2C, C-3$^{'TBDPS}$, C-5$^{'TBDPS}$), 125.8 (C-3'), 121.3 (C-11), 121.2 (C-10), 119.0 (*C*N), 113.8 (2C, C-3^{PMB}, C-5^{PMB}), 109.7 (C-8), 76.6 (C-6), 72.3 (*C*H$_2^{PMB}$), 66.0 (C-4'), 64.0 (C-3a), 63.4 (C-2), 60.6 (C-1'), 55.3 (*C*H$_2^{Bn}$), 55.2 (CH$_3^{PMB}$), 53.4 (C-11b), 46.3 (C-1), 39.6 (C-5), 29.7 (C-4), 26.9 (3C, 3x*C*H$_3^{tBu}$), 19.2 (C$_q^{tBu}$) ppm.

IR (ATR): $\tilde{\nu}$ [cm^{-1}] = 3281, 2930, 2857, 2193, 1719, 1644, 1614, 1513, 1467, 1247.

ESI-MS (+ / *m/z* in %): 560.2 (100) [M-OTBDPS]$^+$.

HRMS (ESI): found 816.3821 [M + H]$^+$ (calc for [$C_{51}H_{54}N_3O_5Si$]$^+$: 816.3827).

Melting range: 83.1-86.2°C (MeCN/H_2O).

$[\alpha]_D^{22}$ = 77.8 (c=0.98, $CHCl_3$)

Synthesis of S4

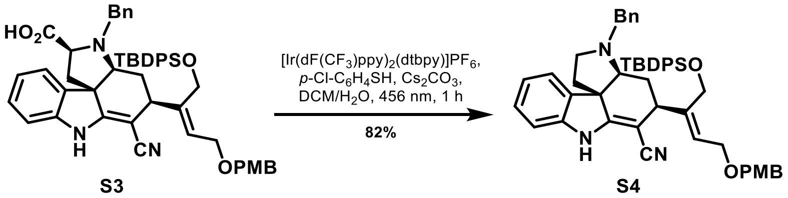

In a Schlenk tube, acid **S3** (816 mg, 1.00 mmol, 1.0 eq.), [Ir(dF(CF_3)ppy)$_2$(dtbpy)]PF_6 (22 mg, 0.02 mmol, 2 mol%), Cs_2CO_3 (391 mg, 1.20 mmol, 1.2 eq.) and 4-chlorothiophenol (15 mg, 0.10 mmol, 0.1 eq.) were dissolved in DCM/H_2O (9:1, 10 mL). The solution was degassed by a stream of argon for five minutes. The vessel was closed and irradiated for 24 h with a Kessil-Spotlight (456 nm, 25% intensity). The reaction mixture was diluted with H_2O and extracted three times with EtOAc. The combined organics were dried over Na_2SO_4 and the solvent was removed under reduced pressure. The crude was purified by column chromatography (silica, cHex/EtOAc 6:1). The product was obtained as a colorless solid (673 mg, 0.871 mmol) in a yield of 87%.

R$_f$ = 0.50 (cHex/EtOAc 4:1)

^{1}H-NMR, COSY (600 MHz, $CDCl_3$) δ 7.67 (dd, *J* = 7.9, 1.4 Hz, 2H, H-2,6^{TBDPS}), 7.64 (dd, *J* = 8.0, 1.4 Hz, 2H, H-2',6'^{TBDPS}), 7.43 – 7.39 (m, 1H, H-4'^{TBDPS}), 7.38 – 7.35 (m, 4H, H-3',5'^{TBDPS}, H-4 ^{TBDPS}), 7.31 (t, *J* = 7.4 Hz, 2H, H-3,5 ^{TBDPS}), 7.25 – 7.19 (m, 6H, H-2,3,4,5,6^{Bn}, H-11), 7.16 (td, *J* = 7.7, 1.2 Hz, 1H, H-9), 7.14 – 7.11 (m, 2H, H-2,6^{PMB}), 6.89 (d, *J* = 1.0 Hz, 1H, H-7), 6.92 – 6.86 (m, 1H, H-10), 6.82 (d, *J* = 7.8 Hz, 1H, H-8), 6.80 – 6.77 (m, 2H, H-3,5^{PMB}), 5.65 (t, *J* = 6.4 Hz, 1H, H-3'), 4.43 (d, *J* = 12.5 Hz, 1H, H$_a$-1'), 4.33 (d, *J* = 12.6 Hz, 1H, H$_b$-1'), 4.27 (q, *J* = 11.4 Hz, 2H, CH$_2^{PMB}$), 3.93 – 3.90 (m, 2H, C*H*$_2$-4'), 3.81 (d, *J* = 13.3 Hz, 1H, H$_a$-CH$_2^{Bn}$), 3.77 (s, 3H, C*H*$_3^{PMB}$), 3.66 (t, *J* = 5.8 Hz, 1H, H-5), 3.57 (d, *J* = 13.4 Hz, 1H, H$_b$-CH$_2^{Bn}$), 3.27 (t, *J* = 5.1 Hz, 1H, H-3a), 2.66 (dt, *J* = 9.5, 4.2 Hz, 2H, H$_2$-2), 2.24 – 2.16 (m, 1H, H$_a$-1), 2.06 (dt, *J* = 14.2, 5.4 Hz, 1H, H$_a$-4), 1.75 (dt, *J* = 14.3, 5.6 Hz, 1H, H$_b$-4), 1.62 (ddd, *J* = 12.1, 5.7, 2.1 Hz, 1H, H$_b$-1), 1.05 (s, 9H, 3xC*H*$_3^{tBu}$) ppm.

^{13}C-NMR, HSQC, HMBC (151 MHz, $CDCl_3$) δ 166.7 (C-6a), 159.0 (C-4^{PMB}), 143.3 (C-2'), 142.4 (C-7a), 138.6 (C-1^{Bn}), 137.6 (C-11a), 135.6 (2C, C-2',6'^{TBDPS}), 135.6 (2C, C-2,6^{TBDPS}), 133.3 (C-1^{TBDPS}), 133.1 (C-1'^{TBDPS}), 130.3 (C-1^{PMB}), 129.8 (2C, C-4^{TBDPS}, C-4'^{TBDPS}), 129.3 (2C, C-2,6^{PMB}), 128.5 (2C, C-2,6^{Bn}), 128.3 (2C, C-3,5^{Bn}),

127.9 (C-9), 127.8 (2C, C-3',5'^{TBDPS}), 127.8 (2C, C-3,5^{TBDPS}), 126.9 (C-4^{Bn}), 124.7 (C-3'), 122.6 (C-11), 121.1 (C-10), 119.2 (*C*N), 113.7 (2C, C-3,5^{PMB}), 109.2 (C-8), 77.3 (C-6), 71.5 (*C*H$_2^{PMB}$), 65.8 (C-4'), 65.0 (C-3a), 60.6 (C-1'), 56.9 (*C*H$_2^{Bn}$), 55.2 (*C*H$_3^{PMB}$), 54.0 (C-11b), 49.7 (C-2), 42.0 (C-1), 40.1 (C-5), 28.4 (C-4), 26.9 (3C, 3xCH$_3^{tBu}$), 19.2 (C$_q^{tBu}$) ppm.

IR (ATR): $\tilde{\nu}$ [cm^{-1}] = 3297, 2930, 2857, 2192, 1644, 1614, 1513, 1466, 1248, 1111.

ESI-MS (+ / *m/z* in %): nd

HRMS (ESI): found 772.3931 [M + H]$^+$ (calc for [$C_{50}H_{54}N_3O_3Si$]$^+$: 772.3929).

Melting range: 55.5-58.6 °C (cHex/EtOAc)

$[\alpha]_D^{22}$ = −87.3 (c=1, $CHCl_3$)

Synthesis of S7b

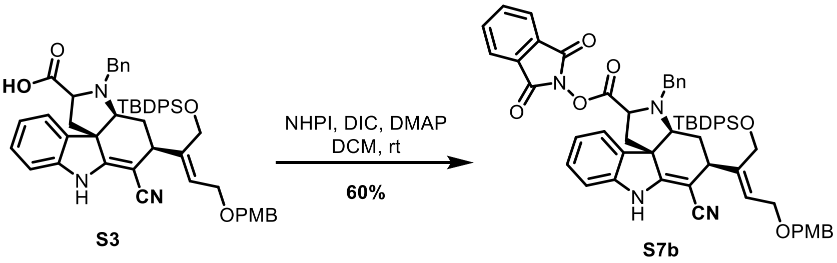

S3 (294 mg, 0.360 mmol, 1.0 eq.), *N*-hydroxyphthalimide (70 mg, 0.43 mmol, 1.2 eq.) and DMAP (4 mg, 0.04 mmol, 0.1 eq.) were dissolved in dry DCM (4 mL) and DIC (64 µL, 0.40 mmol, 1.1 eq.) was added. The solution was stirred at rt for 14 h and water was added. The mixture was extracted three times with EtOAc and the combined organics were dried over Na_2SO_4. The solvent was removed under reduced pressure and the crude was purified by column chromatography (silica, cHex/EtOAc 2:1). The product was obtained as a yellow solid (208 mg, 0.216 mmol, 60%).

$\boldsymbol{R_f}$ = 0.53 (cHex/EtOAc 2:1) [UV]

^{1}H-NMR, COSY (400 MHz, CD_3CN) δ 8.20 (s, 1H, NH), 7.86 (s, 4H, H-Ar^{NHPI}), 7.74 – 7.69 (m, 2H, H-2,6^{TBDPS}), 7.66 – 7.62 (m, 2H, H-2',6'^{TBDPS}), 7.44 – 7.32 (m, 9H, H-3,4,5^{Bn}, H-3,4,5^{TBDPS}, H-3'4'5'^{TBDPS}), 7.12 (td, *J* = 7.7, 1.1 Hz, 1H, H-9), 7.12 – 7.05 (m, 2H, H-2,6^{PMB}), 6.86 (dt, *J* = 7.8, 0.7 Hz, 1H, H-8), 6.76 – 6.71 (m, 2H, H-3,5^{PMB}), 6.68 (td, *J* = 7.5, 1.0 Hz, 1H, H-10), 6.24 (dd, *J* = 7.5, 1.2 Hz, 1H, H-11), 5.54 – 5.46 (m, 1H, H-3'), 4.43 (s, 2H, CH$_2$-1'), 4.19 (d, *J* = 2.2 Hz, 2H, CH$_2^{PMB}$), 3.96 (dd, *J* = 11.4, 5.7 Hz, 1H, H-2), 3.84 (d, *J* = 3.9 Hz, 2H, CH$_2^{Bn}$), 3.77 – 3.72 (m, 1H, H$_a$-4'), 3.71 (s, 3H, CH$_3^{PMB}$), 3.69 – 3.64 (m, 2H, H$_b$-4', H-5), 3.61 (dd, *J* = 5.7, 1.7 Hz, 1H, H-3a), 2.58 (t, *J* = 11.5 Hz, 1H, H$_a$-1), 2.34 – 2.26 (m, 1H, H$_a$-4), 1.85 (dd, *J* = 11.7, 5.7 Hz, 1H, H$_b$-1), 1.58 – 1.45 (m, 1H, H$_b$-4), 1.01 (s, 9H, 3xCH$_3^{tBu}$) ppm.

^{13}C-NMR, HSQC, HMBC (101 MHz, CD_3CN) δ 168.4 (CO_2), 163.9 (C-6a), 161.1 (2C, 2xCO^{NHPI}), 158.2 (C-4^{PMB}), 142.1 (C-7a), 141.2 (C-2'), 135.4 (C-11a), 134.7 (2C, C-2,6^{TBDPS}), 134.6 (2C, C-2',6'^{TBDPS}), 134.5 (2C, C5,6^{NHPI}), 134.0 (C-1^{Bn}), 132.6 (C-1^{TBDPS}), 132.5 (C-1'^{TBDPS}), 129.8 (C-1^{PMB}), 129.6 (2C, C-2,6^{Bn}), 129.1 (C-4^{TBDPS}), 129.0 (C-4'^{TBDPS}), 128.5 (2C, C-2,6^{PMB}), 127.8 (2C, C-3a,7a^{NHPI}), 127.7 (2C, C-3,5^{Bn}), 127.5 (C-9), 127.1 (2C, C-3,5^{TBDPS}), 127.0 (2C, C3',5'^{TBDPS}), 126.9 (C-4^{Bn}), 124.2 (C-3'), 123.1 (2C, C-4,7^{NHPI}), 120.7 (C-11), 119.8 (C-10), 118.3 (CN), 112.6 (2C, C-3,5^{PMB}), 108.6 (C-8), 75.6 (C-6), 70.8 (*C*H$_2^{PMB}$), 65.0 (C-4'), 62.7 (C-3a), 59.7 (C-1'), 59.1 (C-2), 54.0 (CH$_3^{PMB}$), 52.8 (*C*H$_2^{Bn}$), 52.7 (C-11b), 44.6 (C-1), 39.2 (C-5), 29.2 (C-4), 25.4 (3C, 3xCH$_3^{tBu}$), 17.9 (C$_q^{tBu}$) ppm.

IR (ATR): $\tilde{\nu}$ [cm^{-1}] = 3277, 2930, 2856, 2191, 1788, 1744, 1644, 1614, 1467, 1355.

HRMS (ESI): found 961.3976, calc for $[C_{59}H_{57}N_4O_7Si]^+$: 961.3991.

Melting range: 69.0-73.2 °C (cHex/EtOAc)

$[\boldsymbol{\alpha}]_{\boldsymbol{D}}^{\mathbf{21}} = -68.9$ (c=0.13 in $CHCl_3$)

Synthesis of S5

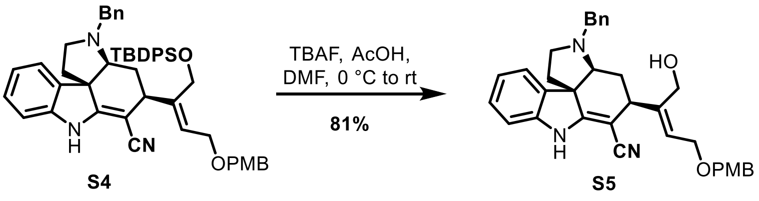

To a solution of **S4** (258 mg, 0.334 mmol, 1.0 eq.) in dry DMF (3 mL) was added AcOH (60 µL, 1.0 mmol, 3.0 eq.). The solution was cooled to 0 °C and TBAF (1 m in THF, 334 µL, 0.334 mmol, 1.0 eq.) was added dropwise. The reaction mixture was stirred at 0 °C for 30 min and then 4 h at rt. A sat. $NaHCO_3$-solution was added and the mixture was extracted three times with EtOAc. The combined organics were dried over Na_2SO_4 and the solvent was removed under reduced pressure. The crude was purified by column chromatography (silica, cHex/EtOAc 2:1). The product was obtained as a colorless solid (145 mg, 0.272 mmol) in a yield of 81%.

$\boldsymbol{R_f}$ = 0.36 (cHex/EtOAc 2:1) [Seebach]

^{1}H-NMR, COSY (600 MHz, $CDCl_3$) δ 7.40 – 7.35 (m, 6H, H-2-6^{Bn}, NH), 7.25 (d, J = 8.6 Hz, 2H, H-2,6^{PMB}), 7.14 (td, J = 7.7, 1.2 Hz, 1H, H-9), 6.86 – 6.83 (m, 2H, H-3,5^{PMB}), 6.82 (d, J = 7.8 Hz, 1H, H-8), 6.78 (td, J = 7.5, 1.0 Hz, 1H, H-10), 6.55 (d, J = 7.4 Hz, 1H, H-11), 5.91 (brs, 1H, OH), 5.81 (td, J = 6.6, 2.2 Hz, 1H, H-3'), 4.45 (s, 2H, CH_2^{PMB}), 4.31 – 4.25 (m, 1H, H_a-4'), 4.28 (d, J = 11.5 Hz, 1H, H_a-1'), 4.18 (ddd, J = 12.4, 5.9, 2.1 Hz, 1H, H_b-4'), 4.00 (d, J = 12.9 Hz, 1H, H_a-CH_2^{Bn}), 3.93 (d, J = 11.3 Hz, 1H, H_b-1'), 3.84 (d, J = 13.0 Hz, 1H, H_b-CH_2^{Bn}), 3.78 (s, 3H, CH_3^{PMB}), 3.47 (d, J = 5.9 Hz, 1H, H-3a), 3.33 (dt, J = 5.1, 2.3 Hz, 1H, H-5), 2.86 (dd, J = 9.7, 6.7 Hz, 1H, H_a-2), 2.79 (ddd, J = 12.3, 9.6, 5.2 Hz, 1H, H_b-2), 2.35 (td, J = 12.2, 6.7 Hz, 1H, H_a-1), 2.32 – 2.29 (m, 2H, H_a-4), 1.62 (dt, J = 14.8, 6.0 Hz, 1H, H_b-4), 1.54 (dd, J = 12.1, 5.1 Hz, 1H, H_b-1) ppm.

^{13}C-NMR, HSQC, HMBC (151 MHz, $CDCl_3$) δ 166.3 (C-6), 159.1 (C-4^{PMB}), 143.2 (C-2'), 142.1 (C-7a), 137.2 (C-11a), 135.1 (C-1^{Bn}), 130.6 (2C, C-3,5^{Bn}), 130.2 (C-1^{PMB}), 129.5 (2C-2,6^{PMB}), 128.8 (C-3'), 128.5 (2C, C-2,6^{Bn}), 128.3 (C-9), 127.9 (C-4^{Bn}), 121.9 (C-11), 121.0 (C-10), 119.2 (CN), 113.8 (2C, C-3,5^{PMB}), 109.4 (C-8), 76.1 (C-6a), 72.2 (CH$_2^{PMB}$), 66.4 (C-4'), 65.1 (C-3a), 58.8 (C-1'), 58.3 (CH$_2^{Bn}$), 55.3 (CH_3^{PMB}), 54.6 (C-11b), 50.3 (C-2), 41.6 (C-5), 41.4 (C-1), 30.6 (C-4) ppm.

IR (ATR): $\tilde{\nu}$ [cm^{-1}] = 3274, 2921, 2837, 2190, 1642, 1614, 1513, 1466, 1354, 1248.

ESI-MS (+ / m/z in %): 534.25 (100) $[M+H]^+$.

HRMS (ESI): found 534.2733, calc for $[C_{34}H_{36}N_3O_3]^+$: 534.2751.

Melting range: 66.4-68.8 °C ($CHCl_3$)

$[\boldsymbol{\alpha}]_{\boldsymbol{D}}^{\mathbf{21}} = -117.6$ (c=0.5 in $CHCl_3$)

Synthesis of S6

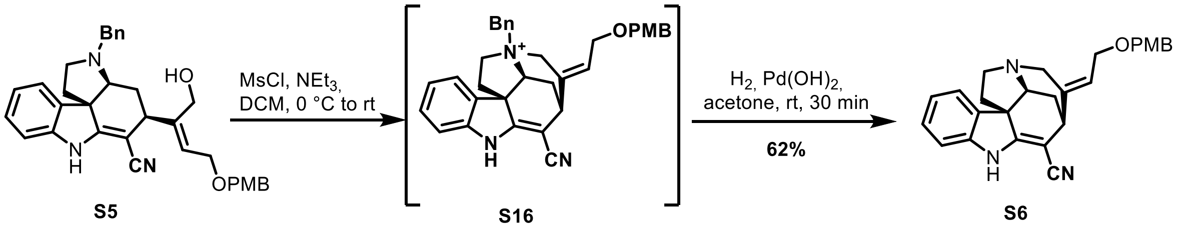

To a solution of **S5** (117 mg, 0.219 mmol, 1.0 eq.) in dry DCM (2 mL) at 0 °C was added NEt_3 (91 µL, 0.66 mmol, 3.0 eq.) and MsCl (51 µL, 0.66 mmol, 3.0 eq.). The solution was stirred at 0 °C for 1 h and afterwards at rt for 3 h. H_2O was added and the mixture was extracted three times with DCM. The combined organics were dried over Na_2SO_4 and the solvent was removed under reduced pressure. The product **S16** was obtained as a yellow foam (160 mg, quant) and was used without further purification.

R_f = 0.53 (DCM/MeOH 10:1)

ESI-MS (+ / *m/z* in %): 516.3 (100) $[M]^+$.

Crude **S16** (42 mg, ~0.069 mmol) was dissolved in acetone (4 mL) and $Pd(OH)_2$ (wet, 20% on carbon, 21 mg) was added. The solution was purged with a stream of nitrogen for 5 min. The atmosphere was changed to hydrogen and the solution was vigorously stirred under hydrogen atmosphere (double walled balloon) for 30 min. The mixture was filtered through a pad of celite, which was additionally rinsed with acetone and MeOH. The solvents were removed under reduced pressure and the residue was purified by column chromatography (silica, DCM/MeOH 100:5). The product was obtained as a colorless oil (18 mg, 0.43 mmol) in a yield of 62% over two steps.

R_f = 0.24 (DCM/MeOH 100:5) [UV]

^{1}H-NMR, COSY (400 MHz, $CDCl_3$) δ 7.26 (d, *J* = 8.6 Hz, 2H, H-2', H-6'), 7.18 (d, *J* = 7.3 Hz, 1H, H-9), 7.17 (ddd, *J* = 8.9, 7.5, 1.9 Hz, 1H, H-11), 6.96 (s, 1H, H-1), 6.92 (td, *J* = 7.5, 1.0 Hz, 1H, H-10), 6.90 – 6.86 (m, 2H, H-3', H-5'), 6.84 (dt, *J* = 7.7, 0.9 Hz, 1H, H-12), 5.81 (td, *J* = 6.7, 1.5 Hz, 1H, H-19), 4.44 (s, 2H, CH_2^{PMB}), 4.08 (q, *J* = 2.6 Hz, 1H, H-3), 4.05 – 3.92 (m, 2H, CH_2-18), 3.80 (s, 3H, CH_3^{PMB}), 3.65 (ddd, *J* = 15.9, 2.0, 1.0 Hz, 1H, H_a-21), 3.32 (s, 1H, H-15), 3.31 (dd, *J* = 15.9, 1.1 Hz, 1H, H_b-21), 3.11 (ddd, *J* = 11.5, 8.8, 6.2 Hz, 1H, H_a-5), 2.82 (ddd, *J* = 11.3, 6.5, 4.7 Hz, 1H, H_b-5), 2.30 (ddd, *J* = 12.8, 6.0, 2.4 Hz, 2H, H_a-6, H_a-14), 1.88 (ddd, *J* = 12.7, 6.2, 4.7 Hz, 1H, H_b-6), 1.39 (dt, *J* = 13.6, 2.8 Hz, 1H, H_b-14) ppm.

^{13}C-NMR, HSQC, HMBC (101 MHz, $CDCl_3$) δ 168.1 (C-2), 159.2 (C-4'), 143.6 (C-13), 140.5 (C-20), 134.9 (C-8), 130.2 (C-1'), 129.4 (2C, C-2', C-6'), 128.1 (C-11), 124.3 (C-19), 121.5 (C-10), 120.9 (C-9), 119.2 (C-17), 113.8 (2C, C-3', C-5'), 109.8 (C-12), 82.3 (C-16), 72.1 (CH_2^{PMB}), 65.0 (C-18), 59.5 (C-3), 56.9 (C-7), 55.3 (OCH_3^{PMB}), 54.2 (C-5), 47.8 (C-21), 44.4 (C-6), 36.0 (C-15), 28.9 (C-14) ppm.

IR (ATR): $\tilde{\nu}$ [cm^{-1}] = 3274, 2934, 2858, 2186, 1637, 1612, 1513, 1464, 1353, 1247.

ESI-MS (+ / *m/z* in %): 426.1 (100) $[M+H]^+$

HRMS (ESI): found 426.2174, calc for $[C_{27}H_{28}N_3O_2]^+$: 426.2176.

$[\alpha]_D^{20}$ = −255.9 (MeOH) (c=1.18)

Synthesis of S7

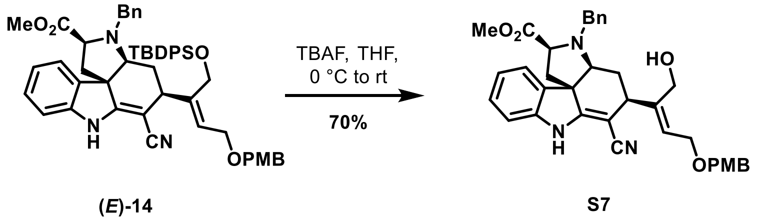

To a solution of tetracycle **(*E*)-14** (7.66 g, 9.24 mmol, 1.0 eq.) in dry THF (150 mL) was added at 0 °C a cold solution of TBAF (1 M in THF, 27.7 mL, 27.7 mmol, 3.0 eq.). The solution was warmed to rt and stirred for 2 h. H_2O was added and the mixture was extracted four times with Et_2O. The combined organics were washed with H_2O, brine and dried over Na_2SO_4. The solvent was removed under reduced pressure and the crude was purified by column chromatography (silica, cHex/EtOAc 3:2). The product was obtained as a colorless oil (3.81 g, 6.44 mmol, 70%)

$\boldsymbol{R_f}$ = 0.38 (cHex/EtOAc 1:1).

^{1}H-NMR, COSY (600 MHz, CD_3CN): δ = 7.80 (s, 1H, NH), 7.40–7.37 (m, 2H, H-2,6^{Bn}), 7.34–7.30 (m, 2H, H-3,5^{Bn}), 7.28–7.25 (m, 2H, H-11, H-4^{Bn}), 7.24–7.20 (m, 2H, H-2,6^{PMB}), 7.17 (td, 1H, *J* = 7.7 Hz, 1.2 Hz, H-9), 6.89 (td, 1H, *J* = 7.7 Hz, 1.2 Hz, H-10), 6.89 (d, 1H, *J* = 7.7 Hz, H-8), 6.85–6.81 (m, 2H, H-3,5^{PMB}), 5.79 (t, 1H, *J* = 6.5 Hz, H-3'), 4.40 (d, 1H, *J* = 11.5 Hz, H_a-CH_2^{PMB}), 4.36 (d, 1H, *J* = 11.4 Hz, H_b-CH_2^{PMB}), 4.13–4.02 (m, 6H, H_2-CH_2^{Bn}, H_2-1', H_2-4'), 3.91 (dd, 1H, *J* = 9.9 Hz, 7.1 Hz, H-2), 3.69 (s, 3H, OCH_3^{PMB}), 3.54 (s, 3H, CO_2CH_3), 3.46 (dd, 1H, *J* = 10.6 Hz, 6.3 Hz, H-5), 3.29 (dd, 1H, *J* = 10.2 Hz, 5.4 Hz, H-3a), 3.01 (t, 1H, *J* = 5.0 Hz, OH), 2.41 (dd, 1H, *J* = 12.5 Hz, 9.9 Hz, H_a-1), 2.06 (dt, 1H, *J* =14.2 Hz, 10.6 Hz, H_a-4), 1.98–1.95 (m, 1H, H_b-4), 1.93–1.91 (m, 1H, H_b-1) ppm.

^{13}C-NMR , HSQC, HMBC (151 MHz, CD_3CN): δ = 174.9 (CO_2CH_3), 164.3 (C_q-6a), 160.1 (C_q-4^{PMB}), 144.6 (C_q-7a), 144.0 (C_q-2'), 140.1 (C_q-1^{Bn}), 136.7 (C_q-11a), 131.7 (C_q-1^{PMB}), 130.4 (2C, C-2,6^{PMB}), 129.8 (2C, C-2,6^{Bn}), 129.3 (2C, C-3,5^{Bn}), 129.2 (C-9), 128.1 (C-4^{Bn}), 124.8 (C-3'), 123.6 (C-11), 121.8 (C-10), 119.1 (*C*N), 114.5 (2C, C-3,5^{PMB}), 110.5 (C-8), 78.8 (C_q-6), 72.3 (*C*H_2^{PMB}), 65.8 (C-4'), 63.9 (C-1'), 63.5 (C-3a), 63.0 (C-2), 55.7 (OCH_3^{PMB}), 54.7 (*C*H_2^{Bn}), 54.3 (C_q-11b), 52.5 (CO_2CH_3), 46.1 (C-1), 37.5 (C-5), 33.3 (C-4) ppm.

IR (ATR): $\tilde{\nu}$ [cm^{-1}] = 3292, 3000, 2951, 2859, 2194, 1733, 1644, 1613, 1513 1248, 1173.

ESI-MS (+ / *m/z* in %): 592.2 (100) [M+H]$^+$.

HR-ESI-MS: found: 592.2805 [M + H]$^+$, calc for [$C_{36}H_{38}N_3O_5$]$^+$: m/z = 592.2806.

$[\boldsymbol{\alpha}]_{\boldsymbol{D}}^{\boldsymbol{20}} = -153.7$ (c = 1.0 in MeOH).

Synthesis of S8

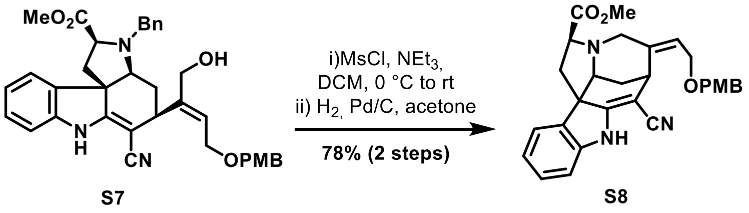

To a solution of alcohol **S7** (346 mg, 0.585 mmol, 1.0 eq.) in dry DCM (25 mL) was added NEt_3 (811 µL, 5.85 mmol, 10.0 eq.) at 0 °C. MsCl (226 µL, 2.93 mmol, 5.0 eq.) was added and the reaction mixture was stirred at 0 °C for 15 min and then at rt for 2 h. A sat solution of $NaHCO_3$ was added and the phases were separated. The organic layer was washed with water and dried over Na_2SO_4. The solvent was

removed under reduced pressure and the residue was filtered through a short plug of silica (DCM/MeOH 10:1 + 1% NEt_3) and was used without further purification.

The crude was dissolved in acetone (30 mL) and Pd (10 wt% on carbon, 10 mg) was added. The reaction was stirred for 14 h at rt under a hydrogen atmosphere (autoclave, 4 bar). The mixture was filtered through silica (EtOAc to EtOAc/MeOH 5:1) and the solvent was removed in vacuo. The residue was purified by column chromatography (silica, cHex/EtOAc 4:1 to EtOAc/MeOH 2:1). The product was obtained as a colorless oil (220 mg, 0.455 mmol) in a yield of 78% over 2 steps.

***R**$_f$* = 0.16 (cHex/EtOAc 1:4).

^{1}H-NMR, COSY (600 MHz, CD_3CN): δ = 7.99 (s, 1H, NH), 7.31 (d, 1H, *J* = 7.6 Hz, H-9), 7.30–7.27 (m, 2H, H-2', H-6'), 7.16 (t, 1H, *J* = 7.6 Hz, H-11), 6.92–6.89 (m, 2H, H-3,5^{PMB}), 6.88–6.85 (m, 2H, H-10, H-12), 5.45 (t, 1H, *J* = 6.6 Hz, H-19), 4.45 (d, 1H, *J* = 12.6 Hz, H_a-CH_2^{PMB}), 4.43 (d, 1H, *J* = 12.6 Hz, H_b-CH_2^{PMB}), 4.14 (d, 2H, *J* = 6.6 Hz, H_2-18), 4.12–4.08 (m, 2H, H-3, H-5), 3.77 (s, 3H, OCH_3^{PMB}), 3.72 (s, 3H, $CO_2C\mathit{H}_3$), 3.65 (d, 1H, *J* = 15.9 Hz, H_a-21), 3.45 (s, 1H, H-15), 3.01 (d, 1H, *J* = 15.9 Hz, H_b-21), 2.53 (t, 1H, *J* = 12.6 Hz, H_a-6), 2.39 (d, 1H, *J* = 13.6 Hz, H_a-14), 1.84 (dd, 1H, *J* = 12.6 Hz, 5.2 Hz, H_b-6), 1.41 (d, 1H, *J* = 13.6 Hz, H_b-14) ppm.

^{13}C-NMR , HSQC, HMBC (151 MHz, CD_3CN): δ = 170.9 (CO_2), 169.6 (C-2), 160.2 (C-4^{PMB}), 144.6 (C-13), 141.5 (C-20), 137.1 (C-8), 131.7 (C-1^{PMB}), 130.6 (2C, C-2,6^{PMB}), 129.1 (C-11), 124.9 (C-19), 122.1 (C-9), 121.9 (C-10), 120.1 (*C*N), 114.6 (2C, C-3,5^{PMB}), 110.5 (C-12), 81.1 (C-16), 72.6 (CH_2^{PMB}), 67.4 (C-5), 67.0 (C-18), 63.0 (C-3), 56.8 (C-7), 55.8 ($O\mathit{C}H_3^{PMB}$), 52.7 ($CO_2\mathit{C}H_3$), 50.7 (C-21), 48.7 (C-6), 34.1 (C-15), 30.8 (C-14) ppm.

IR (ATR): $\tilde{\nu}$ [cm^{-1}] = 3292, 2952, 2854, 2185, 1739, 1638, 1613, 1513, 1465, 1247, 752.

ESI-MS (+ / *m/z* in %): 484.2 (100) $[M+H]^+$.

HR-ESI-MS: found: 484.2228 $[M + H]^+$, calc for $[C_{29}H_{30}N_3O_4]^+$: 484.2231.

$[\alpha]_D^{20} = -75.7$ (c = 0.01 in MeOH).

Synthesis of S9

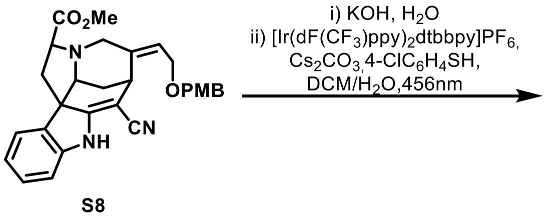

To a solution of tetracycle **S8** (4.1 mg, 8.5 µmol, 1.0 eq.) in MeOH (0.3 mL) was added KOH (10% in H_2O, 300 µL) and the solution was stirred at rt for 30 min. The solution was acidified with 1 M HCl to pH = 2 and the mixture was extracted three times with EtOAc. The combined organics were washed with H2O and dried over Na_2SO_4. The solvent was removed in vacuo.

The crude acid (8.5 µmol, 1.0 eq.), Cs_2CO_3 (4.2 mg, 13 µmol, 1.5 eq.) and $[Ir(dF(CF_3)ppy)_2(dtbbpy)]PF_6$ (0.2 mg, 0.2 µmol, 2mol%) were dissolved in degassed $MeCN/H_2O$ (9:1, 0.25 mL). tBuSH (9 µL, 85 µmol, 10 eq.) were added and the reaction mixture was irradiated with Kessil LED-Spotlight (456 nm, 25%

intensity) for 2 h. The mixture was diluted with H_2O and was extracted three times with EtOAc. The extracts were washed with brine and dried over Na_2SO_4. The solvent was removed under reduced pressure and the residue was purified by preparative HPLC (MN C18-HTec, isocratic MeCN/H_2O(+0.1% FA) 45:55, tR = 10.0 min. The product was obtained as a colorless foam (2.0 mg, 4.8 µmol, 56%).

^{1}H-NMR, COSY (600 MHz, CD3CN): δ = 9.78 (s, 1H, NH), 7.41 (d, 1H, *J* = 7.3 Hz, H-9), 7.26–7.22 (m, 2H, H-2', H-6'), 7.12 (td, 1H, *J* = 7.6 Hz, 1.1 Hz, H-11), 6.90–6.87 (m, 2H, H-3', H-5'), 6.86–6.82 (m, 2H, H-10, H-12), 5.54 (s, 1H, H-21), 4.42–4.36 (m, 2H, H_2-1'a), 4.03 (dd, 1H, *J* = 4.9 Hz, 2.9 Hz, H-3), 3.73 (s, 3H, 4'-OCH_3), 3.58–3.48 (m, 2H, H_2-18), 3.10 (td, 1H, *J* = 12.2 Hz, 4.6 Hz, H_a-5), 3.00 (dd, 1H, *J* = 12.2 Hz, 6.6 Hz, H_b-5), 2.75 (dd, 1H, *J* = 4.9 Hz, 2.7 Hz, H-15), 2.30–2.19 (m, 2H, H_2-20), 2.05 (td, 1H, *J* = 12.2 Hz, 6.6 Hz, H_a-6), 1.91 (dt, 1H, *J* = 12.7 Hz, 2.7 Hz, H_a-14), 1.53 (dd, 1H, *J* = 12.2 Hz, 4.6 Hz, H_b-6), 1.33 (d, 1H, *J* = 12.7 Hz, 2.9 Hz, H_b-14) ppm.

^{13}C-NMR , HSQC, HMBC (151 MHz, CD3CN): δ = 168.9 (Cq-2), 158.6 (Cq-4'), 144.1 (Cq-13), 135.9 (Cq-8), 132.6 (C-21), 130.6 (Cq-1'), 129.2 (2C, C-2', C-6'), 127.7 (C-11), 120.7 (C-9), 120.4 (C-10), 119.9 (CqN), 118.0 (Cq-20), 113.6 (2C, C-3', C-5'), 109.5 (C-12), 79.9 (Cq-16), 71.5 (C-1'a), 68.4 (C-18), 58.5 (C-3), 57.8 (Cq-7), 55.1 (4'-OCH3), 52.3 (C-5), 46.9 (C-6), 34.6 (C-19), 32.2 (C-15), 30.2 (C-14) ppm.

IR (ATR): $\tilde{\nu}$ [cm^{-1}] = 3400, 2925, 2854, 2188, 1630, 1611, 1513, 1465, 1247, 1113, 1093

ESI-MS (+ / *m/z* in %): 426.2 (100) $[M+H]^+$.

HR-ESI-MS: found 426.2179; calc. for $[C_{27}H_{28}N_3O_2]^+$: 426.2176,

$[\boldsymbol{\alpha}]_{\boldsymbol{D}}^{\mathbf{20}}$ = -53.3 (c = 0.015 in MeOH)

Synthesis of S10

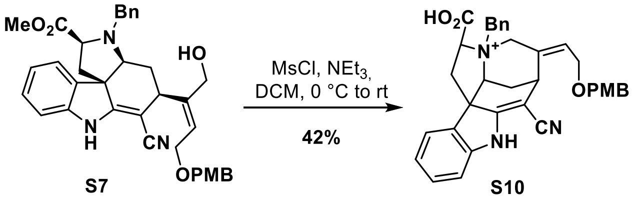

To a solution of alcohol **S7** (3.81 g, 6.44 mmol, 1.0 eq.) in dry DCM was added at 0 °C NEt_3 (8.93 mL, 64.4 mmol, 10.0 eq.) and MsCl (2.49 mL, 32.2 mmol, 5.0 eq.). The resulting solution was stirred at 0 °C for 15 min and for 2 h at rt. A solution of $NaHCO_3$ was added and the phases were separated. The organic layer was washed with water and dried over Na_2SO_4. The solvent was removed and the crude was purified by column chromatography (silica, DCM/MeOH 10:1 + 1% NEt_3) The product was obtained as a colorless oil (1.53 g, 2.73 mmol, 42%).

Note: during reaction only the methyl ester (m/z = 574.3 $[M]^+$) was observed. After extraction only the hydrolysis product (m/z = 560.3 $[M]^+$) was observed.

$\boldsymbol{R_f}$ = 0.42 (DCM/MeOH 10:1 + 1% NEt3).

^{1}H-NMR, COSY (600 MHz, DMSO-d6): δ = 10.38 (s, 1H, NH), 7.83 (d, 2H, J = 7.4 Hz, H-2', H-6'), 7.69–7.65 (m, 1H, H-4'), 7.61 (t, 2H, J = 7.4 Hz, H-3', H-5'), 7.30–7.26 (m, 2H, H-2'', H-6''), 7.11 (td, 1H, J = 7.7

Hz, 1.2 Hz, H-11), 6.92–6.89 (m, 2H, H-3'', H-5''), 6.82 (d, 1H, J = 7.7 Hz, H-12), 6.57 (td, 1H, J = 7.7 Hz, 0.9 Hz, H-10), 5.80–5.78 (m, 1H, H-19), 5.33 (d, 1H, J = 12.6 Hz, Ha-1'a), 5.12 (d, 1H, J = 15.0 Hz, Ha-21), 4.62 (s, 1H, H-3), 4.44 (s, 2H, H2-1''a), 4.21–4.13 (m, 3H, Hb-21, H2-18), 4.12–4.08 (m, 2H, H-3, H-5), 4.03 (dd, 1H, J =14.1 Hz, 6.3 Hz, H-5), 3.74 (s, 3H, 4''-OCH3), 3.64 (s, 1H, H-15), 2.73 (dd, 1H, J = 14.8 Hz, 2.7 Hz, Ha-14), 2.64 (t, 1H, J = 14.1 Hz, Ha-6), 1.84 (dd, 1H, J = 14.1 Hz, 6.3 Hz, Hb-6), 1.65 (d, 1H, J = 14.8 Hz, Hb-14) ppm.

^{13}C-NMR, HSQC, HMBC (151 MHz, DMSO-d6): δ = 165.2 (Cq-2), 163.8 (CO2CH3), 158.8 (Cq-4''), 143.2 (Cq-13), 134.4 (Cq-8), 134.3 (Cq-20), 134.0 (2C, C-2', C-6'), 130.7 (C-4'), 130.4 (C-19), 130.0 (Cq-1''), 129.6 (2C, C-2'', C-6''), 129.2 (2C, C-3', C-5'), 129.0 (C-11), 128.5 (Cq-1'), 120.5 (C-10), 119.7 (C-9), 119.0 (CqN), 113.7 (2C, C-3'', C-5''), 110.2 (C-12), 78.2 (Cq-16), 71.6 (C-1''a), 70.0 (C-5), 67.4 (C-3), 66.1 (C-18), 64.7 (C-1'a), 59.6 (C-21), 55.1 (4''-OCH3), 51.7 (Cq-7), 46.7 (C-6), 31.4 (C-15), 27.3 (C-14) ppm.

IR (ATR): $\tilde{\nu}$ [cm^{-1}] = 3351, 3003, 2954, 2837, 2190, 1638, 1613, 1513, 1467, 1355, 1246.

ESI-MS (+ / *m/z* in %): 560.3 (100) [M-H]$^+$.

HRMS (ESI): found 560.2555 [M + H]$^+$ (calc. for [$C_{35}H_{34}N_3O_4$]+ : 560.2544).

$[\alpha]_D^{22}$ = −147.5 (c=0.16 in MeOH)

Synthesis of S11 and S12

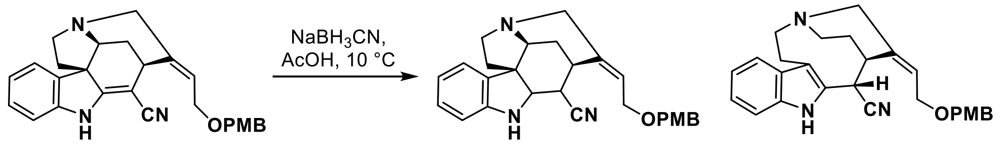

To a solution of **17** (33 mg, 0.077 mmol, 1.0 eq.) in AcOH (1.9 mL) at 15 °C was added $NaBH_3CN$ (72 mg, 1.15 mmol, 1.0 eq.) in small portions. After the addition was finished the reaction was stirred for further 4 h. The solution was added to a solution of $NaHCO_3$ and the mixture was extracted three times with DCM. The combined organics were dried over Na_2SO_4 and the solvent was removed under reduced pressure. The crude was purified by column chromatography (silica, DCM/MeOH(+10% NH_4OH_{aq}) 95:5). The compounds **S11** was isolated as a yellowish solid (8.5 mg, 0.020 mmol, 26%) alongside compound **S12** as a brownish solid (9.8 mg, 0.023 mmol, 30%)

Analytical data for **S11**

R$_f$ = 0.10 (DCM/MeOH (+10%NH_4OH_{aq}) 95:5)

^{1}H-NMR, COSY (600 MHz, $CDCl_3$) δ 7.32 – 7.27 (m, 2H, H-2',6'), 7.12 (td, *J* = 7.6, 1.2 Hz, 1H, H-11), 7.03 (dd, *J* = 7.4, 1.2 Hz, 1H, H-9), 6.97 – 6.91 (m, 2H, H-3',5'), 6.81 (td, *J* = 7.4, 1.0 Hz, 1H, H-10), 6.72 (d, *J* = 7.8 Hz, 1H, H-12), 5.63 (t, *J* = 7.3 Hz, 1H, H-19), 4.53 (d, *J* = 11.4 Hz, 1H, H_a-CH_2^{PMB}), 4.43 (d, *J* = 11.5 Hz, 1H, H_b-CH_2^{PMB}), 4.05 – 3.97 (m, 1H, H_a-18), 3.95 – 3.89 (m, 2H, 1, H_b-18), 3.84 (s, 1H, H-3), 3.83 (s, 3H, CH_3^{PMB}), 3.76 (d, *J* = 15.8 Hz, 1H, H_a-21), 3.54 (d, *J* = 7.1 Hz, 1H, H-2), 3.27 (t, *J* = 9.2 Hz, 1H, H_b-5), 3.15 (dd, *J* = 7.0, 2.5 Hz, 1H, H-16), 3.08 (s, 1H, H-15), 2.91 (d, *J* = 15.9 Hz, 1H, H_b-21), 2.74 (ddd, *J* = 11.6, 10.3, 6.4 Hz, 1H, H_a-5), 2.12 – 1.98 (m, 3H, H_a-6, H_2-14), 1.89 – 1.72 (m, 1H, H_b-6) ppm.

^{13}C-NMR, HSQC, HMBC (151 MHz, $CDCl_3$) δ 159.5 (C-4’), 149.5 (C-13), 138.9 (br, C-20), 129.9 (C-8), 129.8 (2C, C-2’,6’), 129.7 (C-1’), 128.8 (C-11), 125.2 (br, C-19), 121.8 (C-9), 119.9 (C-17), 119.8 (C-10), 114.0 (2C, C-3’,5’), 110.0 (C-12), 72.6 (CH_2^{PMB}), 64.7 (C-18), 58.9 (C-2), 58.3 (C-3), 55.3 (CH_3^{PMB}), 53.8 (C-21), 52.9 (C-7), 51.8 (C-5), 38.7 (C-6), 37.7 (C-16), 31.0 (C-15), 21.2 (C-14) ppm.

IR (ATR): $\tilde{\nu}$ [cm^{-1}] = 3364, 2930, 2855, 2243, 1610, 1513, 1466, 1248, 1032, 736.

ESI-MS (+ / *m/z* in %): 428.2 (100) $[M+H]^+$

HRMS (ESI): found 428.2327, calc. for $[C_{27}H_{30}N_3O_2]^+$: 428.2333.

Optical Rotation $[\alpha]_D^{21} = -87.9$ (c=0.33, $CHCl_3$)

Analytical data for **S12**

R$_f$ = 0.20 (DCM/MeOH (+10% NH_4OH_{aq}) 95:5)

^{1}H-NMR, COSY (600 MHz, $CDCl_3$) δ 8.27 (s, 1H, NH), 7.52 – 7.47 (m, 1H, H-12), 7.36 – 7.30 (m, 3H, H-2,6^{PMB}, H-9), 7.21 (ddd, *J* = 8.1, 7.0, 1.1 Hz, 1H, H-10), 7.13 (ddd, *J* = 8.0, 7.0, 1.0 Hz, 1H, H-11), 6.91 (d, *J* = 8.6 Hz, 2H, H-3,5^{PMB}), 5.66 (s, 1H, H-2‘), 4.64 – 4.60 (d, *J* = 2.2 Hz 1H, H-7), 4.57 (d, *J* = 11.3 Hz, 1H, H_a-CH_2^{PMB}), 4.54 (d, *J* = 11.2 Hz, 1H, H_b-CH_2^{PMB}), 4.17 – 4.11 (m, 1H, H_a-1‘), 4.01 (dd, *J* = 11.1, 7.3 Hz, 1H, H_b-1‘), 3.80 (s, 3H, CH_3), 3.70 (d, *J* = 16.9 Hz, 1H, H_a-4), 3.68 – 3.63 (m, 1H, H-6), 3.17 (br, 1H, H_b-4), 3.15 – 3.07 (m, 2H, H_2-2), 2.99 (ddd, *J* = 12.8, 8.4, 3.3 Hz, 2H, H_a-1, H_a-14), 2.88 (br, 1H, H_b-1), 1.87 – 1.74 (m, 1H, H_a-15) ppm.

^{13}C-NMR, HSQC, HMBC (151 MHz, $CDCl_3$) δ 159.5 (C-4^{PMB}), 145.8 (C-5), 135.4 (C-8a), 129.8 (2C, C-2,6^{PMB}), 129.6 (C-1^{PMB}), 127.7 (C-12a), 126.0 (br, C-7a), 122.5 (C-10), 120.9 (br, C-2’), 120.0 (br, CN), 119.9 (C-11), 118.5 (C-12), 114.1 (2C, C-3,5^{PMB}), 113.5 (C-12b), 111.2 (C-9), 72.9 (CH_2^{PMB}), 65.4 (C-1‘), 56.8 (C-2), 55.3 (CH_3), 53.4 (C-4), 44.4 (C-14), 37.3 (br, C-6), 36.7 (C-7), 25.1 (C-1) ppm.

(Note NMR: H_b-15 and C-15 could not be unambiguously assigned due to signal quality.)

IR (ATR): $\tilde{\nu}$ [cm^{-1}] = 3352, 2928, 2852, 2244, 1611, 1513, 1460, 1302, 1247, 1034.

ESI-MS (+ / *m/z* in %): 428.2 (100) $[M+H]^+$

HRMS (ESI): found 428.2327, calc. for $[C_{27}H_{30}N_3O_2]^+$: 428.2333

Synthesis of **S15**

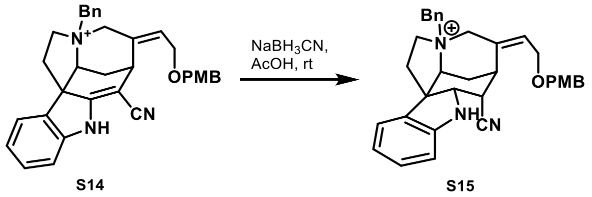

To a solution of **S14** (25 mg, 0.041 mmol, 1.0 eq.) in AcOH (1.5 mL) at 15 °C was added $NaBH_3CN$ (50 mg, 0.80 mmol, 19 eq.) portion-wise over 2 h. The reaction mixture was stirred additional 2 h and was quenched with an aqueous solution of $NaHCO_3$. The mixture was extracted three times with DCM and the combined organics were dried over Na_2SO_4 and the solvent was removed under reduced

pressure. The crude was purified by column chromatography (silica, DCM/MeOH(+10%NH_4OH_{aq}) 9:1) and the product **S15** was obtained as colourless solid (7.5 mg, 0.012 mmol) in 30% yield.

R_f = 0.29 (DCM/MeOH(+10% NH_4OH_{aq}) 9:1)

^{1}H-NMR, COSY (600 MHz, CD_3OD) δ 7.82 – 7.75 (m, 2H, H-2,6^{Bn}), 7.64 – 7.58 (m, 3H, H-3,4,5^{Bn}), 7.35 – 7.29 (m, 2H, H-2',6'), 7.14 (d, *J* = 7.5 Hz, 1H, H-9), 7.11 (dd, *J* = 7.7, 1.1 Hz, 1H, H-11), 6.97 – 6.91 (m, 2H, H-3',5'), 6.77 (td, *J* = 7.5, 1.0 Hz, 1H, H-10), 6.69 (d, *J* = 7.8 Hz, 1H, H-12), 6.12 – 6.06 (m, 1H, H-19), 4.80 (d, *J* = 12.8 Hz, 1H, H_a-CH_2^{Bn}), 4.75 (d, *J* = 12.8 Hz, 1H, H_b-CH_2^{Bn}), 4.65 (d, *J* = 3.4 Hz, 1H, H-3), 4.53 (d, *J* = 11.4 Hz, 1H, H_a-CH_2^{PMB}), 4.50 (d, *J* = 11.3 Hz, 1H, H_b-CH_2^{PMB}), 4.32 (d, *J* = 14.2 Hz, 1H, H_a-21), 4.24 – 4.13 (m, 2H, H_2-18), 4.11 – 4.03 (m, 1H, H_a-5), 3.78 (s, 3H, CH_3^{PMB}), 3.81 – 3.75 (m, 2H, H-2, H_b-21), 3.66 (ddd, *J* = 7.7, 3.6, 1.7 Hz, 1H, H-16), 3.52 (td, *J* = 12.9, 6.1 Hz, 1H, H_b-5), 3.33 (s, 1H, H-15), 2.46 – 2.39 (m, 1H, H_a-14), 2.17 (s, 2H, H_a-6, H_b-14), 1.58 (td, *J* = 13.2, 7.5 Hz, 1H, H_b-6) ppm.

^{13}C-NMR, HSQC, HMBC (151 MHz, CD_3OD) δ 158.9 (C-4'), 149.3 (C-13), 133.9 (C-19), 132.2 (2C, C-2,3^{Bn}), 130.1 (C-4^{Bn}), 128.9 (C-11), 128.9 (C-1'), 128.8 (2C, C-2',6'), 128.6 (2C, C-3,5^{Bn}), 127.6 (C-20), 127.3 (C-1^{Bn}), 125.3 (C-8), 120.4 (C-9), 118.0 (C-10), 117.7 (C-17), 112.8 (2C, C-3',5'), 109.2 (C-12), 71.8 (CH_2^{PMB}), 69.2 (C-3), 67.2 (CH_2^{Bn}), 64.3 (C-18), 61.9 (C-21), 58.2 (C-5), 56.9 (C-2), 53.6 (CH_3^{PMB}), 52.7 (C-7), 36.0 (C-16), 34.7 (C-6), 28.8 (C-15), 18.6 (C-14) ppm.

IR (ATR): $\tilde{\nu}$ [cm^{-1}] = 3128, 3042, 2922, 2851, 2326, 2191, 2025, 1674, 1610, 1406.

ESI-MS (+ / *m/z* in %): 518.2 (100) $[M]^+$

HRMS (ESI): found 518.2790, calc. for $[C_{34}H_{36}N_3O_2]^+$: 518.2802.

$[\alpha]_D^{21}$ = +14.1 (c=0.75, MeOH)

References

[1] G. Lippke, H. Thaler, “Die spezifische Drehung des Sorbits und des Sorbit-Molybdat-Komplexes” *Starch - Stärke* **1970**, *22*, 344–351.

[2] R. B. Woodward, M. P. Cava, W. D. Ollis, A. Hunger, H. U. Daeniker, K. Schenker, “The total synthesis of strychnine” *J. Am. Chem. Soc.* **1954**, *76*, 4749–4751.

[3] R. B. Woodward, M. P. Cava, W. D. Ollis, A. Hunger, H. U. Daeniker, K. Schenker, “The total synthesis of strychnine” *Tetrahedron* **1963**, *19*, 247–288.

[4] P. Magnus, M. Giles, R. Bonnert, C. S. Kim, L. McQuire, A. Merritt, N. Vicker, “Synthesis of Strychnine via the Wieland-Gumlich Aldehyde” *J. Am. Chem. Soc.* **1992**, *114*, 4403–4405.

[5] Stork G. in *Ischia Porto Advanced School of Organic Chemistry, September*, Italien, **1992**.

[6] P. Magnus, M. Giles, R. Bonnert, G. Johnson, L. McQuire, M. Deluca, A. Merritt, C. S. Kim, N. Vicker, “Synthesis of Strychnine and the Wieland-Gumlich Aldehyde” *J. Am. Chem. Soc.* **1993**, *115*, 8116–8129.

[7] S. D. Knight, L. E. Overman, G. Pairaudeau, “Asymmetric Total Syntheses of (—)- and (+)-Strychnine and the Wieland—Gumlich Aldehyde” *J. Am. Chem. Soc.* **1995**, *117*, 5776–5788.

[8] S. D. Knight, L. E. Overman, G. Pairaudeau, “Enantioselective Total Synthesis of (−)-Strychnine” *J. Am. Chem. Soc.* **1993**, *115*, 9293–9294.

[9] M. E. Kuehne, F. Xu, “Total Synthesis of Strychnan and Aspidospermatan Alkaloids. 3. The Total Synthesis of (±)-Strychnine” *J. Org. Chem.* **1993**, *58*, 7490–7497.

[10] V. H. Rawal, S. Iwasa, “A Short, Stereocontrolled Synthesis of Strychnine” *J. Org. Chem.* **1994**, *59*, 2685–2686.

[11] M. E. Kuehne, F. Xu, “Syntheses of strychnan- and aspidospermatan-type alkaloids. 10. An enantioselective synthesis of (-)-strychnine through the Wieland-Gumlich aldehyde” *J. Org. Chem.* **1998**, *63*, 9427–9433.

[12] D. Sole, J. Bonjoch, S. García-Rubio, E. Peidro, J. Bosch, “Enantioselective Total Synthesis of Wieland-Gumlich Aldehyde and (−)-Strychnine.” *Chem. - Eur. J.* **2000**, *6*, 655–665.

[13] D. Solé, J. Bonjoch, S. García-Rubio, E. Peidró, J. Bosch, “Total Synthesis of (−)-Strychnine via the Wieland–Gumlich Aldehyde” *Angew. Chem. Int. Ed.* **1999**, *38*, 395–397.

[14] M. J. Eichberg, R. L. Dorta, K. Lamottke, K. P. C. Vollhardt, “The formal total synthesis of (±)-strychnine via a cobalt-mediated [2 + 2 + 2]cycloaddition” *Org. Lett.* **2000**, *2*, 2479–2481.

[15] M. J. Eichberg, R. L. Dorta, D. B. Grotjahn, K. Lamottke, M. Schmidt, K. P. C. Vollhardt, “Approaches to the synthesis of (±)-strychnine via the cobalt-mediated [2 + 2 + 2] cycloaddition: Rapid assembly of a classic framework” *J. Am. Chem. Soc.* **2001**, *123*, 9324–9337.

[16] M. Ito, C. W. Clark, M. Mortimore, J. B. Goh, S. F. Martin, “Biogenetically inspired approach to the Strychnos alkaloids. Concise syntheses of (±)-akuammicine and (±)-strychnine” *J. Am. Chem. Soc.* **2001**, *123*, 8003–8010.

[17] M. Nakanishi, M. Mori, "Total Synthesis of (−)-Strychnine" *Angew. Chem. Int. Ed.* **2002**, *41*, 1934–1936.

[18] M. Mori, M. Nakanishi, D. Kajishima, Y. Sato, "A novel and general synthetic pathway to strychnos indole alkaloids: Total syntheses of (-)-tubifoline, (-)-dehydrotubifoline, and (-)-strychnine using palladium-catalyzed asymmetric allylic substitution" *J. Am. Chem. Soc.* **2003**, *125*, 9801–9807.

[19] G. J. Bodwell, J. Li, "A Concise Formal Total Synthesis of (AE)-Strychnine by Using a Transannular Inverse-Electron-Demand Diels±Alder Reaction of a [3](1,3)Indolo[3](3,6)pyridazinophane**" *Angew. Chem. Int. Ed.* **2002**, *41*, 3261–3262.

[20] T. Ohshima, Y. Xu, R. Takita, S. Shimizu, D. Zhong, M. Shibasaki, "Enantioselective total synthesis of (-)-strychnine using the catalytic asymmetric Michael reaction and tandem cyclization" *J. Am. Chem. Soc.* **2002**, *124*, 14546–14547.

[21] Y. Kaburagi, H. Tokuyama, T. Fukuyama, "Total synthesis of (-)-strychnine" *J. Am. Chem. Soc.* **2004**, *126*, 10246–10247.

[22] H. Zhang, J. Boonsombat, A. Padwa, "Total synthesis of (±)-strychnine via a [4 + 2]-cycloaddition/ rearrangement cascade" *Org. Lett.* **2007**, *9*, 279–282.

[23] G. Sirasani, T. Paul, W. Dougherty, S. Kassel, R. B. Andrade, "Concise total syntheses of (±)-Strychnine and (±)-Akuammicine" *J. Org. Chem.* **2010**, *75*, 3529–3532.

[24] C. Beemelmanns, H. U. Reissig, "A Short Formal Total Synthesis of Strychnine with a Samarium Diiodide Induced Cascade Reaction as the Key Step" *Angew. Chem. Int. Ed.* **2010**, *49*, 8021–8025.

[25] D. B. C. Martin, C. D. Vanderwal, "A synthesis of strychnine by a longest linear sequence of six steps" *Chem. Sci.* **2011**, *2*, 649–651.

[26] S. B. Jones, B. Simmons, A. Mastracchio, D. W. C. MacMillan, "Collective synthesis of natural products by means of organocascade catalysis" *Nature* **2011**, *475*, 183–188.

[27] G. Jacquemot, G. Maertens, S. Canesi, "Isostrychnine synthesis mediated by hypervalent iodine reagent" *Chem. - Eur. J.* **2015**, *21*, 7713–7715.

[28] L. W. Feng, H. Ren, H. Xiong, P. Wang, L. Wang, Y. Tang, "Reaction of Donor-Acceptor Cyclobutanes with Indoles: A General Protocol for the Formal Total Synthesis of (±)-Strychnine and the Total Synthesis of (±)-Akuammicine" *Angew. Chem. Int. Ed.* **2017**, *56*, 3055–3058.

[29] G. S. Lee, G. Namkoong, J. Park, D. Y. K. Chen, "Total Synthesis of Strychnine" *Chem. - Eur. J.* **2017**, *23*, 16189–16193.

[30] L. He, X. Wang, X. Wu, Z. Meng, X. Peng, X. Y. Liu, Y. Qin, "Asymmetric Total Synthesis of (+)-Strychnine" *Org. Lett.* **2019**, *21*, 252–255.

[31] L. S. Hutchings-Goetz, C. Yang, J. W. B. Fyfe, T. N. Snaddon, "Enantioselective Syntheses of Strychnos and Chelidonium Alkaloids through Regio- and Stereocontrolled Cooperative Catalysis" *Angew. Chem. Int. Ed.* **2020**, *59*, 17556–17564.

[32] P. Wang, J. Chen, W. He, J. Song, H. Song, H. Wei, W. Xie, "An Asymmetric Synthesis of (+)-Isostrychnine Based on Catalytic Asymmetric Tandem Double Michael Addition" *Org. Lett.* **2021**, *23*, 5476–5479.

[33] X. Liu, M. Lou, S. Bai, G. Sun, X. Qi, “Asymmetric Total Syntheses of Strychnos Alkaloids via Selective Fischer Indolization” *J. Org. Chem.* **2022**, *87*, 5199–5212.

[34] L. P. Zhao, S. Y. Zhang, H. K. Liu, Y. J. Cheng, Z. P. Liu, L. Wang, Y. Tang, “Insights into Stereoselectivity Switch in Michael Addition-Initiated Tandem Mannich Cyclizations and Their Extension from Enamines to Vinyl Ethers” *J. Am. Chem. Soc.* **2023**, *145*, 15553–15564.

[35] W. Zhou, S. Xi, H. Chen, D. Jiang, J. Yang, S. Liu, L. He, H. Qiu, Y. Lan, M. Zhang, “A bridged backbone strategy enables collective synthesis of strychnan alkaloids” *Nat. Chem.* **2023**, *15*, 1074–1082.

[36] G. I. Tesser, I. C. Balvert-Geers, “The methylsulfonylethyloxycarbonyl group, a new and versatile amino protective function” *Int. J. Pept. Protein Res.* **1975**, *7*, 295–305.

[37] C. Cassani, G. Bergonzini, C. J. Wallentin, “Photocatalytic decarboxylative reduction of carboxylic acids and its application in asymmetric synthesis” *Org Lett* **2014**, *16*, 4228–4231.

[38] Y. Lu, J. G. West, “Chemoselective Decarboxylative Protonation Enabled by Cooperative Earth-Abundant Element Catalysis” *Angew. Chem. Int. Ed.* **2023**, *62*, e202213055.

[39] T. S. Mayer, T. Taeufer, S. Brandt, J. Rabeah, J. Pospech, “Photomediated Hydro- and Deuterodecarboxylation of Pharmaceutically Relevant and Natural Aliphatic Carboxylic Acids” *J. Org. Chem.* **2023**, *88*, 6347–6353.

[40] T. Itou, Y. Yoshimi, K. Nishikawa, T. Morita, Y. Okada, N. Ichinose, M. Hatanaka, “A mild deuterium exchange reaction of free carboxylic acids by photochemical decarboxylation” *Chem. Commun.* **2010**, *46*, 6177.

[41] Y. Yoshimi, “Photoinduced electron transfer-promoted decarboxylative radical reactions of aliphatic carboxylic acids by organic photoredox system” *J. Photochem. Photobiol., A* **2017**, *342*, 116–130.

[42] J. D. Griffin, M. A. Zeller, D. A. Nicewicz, “Hydrodecarboxylation of Carboxylic and Malonic Acid Derivatives via Organic Photoredox Catalysis: Substrate Scope and Mechanistic Insight” *J. Am. Chem. Soc.* **2015**, *137*, 11340–11348.

[43] C.-Q. Deng, Y. Xu, J.-H. Luo, G.-Z. Wang, J. Deng, Y. Fu, “Chemoselective direct deuterodecarboxylation of free aliphatic carboxylic acids enabled by deuteron-coupled electron transfer” *Chem Catal.* **2024**, *4*, 100899.

[44] N. Li, Y. Ning, X. Wu, J. Xie, W. Li, C. Zhu, “A highly selective decarboxylative deuteration of carboxylic acids” *Chem. Sci.* **2021**, *12*, 5505–5510.

[45] D. H. R. Barton, D. Crich, W. B. Motherwell, “New and improved methods for the radical decarboxylation of acids” *J. Chem. Soc., Chem. Commun.* **1983**, 939.

[46] H. Yamamoto, K. Yamaoka, A. Shinohara, K. Shibata, K. Takao, A. Ogura, “Red-light-mediated Barton decarboxylation reaction and one-pot wavelength-selective transformations” *Chem. Sci.* **2023**, *14*, 11243–11250.

[47] K. Okada, K. Okubo, N. Morita, M. Oda, “Reductive decarboxylation of N-(acyloxy)phthalimides via redox-initiated radical chain mechanism” *Tetrahedron Lett.* **1992**, *33*, 7377–7380.

[48] Z. Li, K.-F. Wang, X. Zhao, H. Ti, X.-G. Liu, H. Wang, "Manganese-mediated reductive functionalization of activated aliphatic acids and primary amines" *Nat Commun* **2020**, *11*, 5036.

[49] E. de Pedro Beato, D. Spinnato, W. Zhou, P. Melchiorre, "A General Organocatalytic System for Electron Donor–Acceptor Complex Photoactivation and Its Use in Radical Processes" *J. Am. Chem. Soc.* **2021**, *143*, 12304–12314.

[50] J. Zhang, J. Yang, L. Guo, X. Duan, "Visible-Light-Mediated Dual Decarboxylative Coupling of Redox-Active Esters with α,β-Unsaturated Carboxylic Acids" *Chem. - Eur. J.* **2017**, *23*, 10259–10263.

[51] T. Qin, L. R. Malins, J. T. Edwards, R. R. Merchant, A. J. E. Novak, J. Z. Zhong, R. B. Mills, M. Yan, C. Yuan, M. D. Eastgate, P. S. Baran, "Nickel-Catalyzed Barton Decarboxylation and Giese Reactions: A Practical Take on Classic Transforms" *Angew. Chem. Int. Ed.* **2017**, *56*, 260–265.

[52] R. L. Parsons, J. D. Berk, M. E. Kuehne, "Total synthesis of strychnan- and aspidospermatan-type alkaloids. 2. Generation of 15-(3-furanyl) ABCE tetracyclic intermediates" *J. Org. Chem.* **1993**, *58*, 7482–7489.

[53] M. E. Kuehne, F. Xu, "Syntheses of Strychnan- and Aspidospermatan-Type Alkaloids. 9. [1] The Enantioselective Generation of Tetracyclic ABCE Intermediates by a Tandem Condensation, [3,3]-Sigmatropic Rearrangement, and Cyclization Sequence" *J. Org. Chem.* **1997**, *62*, 7950–7960.

[54] M. E. Kuehne, C. S. Brook, D. A. Frasier, F. Xu, "Syntheses of Strychnos- and Aspidospermatan-Type Alkaloids. 5. Total Syntheses of (.+-.)-Echitamidine and 20-epi- and 19-epi-20-epi-Echitamidine" *J. Org. Chem.* **1994**, *59*, 5977–5982.

[55] M. E. Kuehne, P. A. Matson, W. G. Bornmann, "Enantioselective syntheses of vinblastine, leurosidine, vincovaline and 20'-epi-vincovaline" *J. Org. Chem.* **1991**, *56*, 513–528.

[56] M. Koura, Y. Yamaguchi, S. Kurobuchi, H. Sumida, Y. Watanabe, T. Enomoto, T. Matsuda, A. Okuda, T. Koshizawa, Y. Matsumoto, K. Shibuya, "Discovery of a 2-hydroxyacetophenone derivative as an outstanding linker to enhance potency and β-selectivity of liver X receptor agonist" *Bioorg. Med. Chem.* **2016**, *24*, 3436–3446.

[57] B. M. Trost, R. C. Livingston, "An atom-economic and selective ruthenium-catalyzed redox isomerization of propargylic alcohols. An efficient strategy for the synthesis of leukotrienes" *J. Am. Chem. Soc.* **2008**, *130*, 11970–11978.

[58] L. E. Overman, M. D. Rosen, "Terminating catalytic asymmetric Heck cyclizations by stereoselective intramolecular capture of η3-allylpalladium intermediates: total synthesis of (−)-spirotryprostatin B and three stereoisomers" *Tetrahedron* **2010**, *66*, 6514–6525.

[59] T. Jeffery, "Palladium-catalysed vinylation of organic halides under solid–liquid phase transfer conditions" *J. Chem. Soc., Chem. Commun.* **1984**, 1287–1289.

[60] M. E. Kuehne, F. Xu, "Syntheses of Strychnan- and Aspidospermatan-Type Alkaloids. 9. [1] The Enantioselective Generation of Tetracyclic ABCE Intermediates by a Tandem Condensation, [3,3]-Sigmatropic Rearrangement, and Cyclization Sequence" *J. Org. Chem.* **1997**, *62*, 7950–7960.

[61] J. Quetin-Leclercq, C. Coune, C. Delaude, R. Warin, R. Bassleer, L. Angenot, “Revision of the structure of strychnofluorine, an alkaloid of Strychnos gossweileri” *Phytochemistry* **1992**, *31*, 4347–4349.

[62] C. Mirand, G. Massiot, L. Le Men-Olivier, J. Levy, “The markovnikoff hydroboration of a trisubstituted olefin” *Tetrahedron Lett.* **1982**, *23*, 1257–1258.

[63] R. Robinson, F. A. L. Anet, “Conversion of the Wieland-Gumlich aldehyde into strychnine” *Chem Ind* **1953**, *11*, 245–245.

[64] B. Hong, D. Grzech, L. Caputi, P. Sonawane, C. E. R. López, M. O. Kamileen, N. J. Hernández Lozada, V. Grabe, S. E. O’Connor, “Biosynthesis of strychnine” *Nature* **2022**, *607*, 617–622.

Spectra

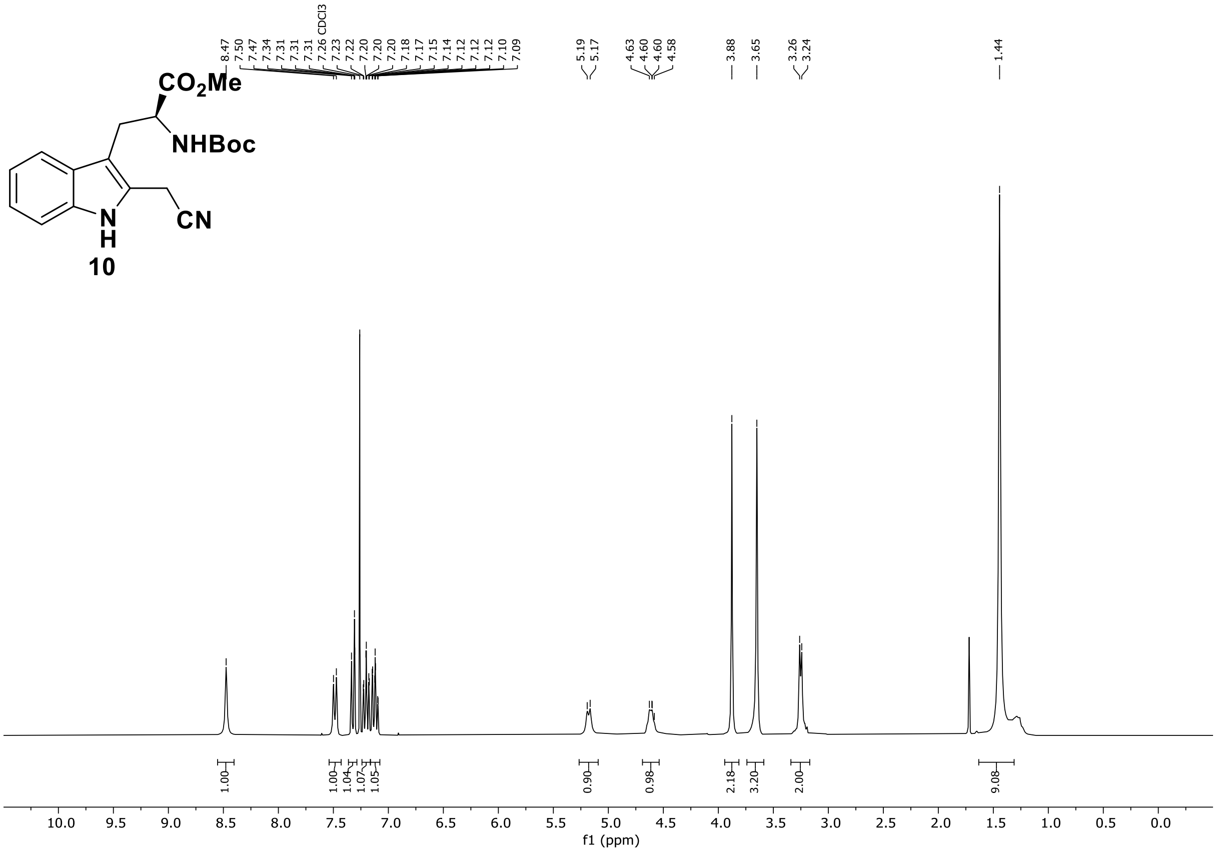

Figure S 2: ^{1}H NMR ($CDCl_3$, 300 MHz, 296 K) of compound **10**.

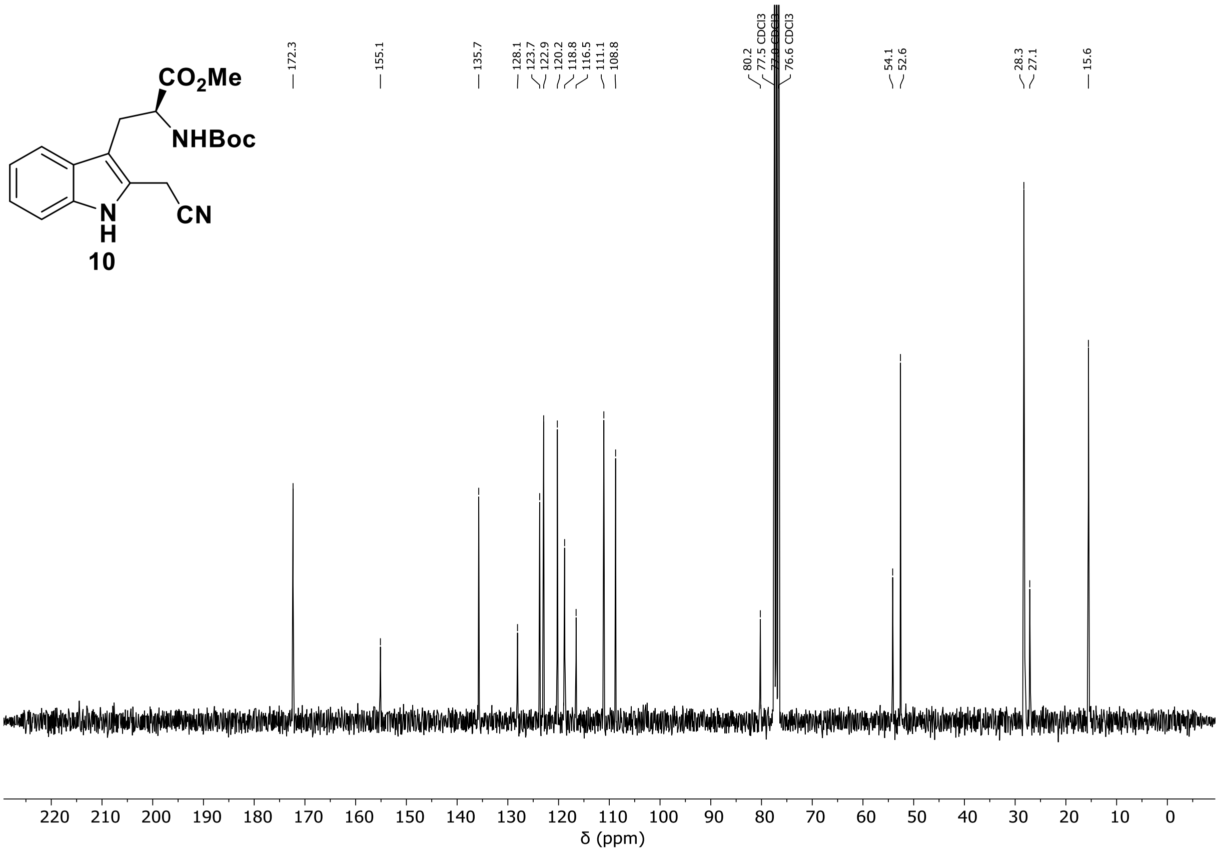

Figure S 3: $^{13}C\{^{1}H\}$ NMR ($CDCl_3$, 75 MHz, 296 K) of compound **10**.

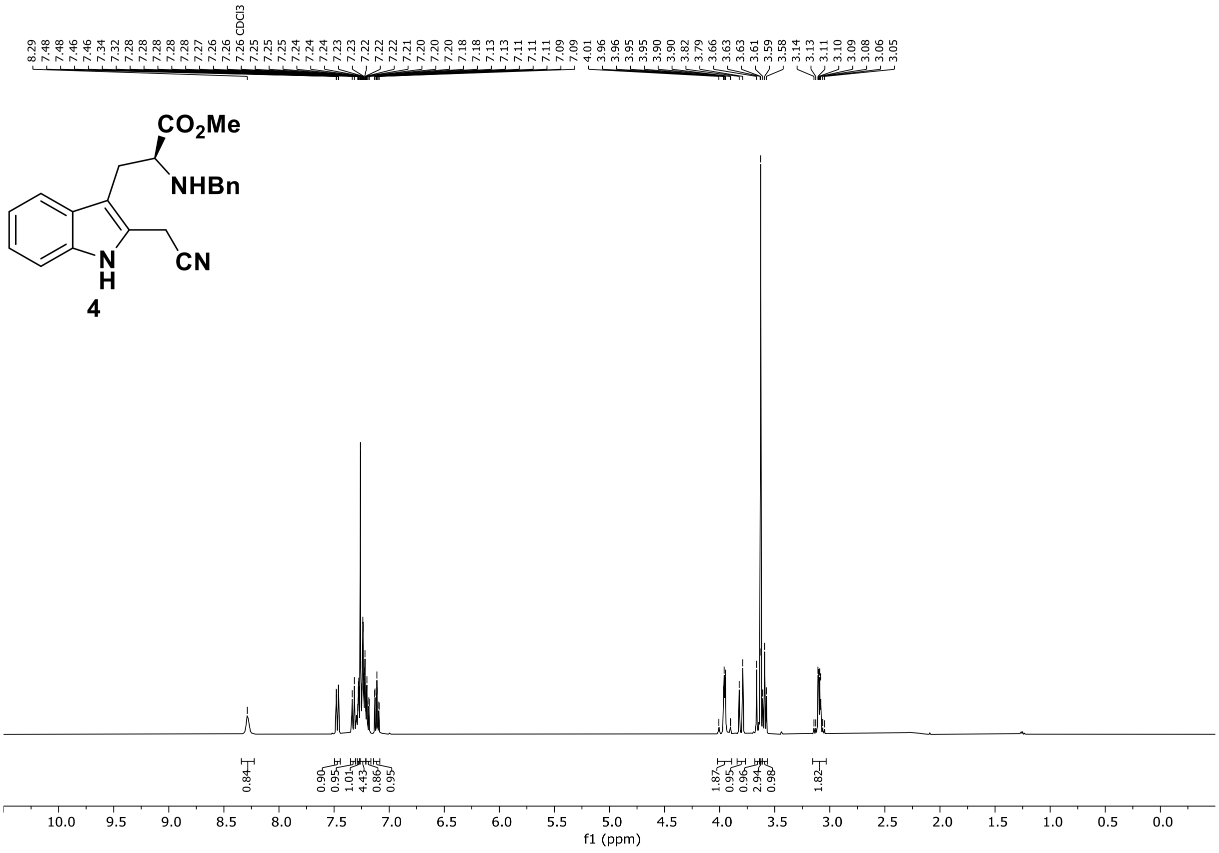

Figure S 4: ^{1}H NMR ($CDCl_3$, 400 MHz, 296 K) of compound **4**.

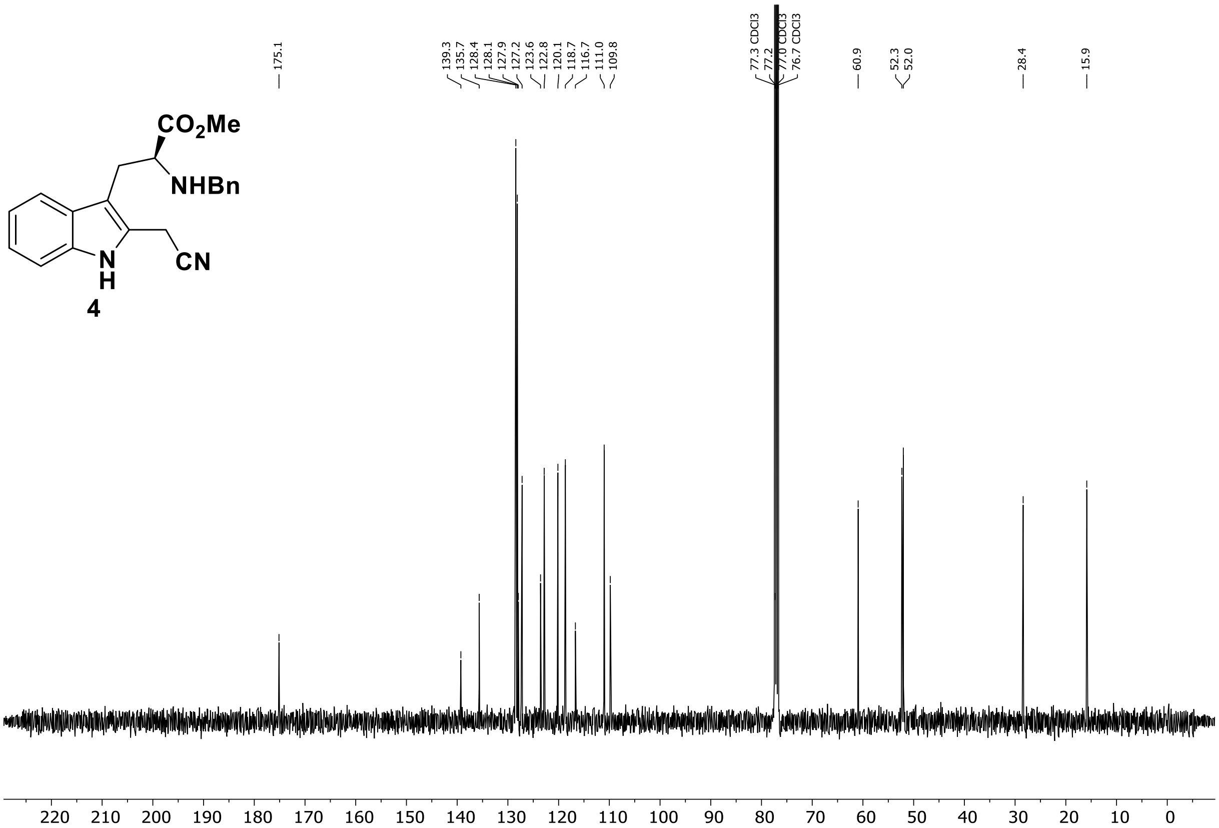

Figure S 5: $^{13}C\{^{1}H\}$ NMR ($CDCl_3$, 101 MHz, 296 K) of compound **4**.

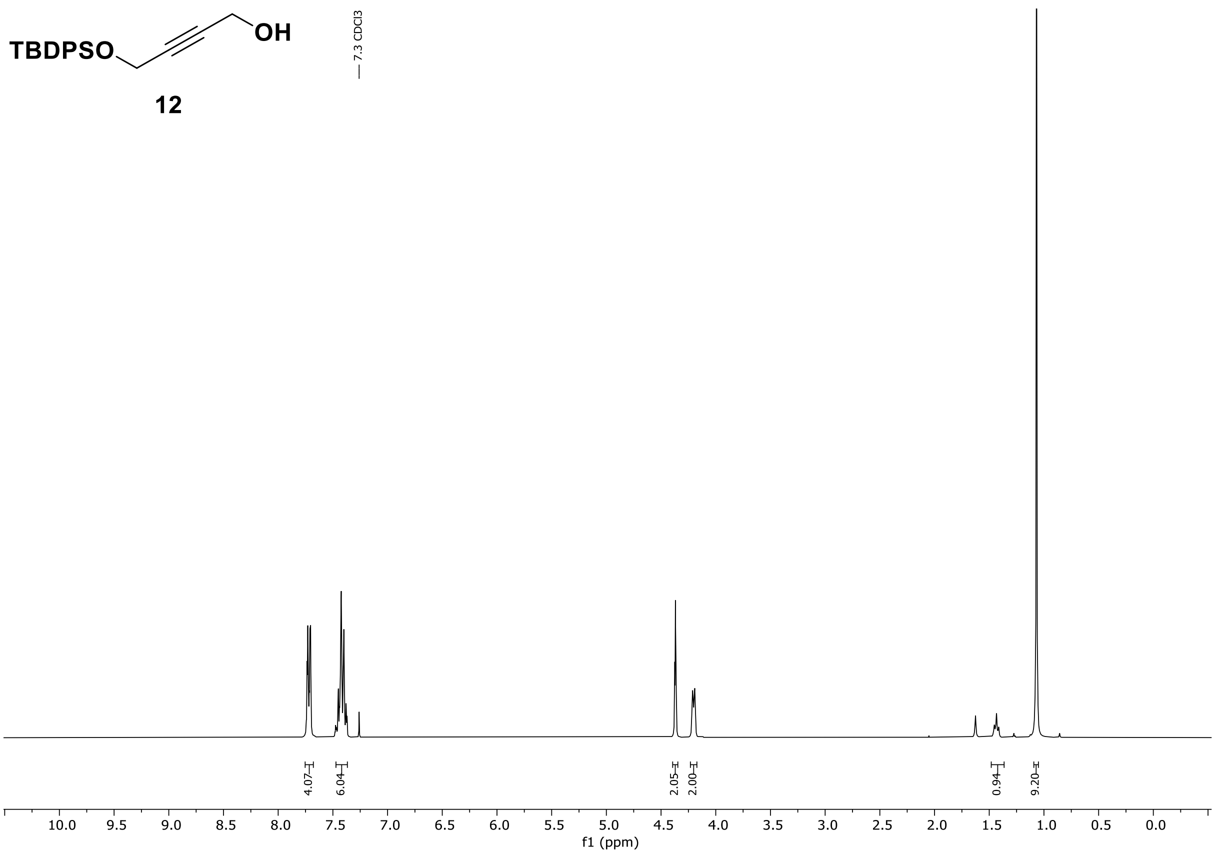

Figure S 6: ^{1}H NMR ($CDCl_3$, 300 MHz, 296 K) of compound **12**.

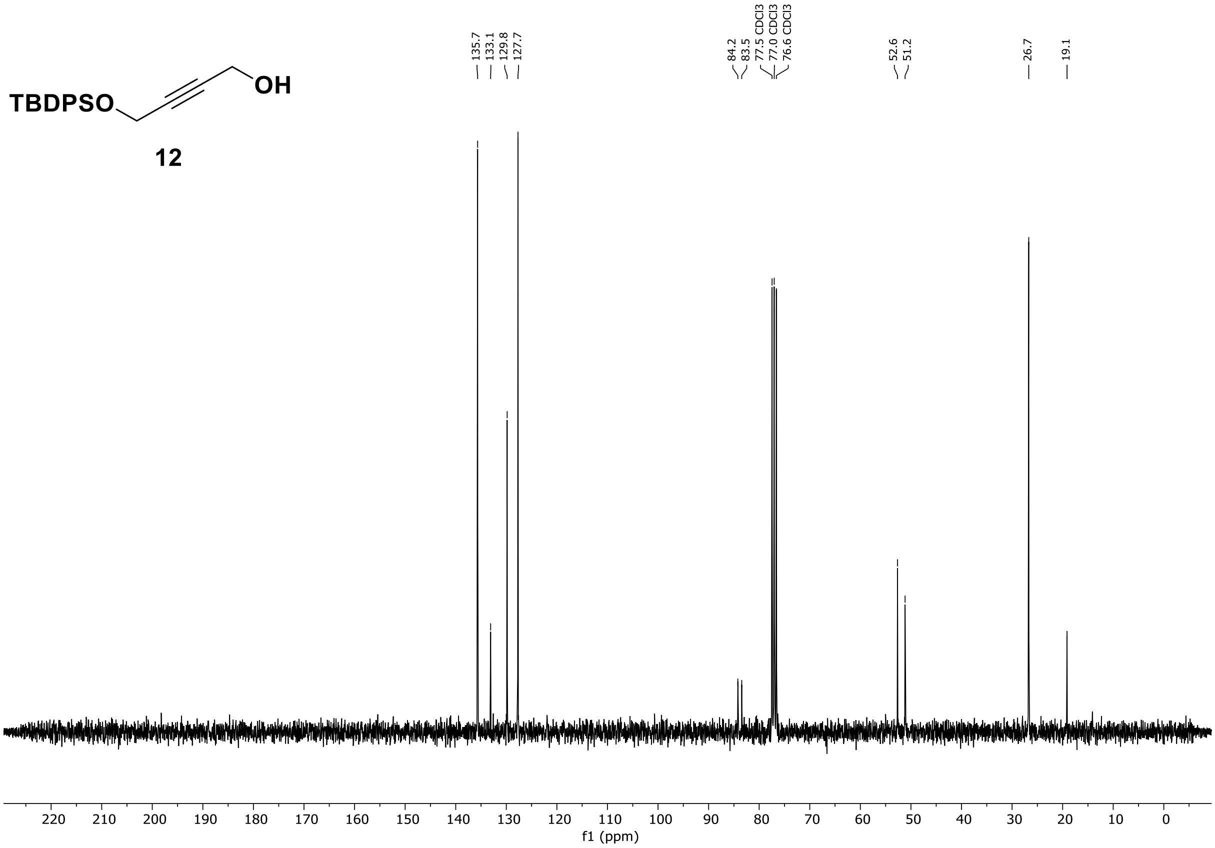

Figure S 7: $^{13}C\{^{1}H\}$ NMR ($CDCl_3$, 75 MHz, 296 K) of compound **12**.

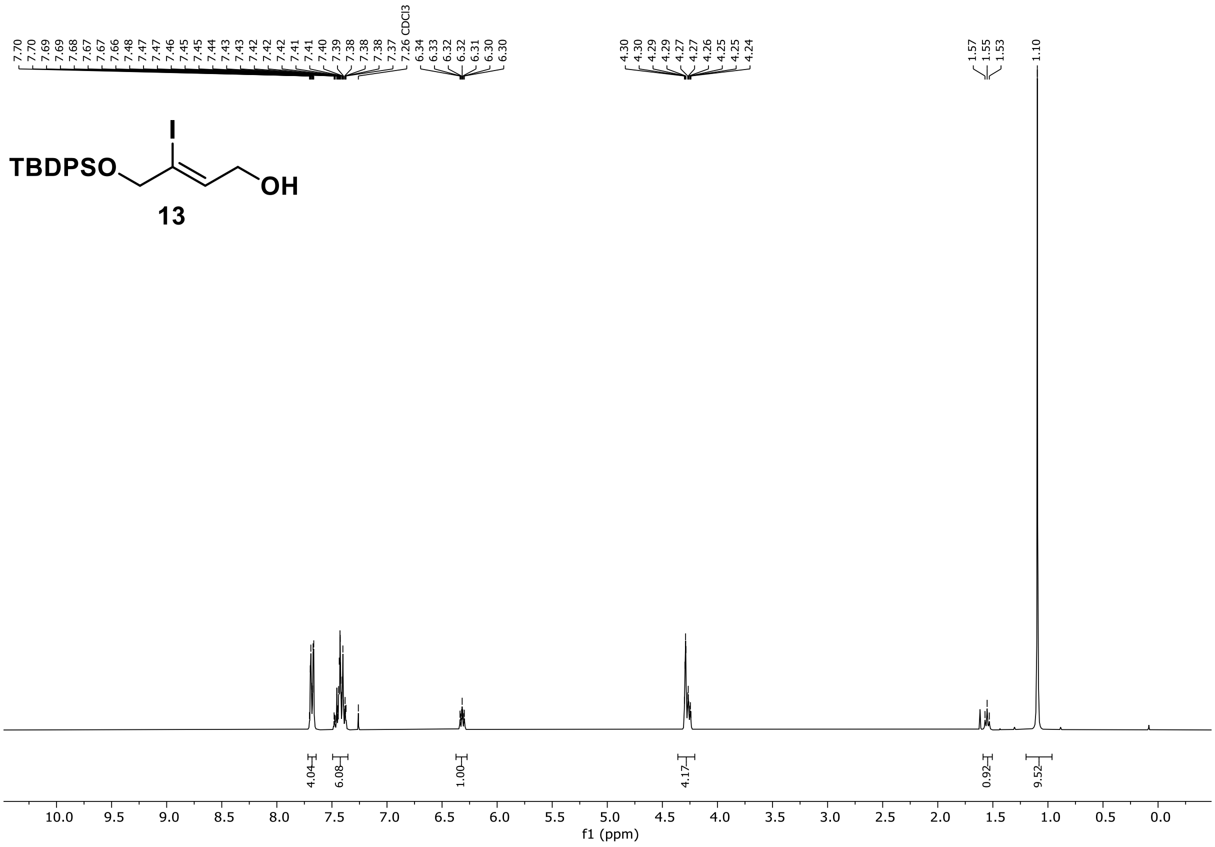

Figure S 8: ^{1}H NMR ($CDCl_3$, 300 MHz, 296 K) of compound **13**.

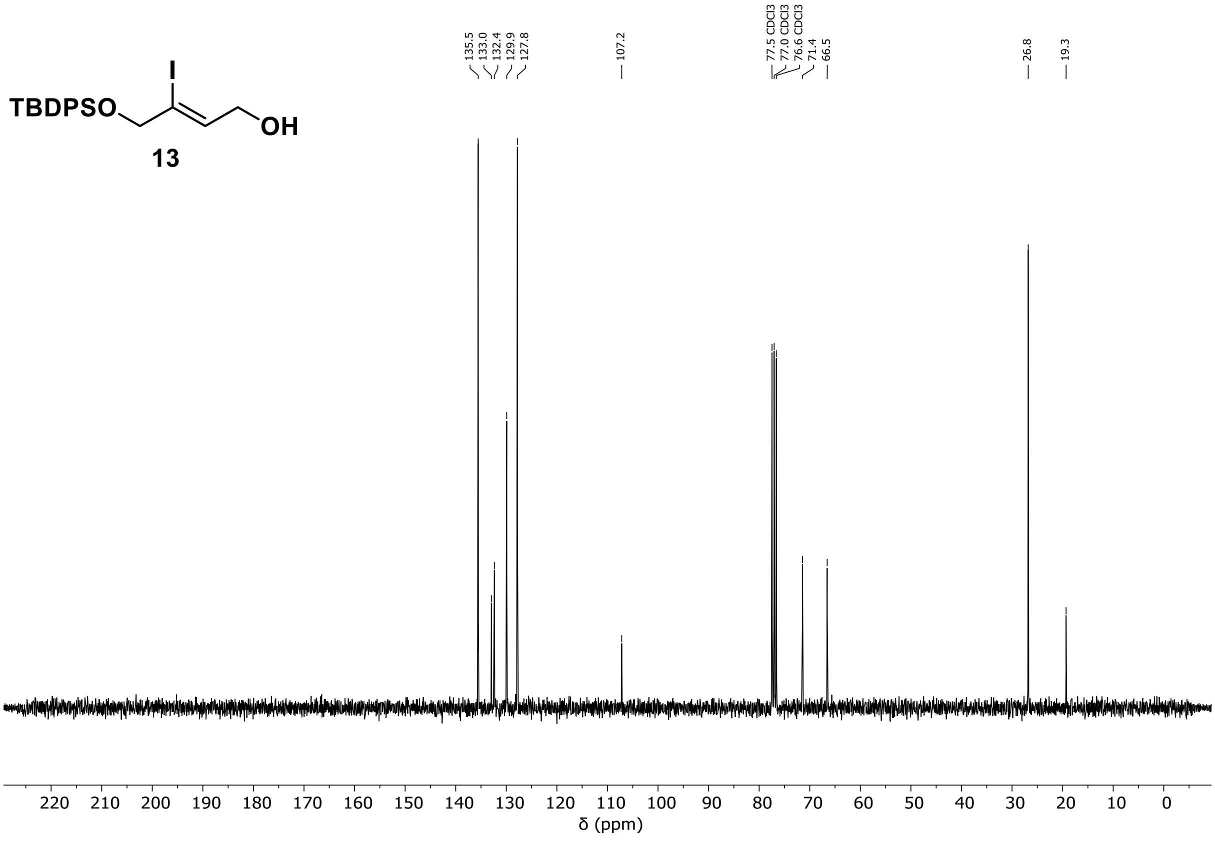

Figure S 9: $^{13}C\{^{1}H\}$ NMR ($CDCl_3$, 75 MHz, 296 K) of compound **13**.

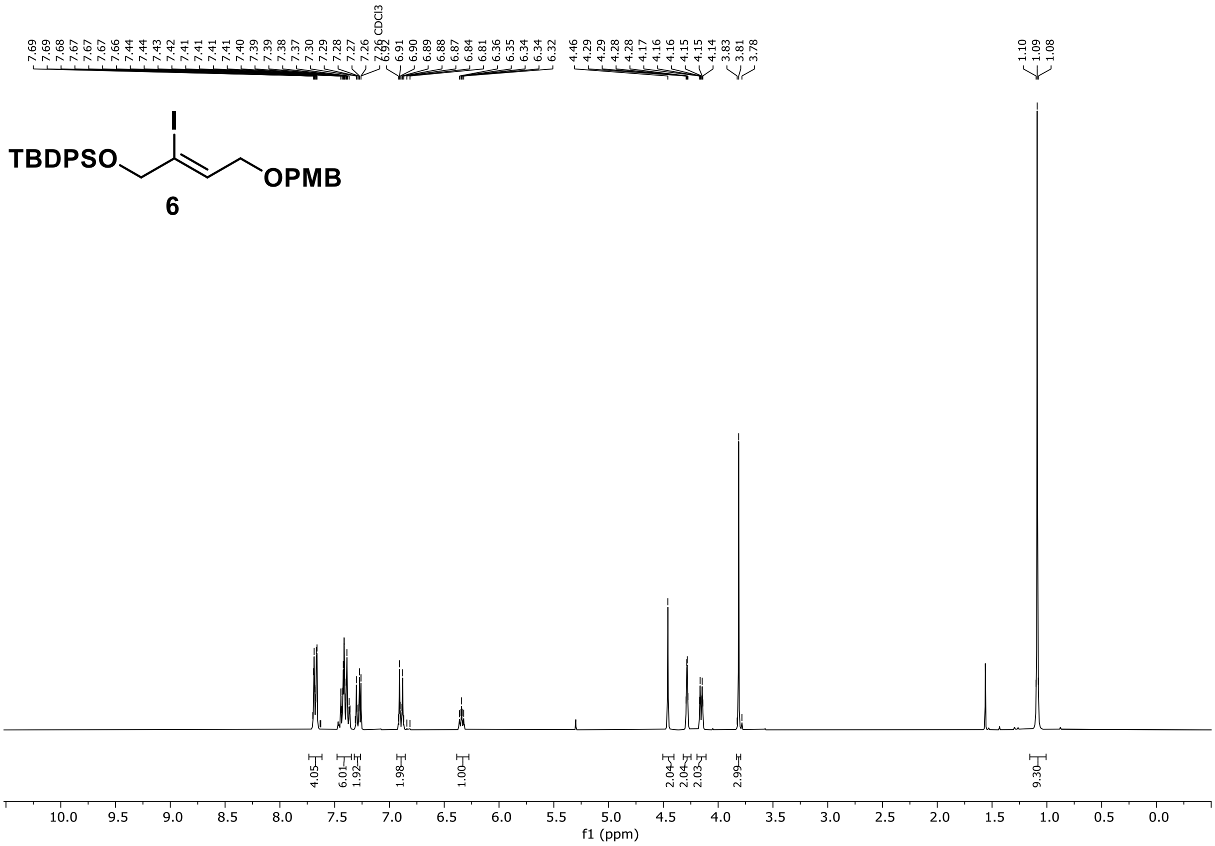

Figure S 10: ^{1}H NMR ($CDCl_3$, 300 MHz, 296 K) of compound **6**.

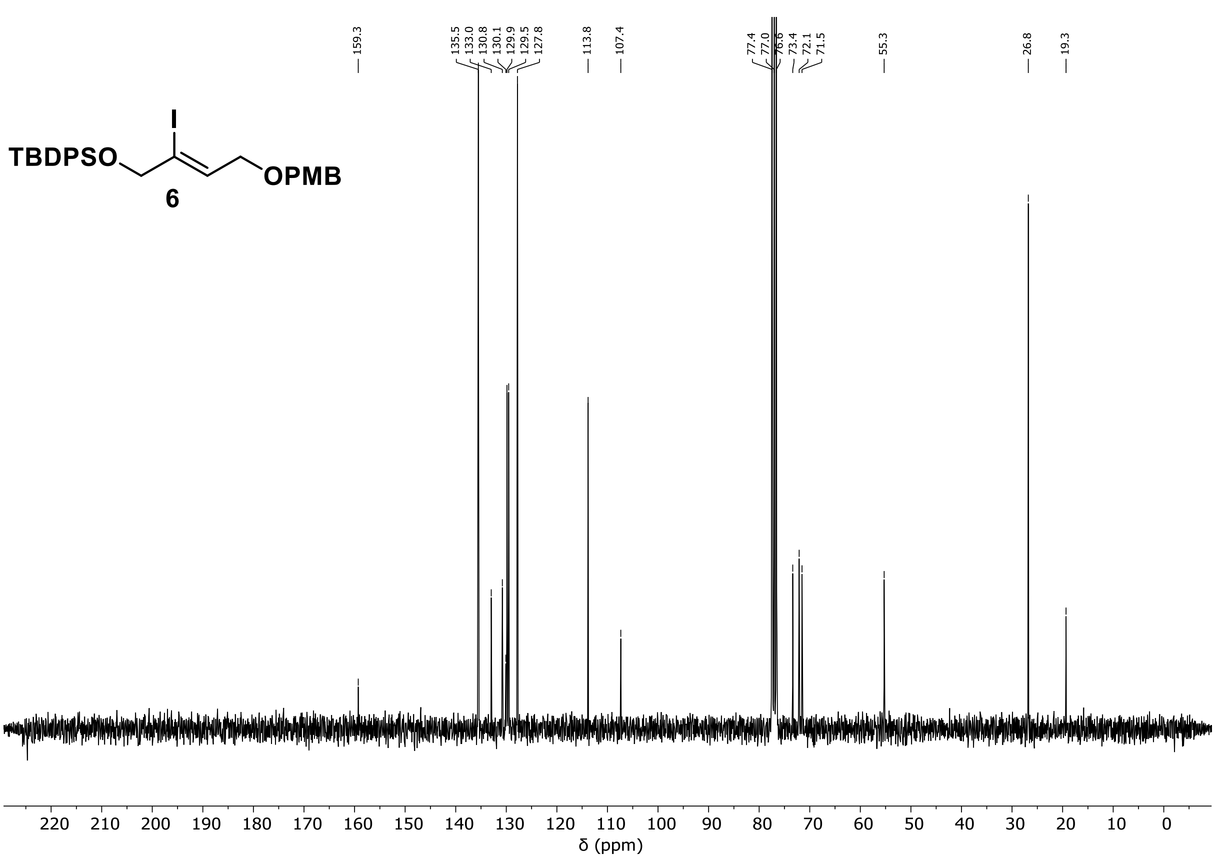

Figure S 11: $^{13}C\{^{1}H\}$ NMR ($CDCl_3$, 75 MHz, 296 K) of compound **6**.

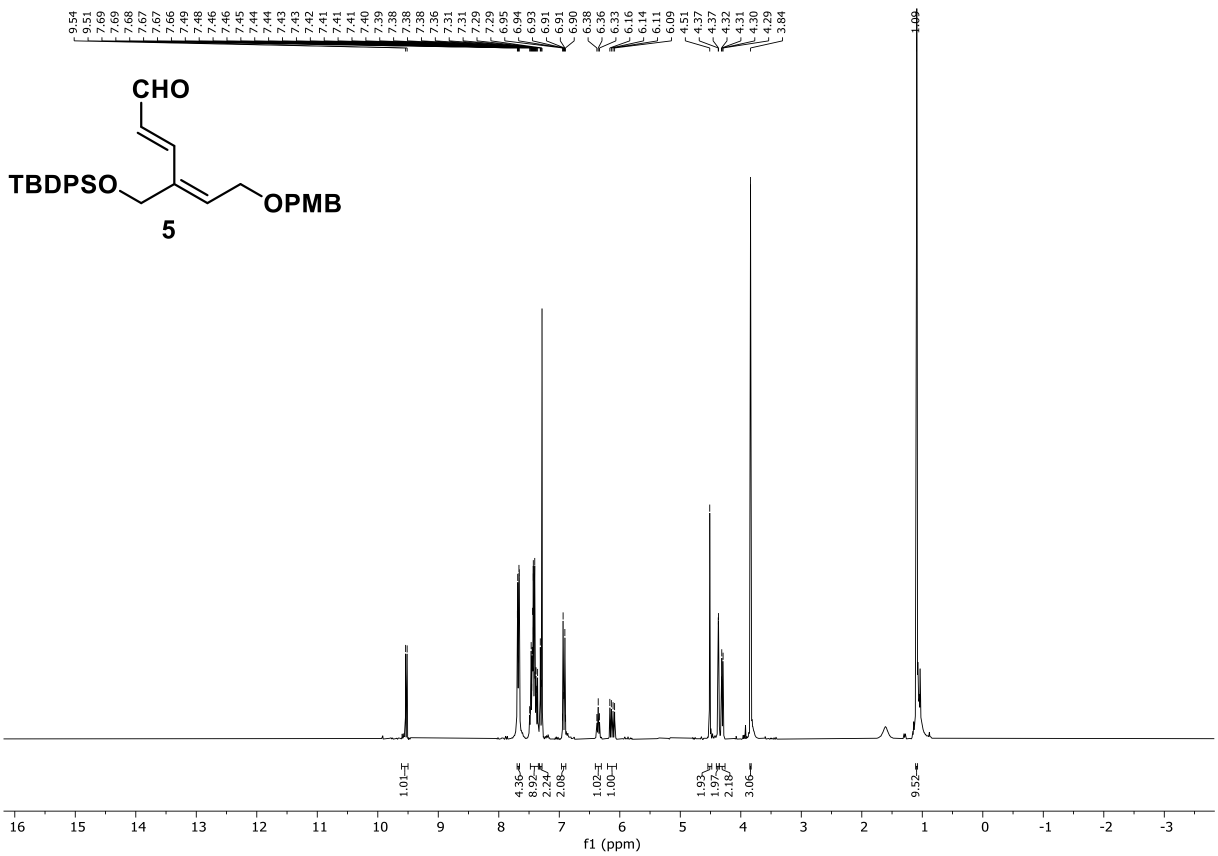

Figure S 12: ^{1}H NMR ($CDCl_3$, 300 MHz, 296 K) of compound **5**.

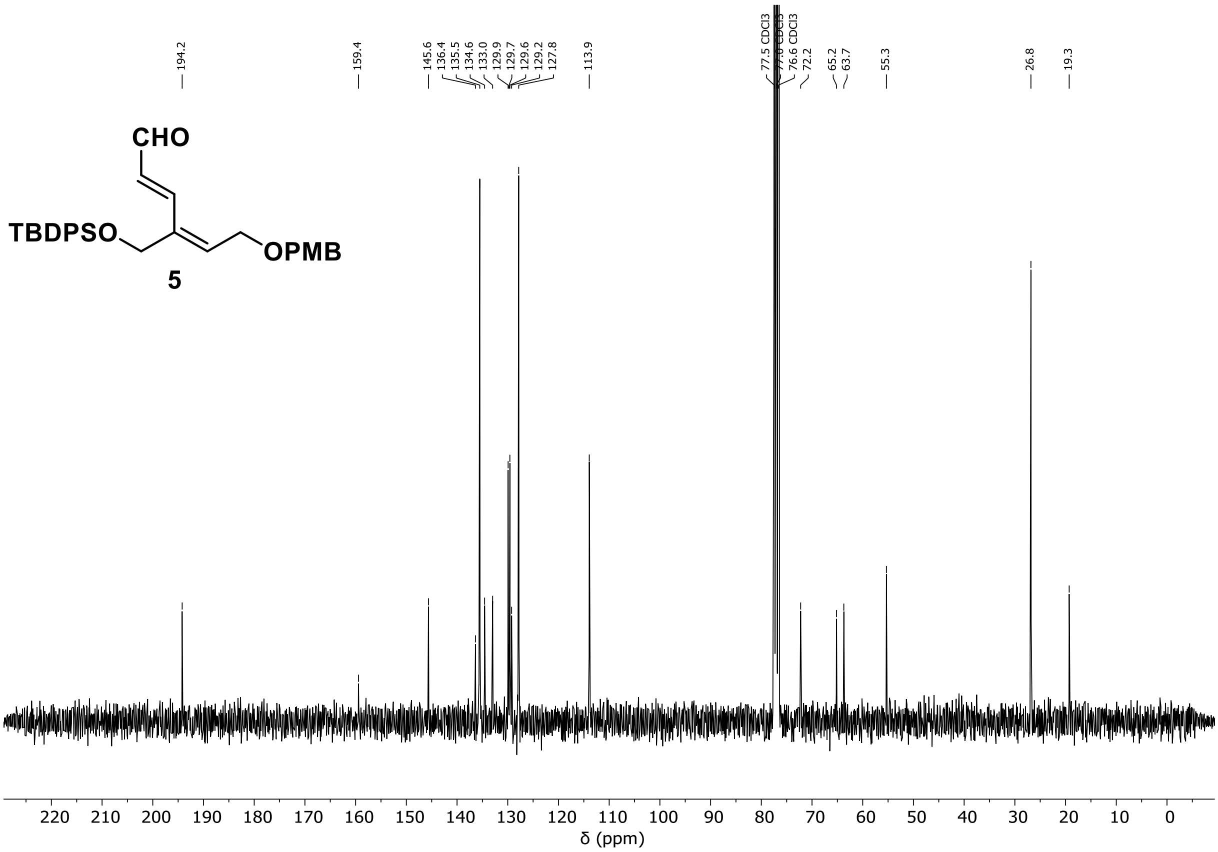

Figure S 13: $^{13}C\{^{1}H\}$ NMR ($CDCl_3$, 75 MHz, 296 K) of compound **5**.

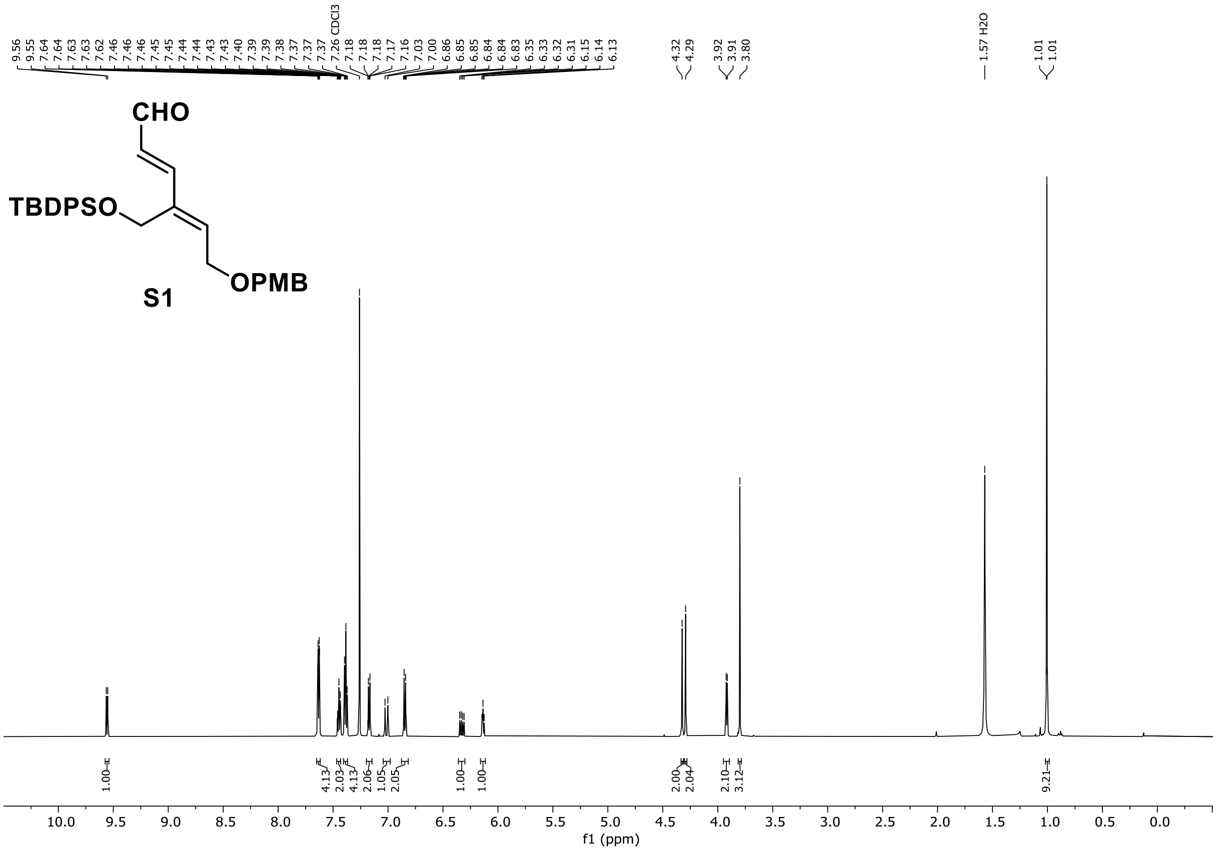

Figure S 14: ^{1}H NMR ($CDCL_3$, 600 MHz, 296 K) of compound **S1**.

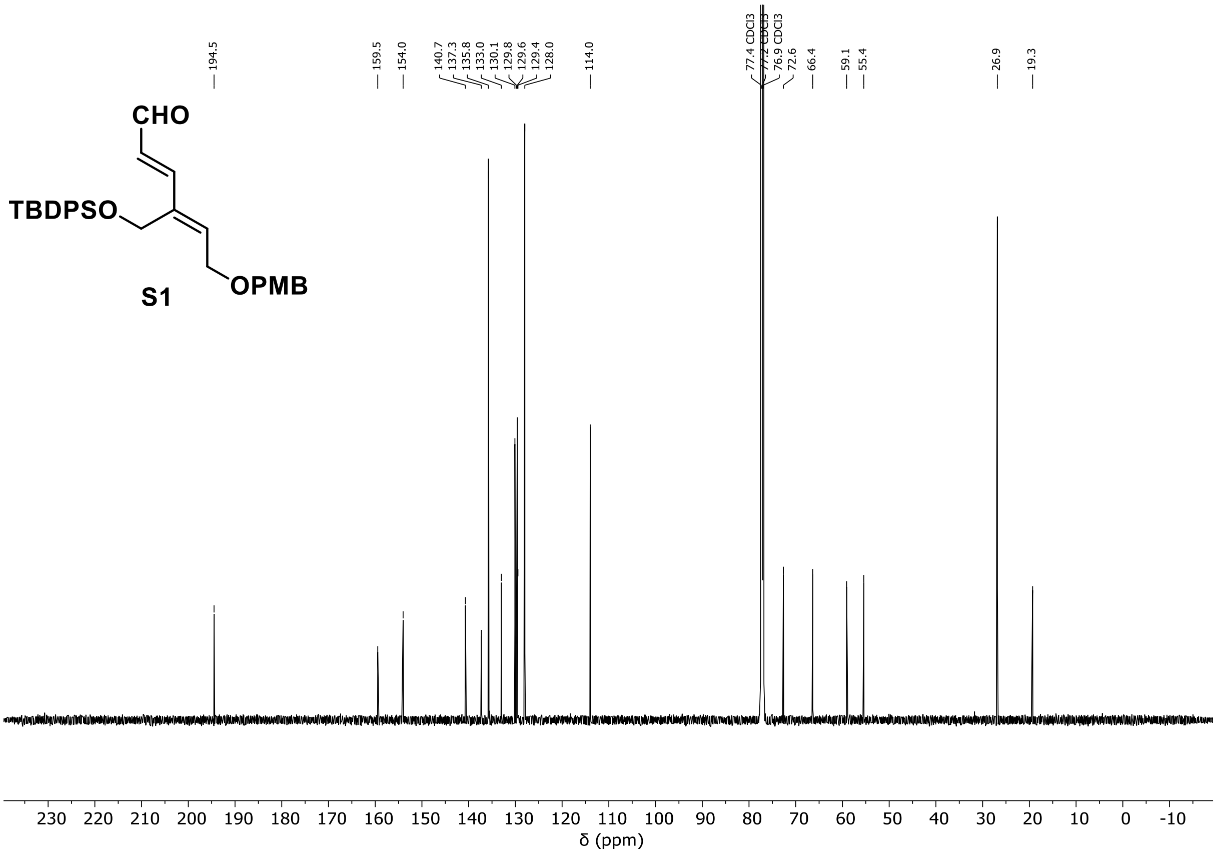

Figure S 15: $^{13}C\{^{1}H\}$ NMR ($CDCl_3$, 151 MHz, 296 K) of compound **S1**.

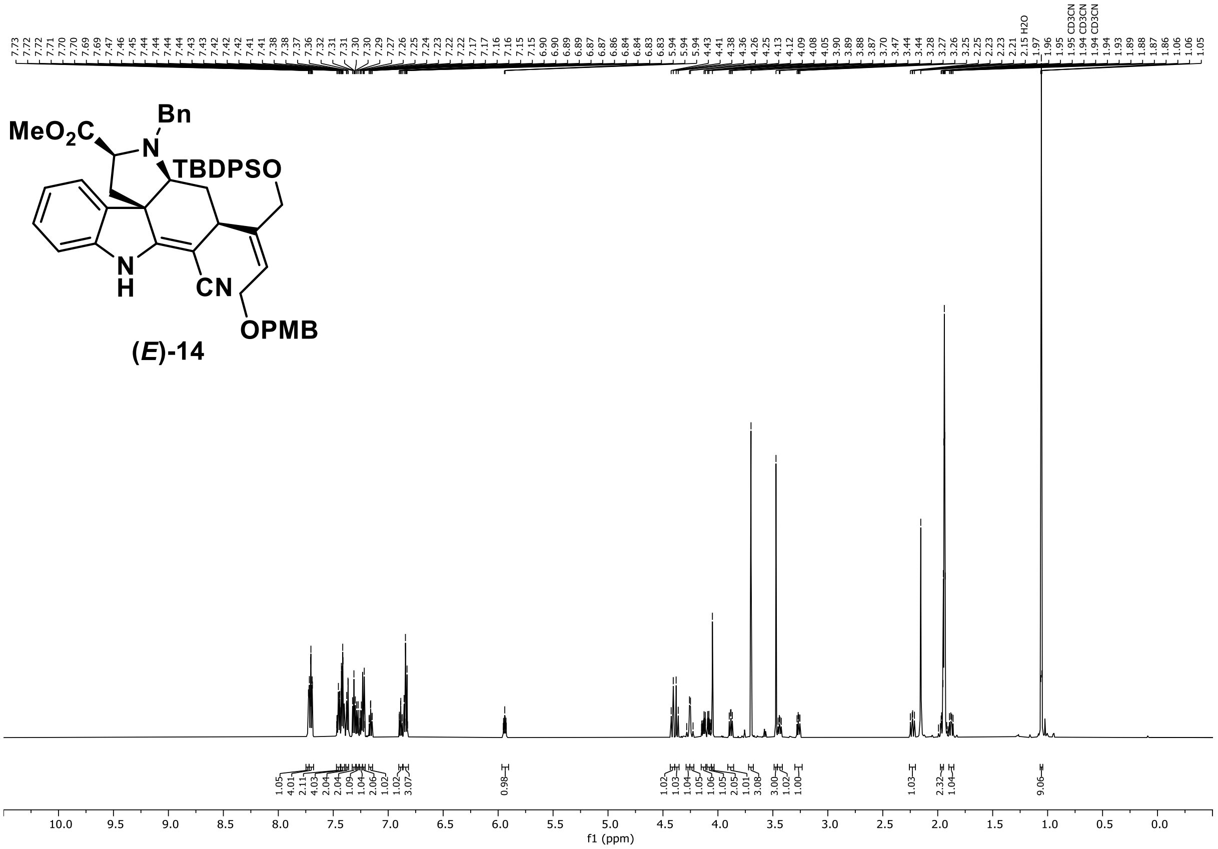

Figure S 16: ^{1}H NMR (CD_3CN, 600 MHz, 296 K) of compound **(*E*)-14**.

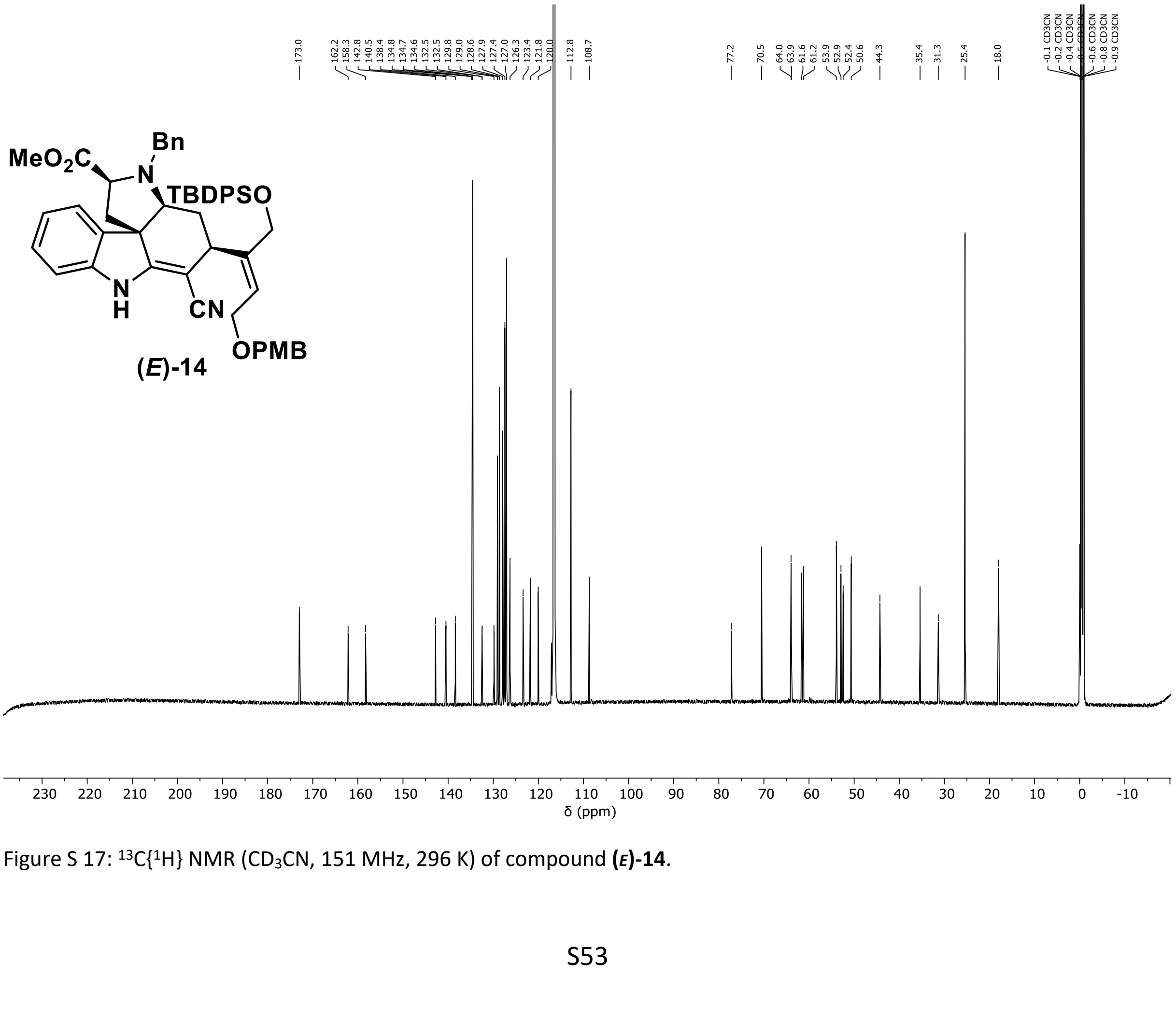

Figure S 17: $^{13}C\{^{1}H\}$ NMR (CD_3CN, 151 MHz, 296 K) of compound **(*E*)-14**.

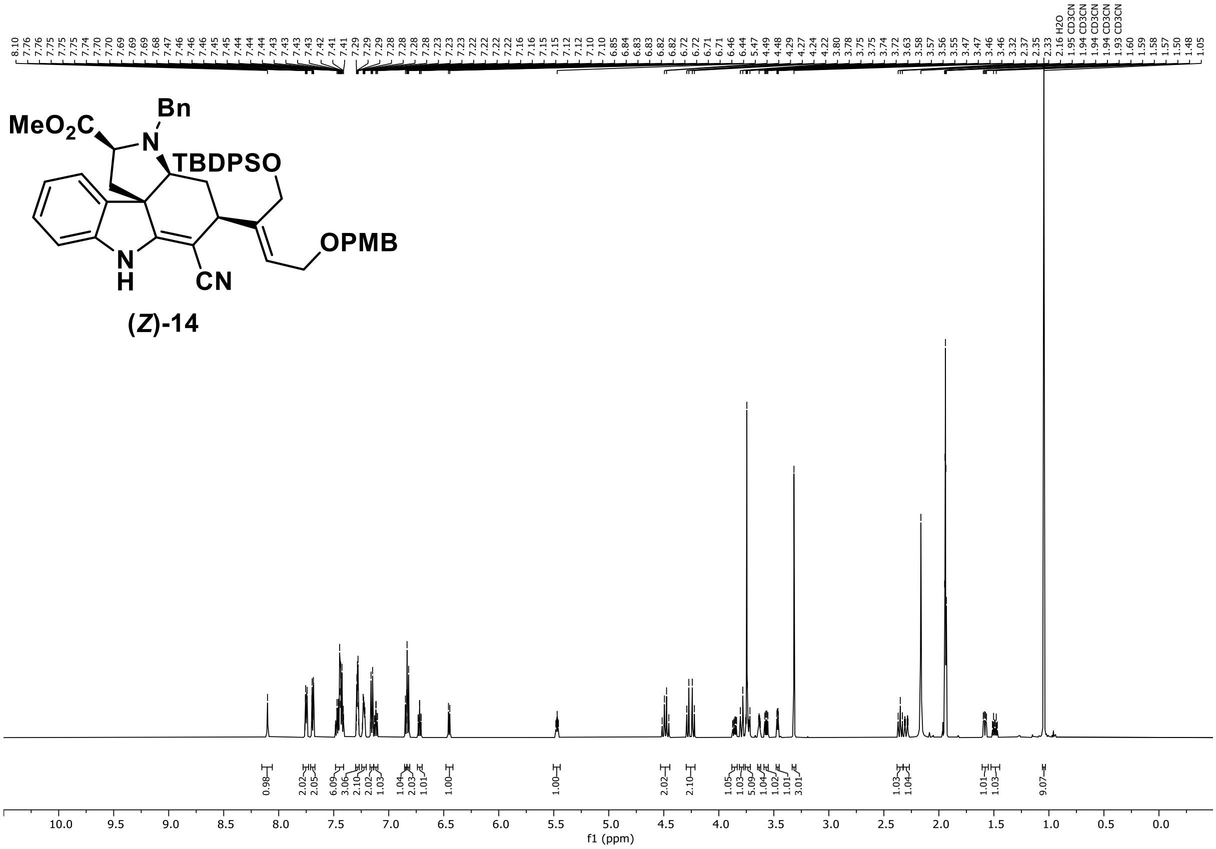

Figure S 18: ${}^{1}H$ NMR (CD_3CN, 600 MHz, 296 K) of compound **(*z*)-14**.

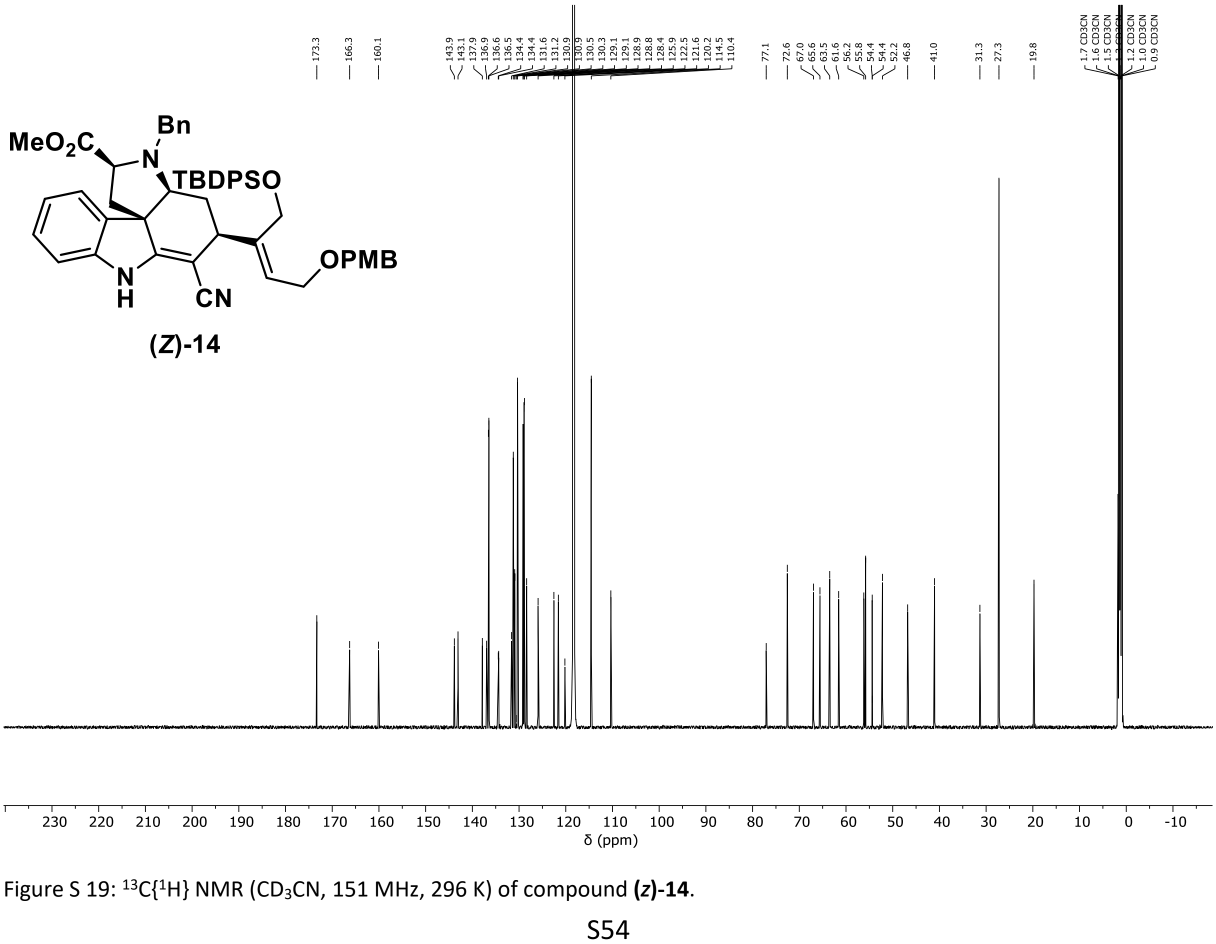

Figure S 19: ${}^{13}C\{{}^{1}H\}$ NMR (CD_3CN, 151 MHz, 296 K) of compound **(*z*)-14**.

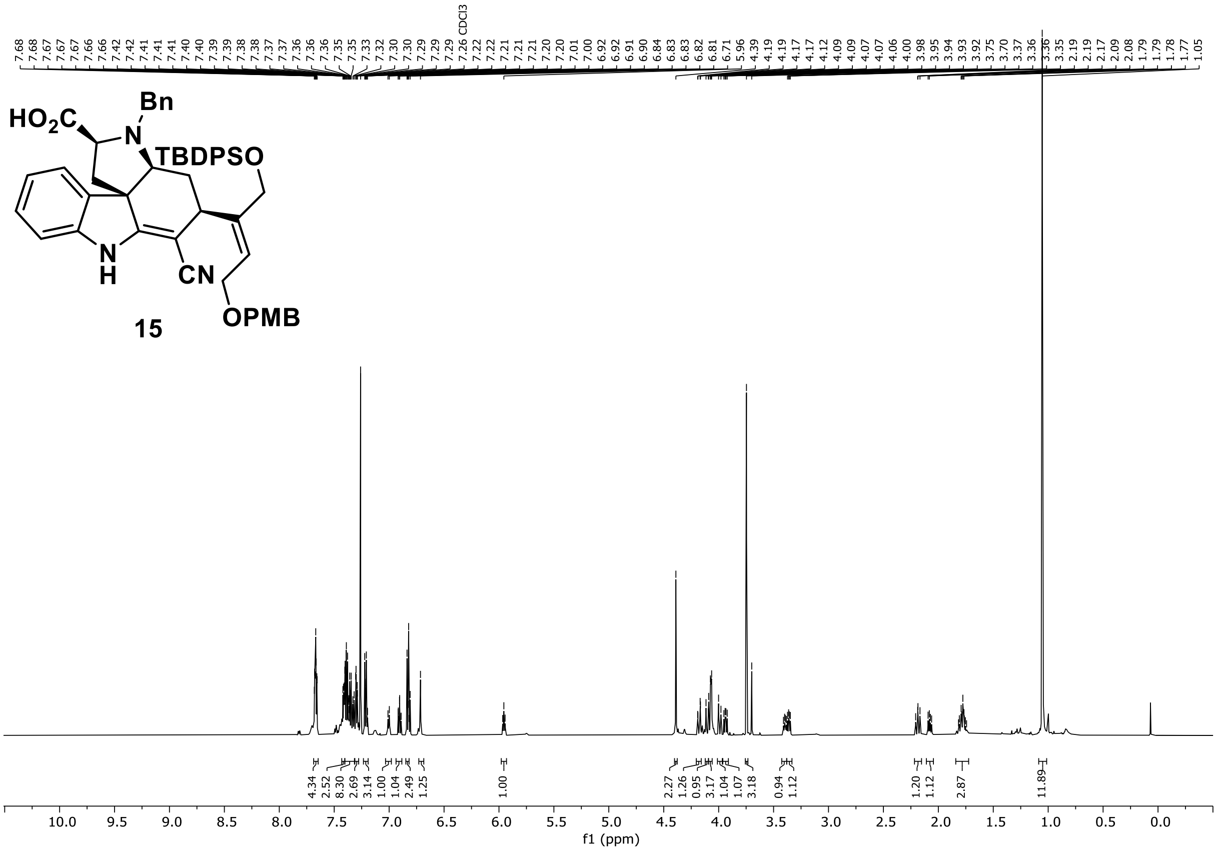

Figure S 20: ^{1}H NMR ($CDCl_3$, 600 MHz, 296 K) of compound **15**.

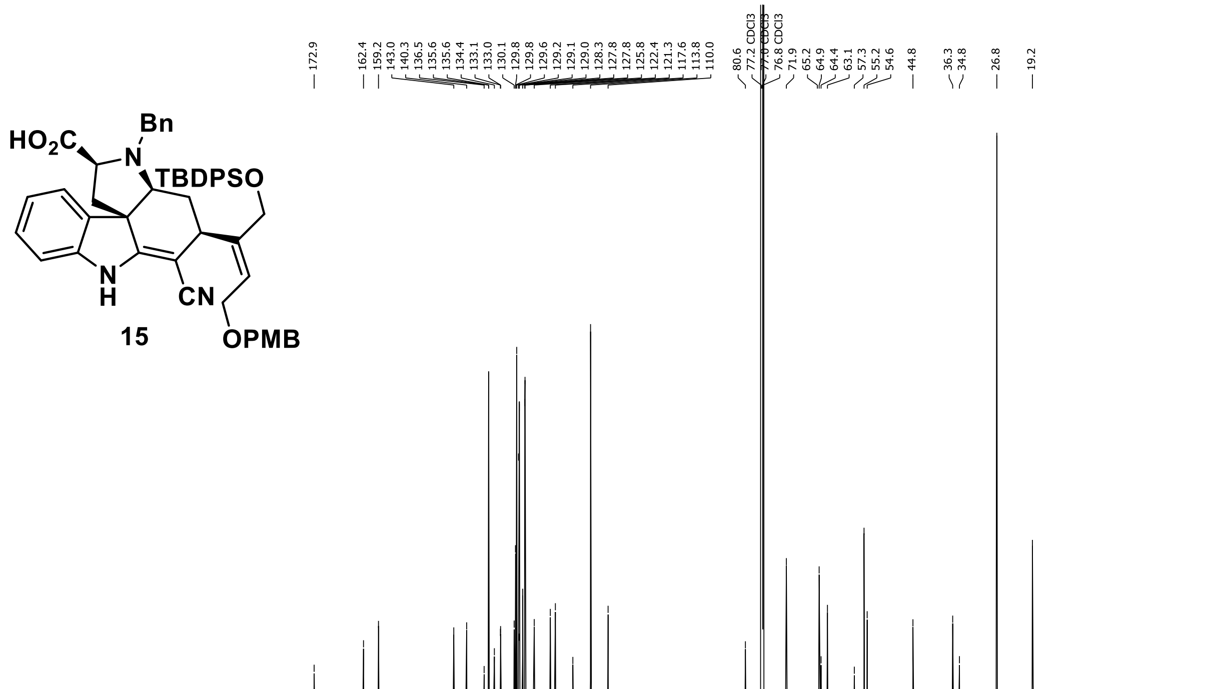

Figure S 21: $^{13}C\{^{1}H\}$ NMR ($CHCl_3$, 151 MHz, 296 K) of compound **15**.

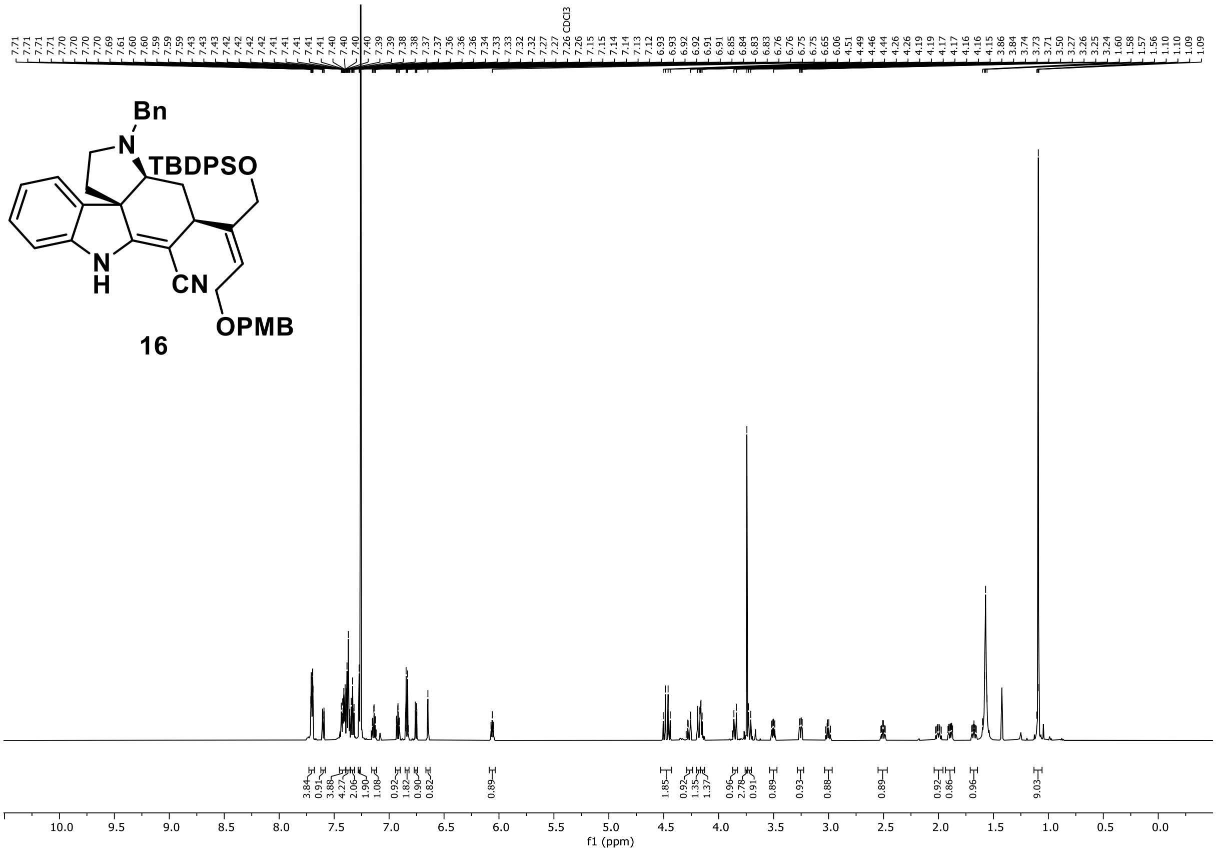

Figure S 22: ^{1}H NMR ($CDCl_3$, 600 MHz, 296 K) of compound **16**.

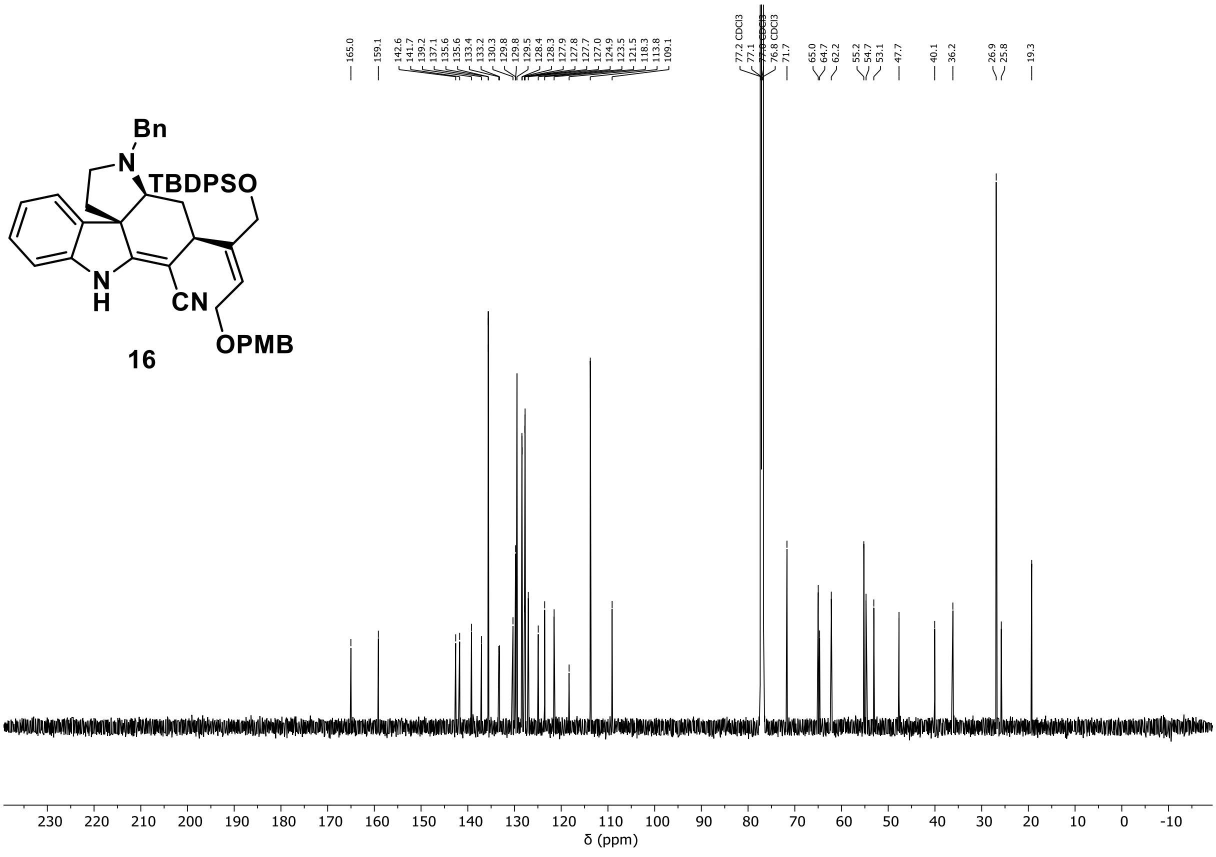

Figure S 23: $^{13}C\{^{1}H\}$ NMR ($CHCl_3$, 151 MHz, 296 K) of compound **16**.

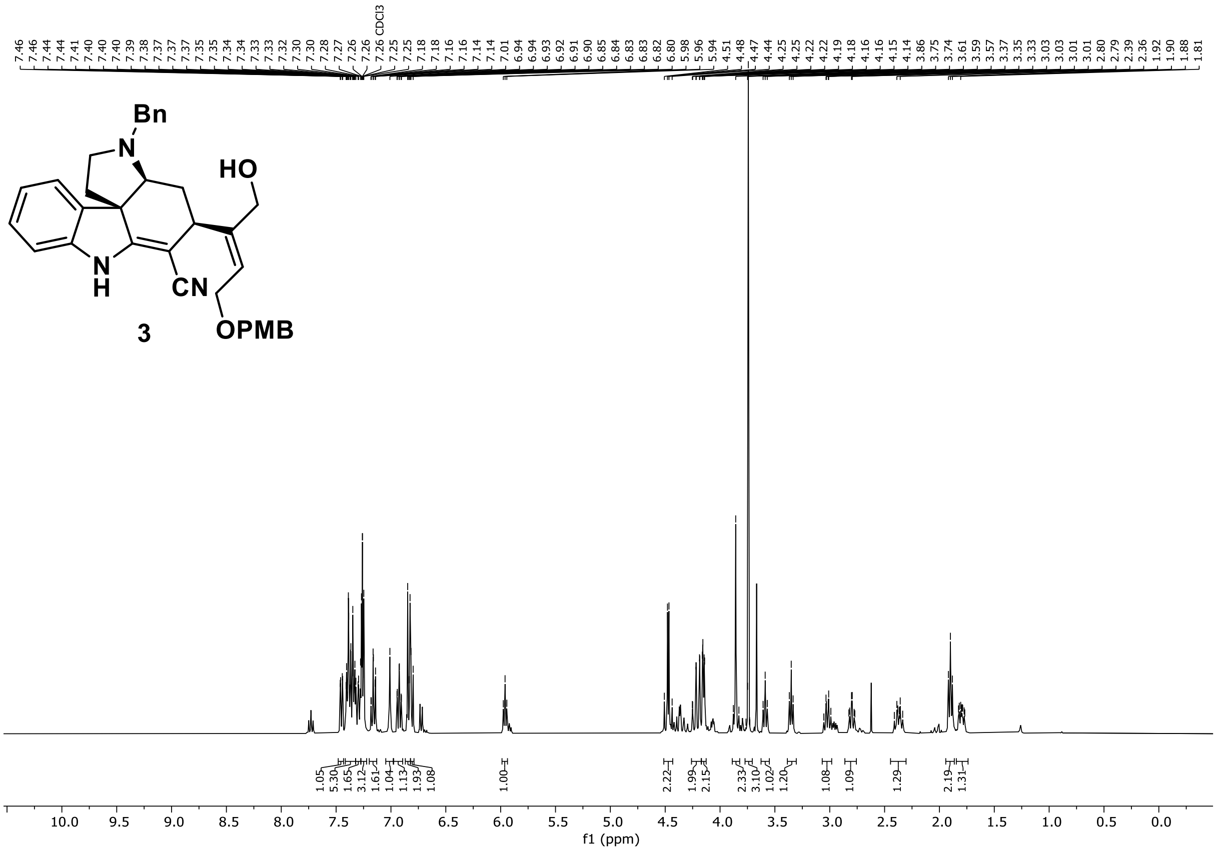

Figure S 24: ^{1}H NMR ($CDCl_3$, 400 MHz, 296 K) of compound **3**. **3** was obtained as a non-separatable mixture with its hydrolysis product the corresponding amide.

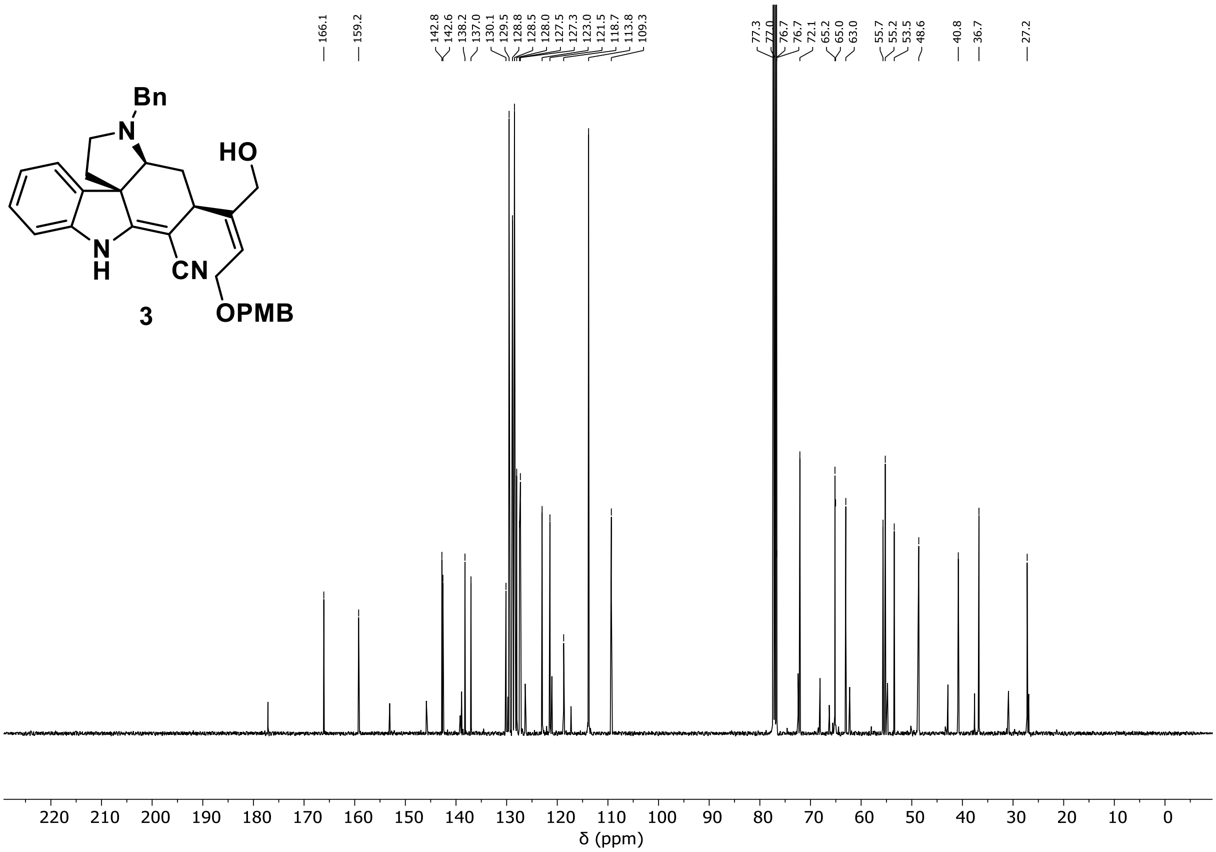

Figure S 25: $^{13}C\{^1H\}$ NMR ($CHCl_3$, 101 MHz, 296 K) of compound **3**. **3** was obtained as a non-separatable mixture with its hydrolysis product, the corresponding amide.

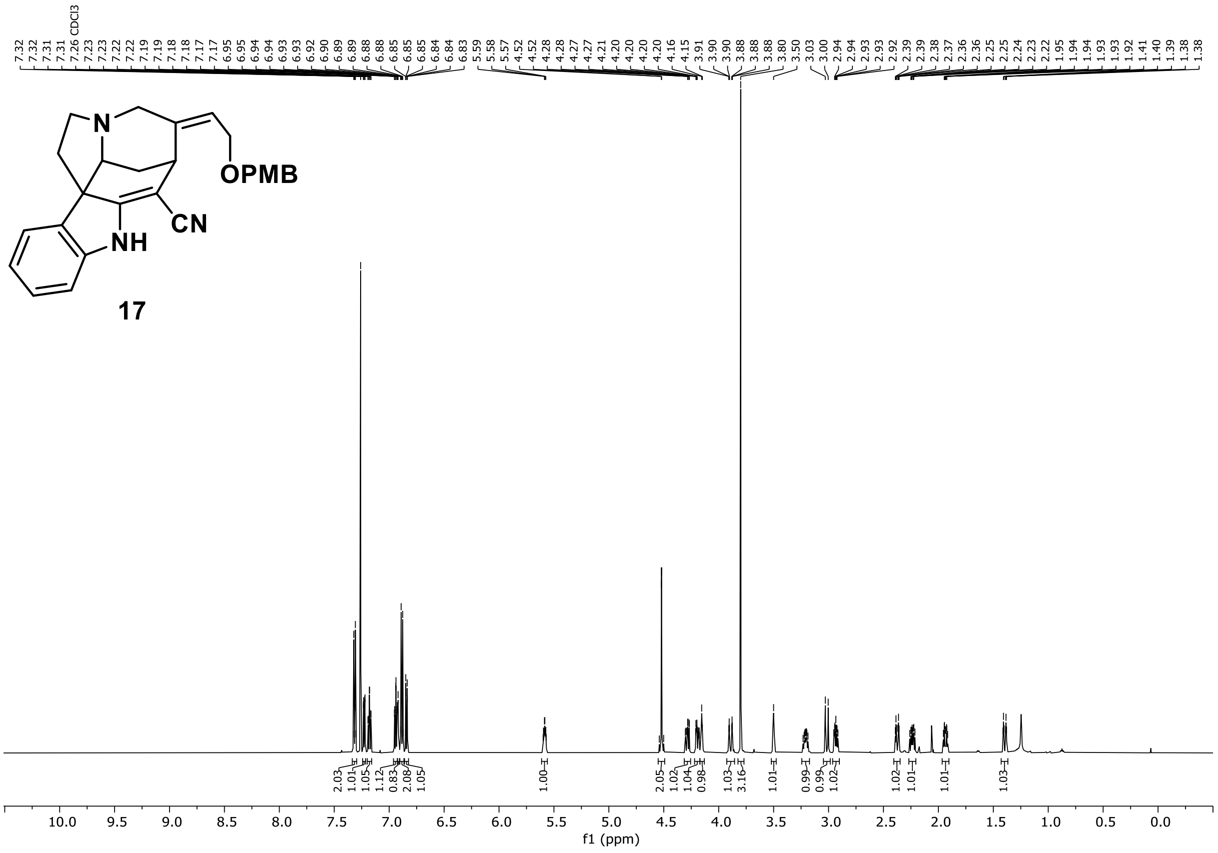

Figure S 26: ^{1}H NMR ($CDCl_3$, 600 MHz, 296 K) of compound **17**.

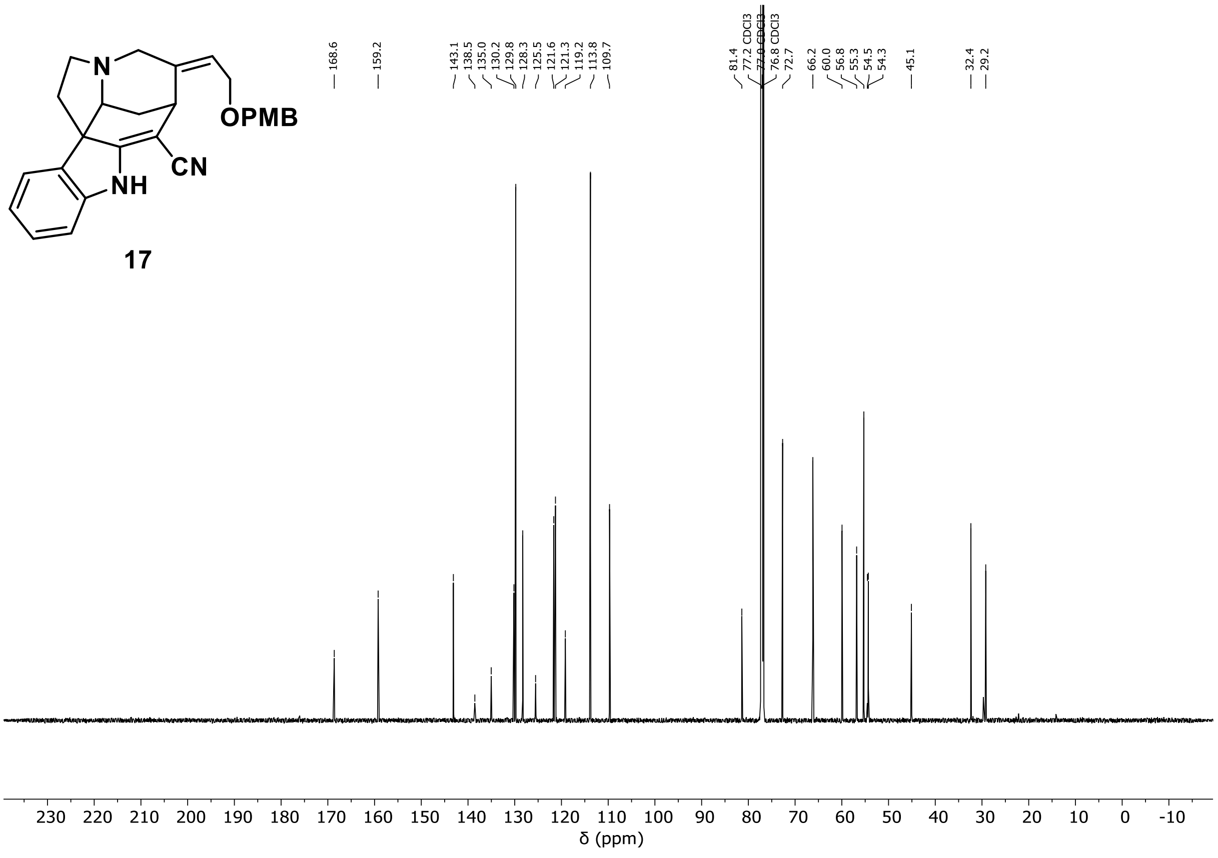

Figure S 27: $^{13}C\{^{1}H\}$ NMR ($CHCl_3$, 151 MHz, 296 K) of compound **17**.

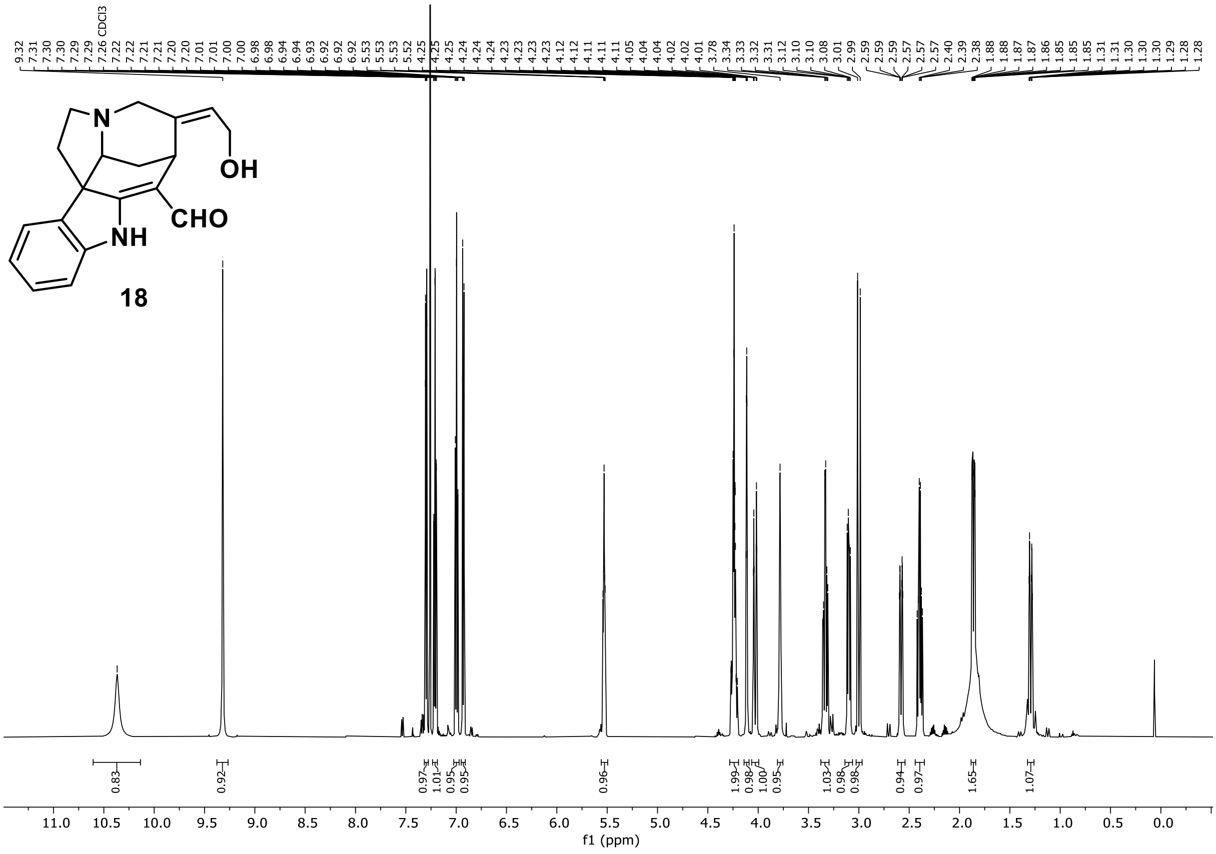

Figure S 28: ^{1}H NMR ($CDCl_3$, 600 MHz, 296 K) of compound **18**.

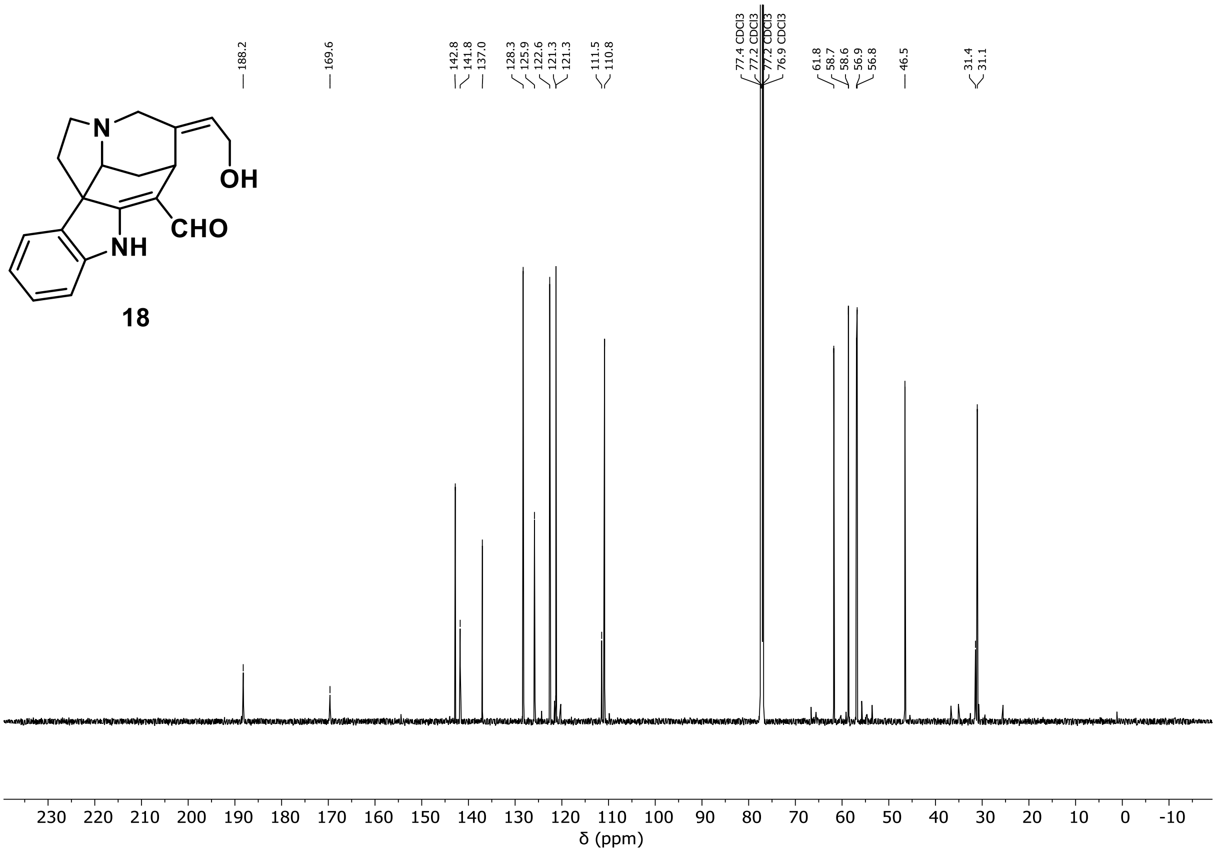

Figure S 29: $^{13}C\{^{1}H\}$ NMR ($CHCl_3$, 151 MHz, 296 K) of compound **18**.

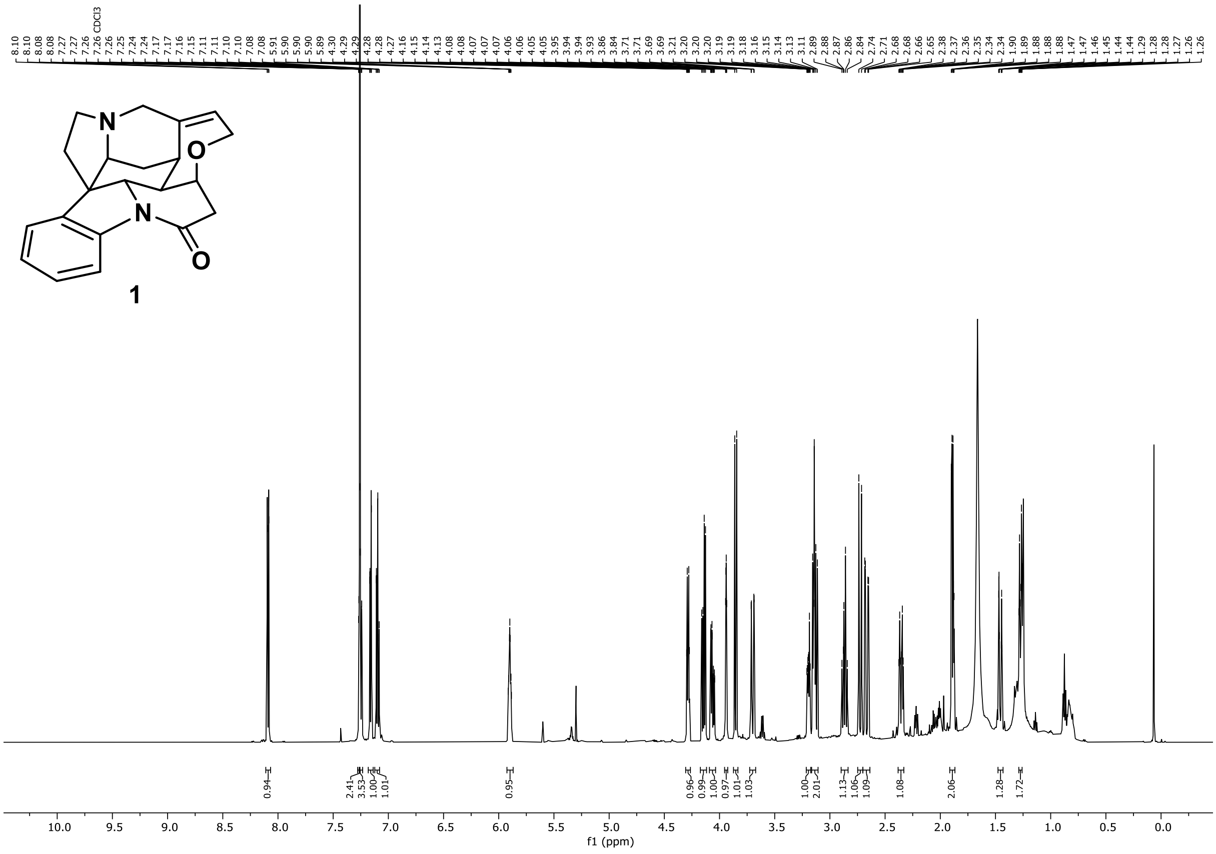

Figure S 30: ^{1}H NMR ($CDCl_3$, 600 MHz, 296 K) of compound **1**.

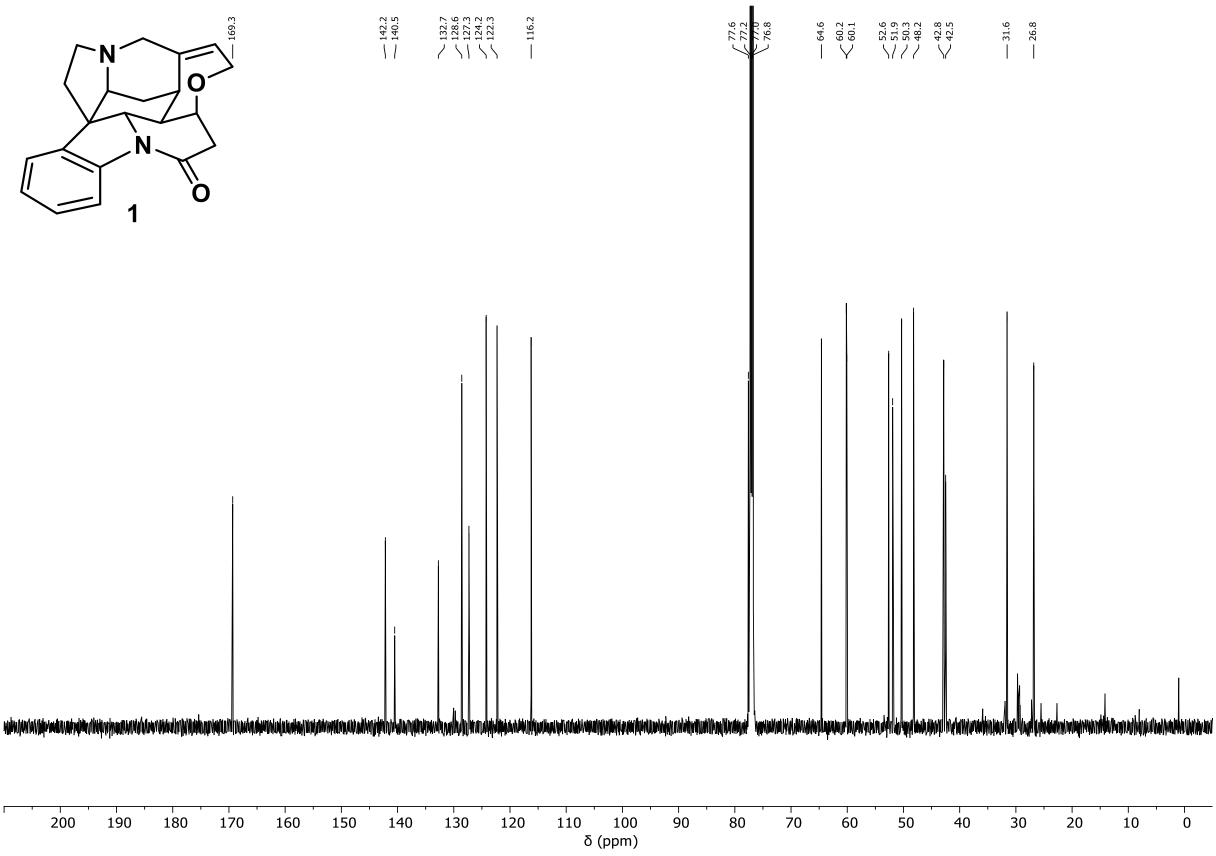

Figure S 31: $^{13}C\{^1H\}$ NMR ($CDCl_3$, 151 MHz, 296 K) of compound **1**.

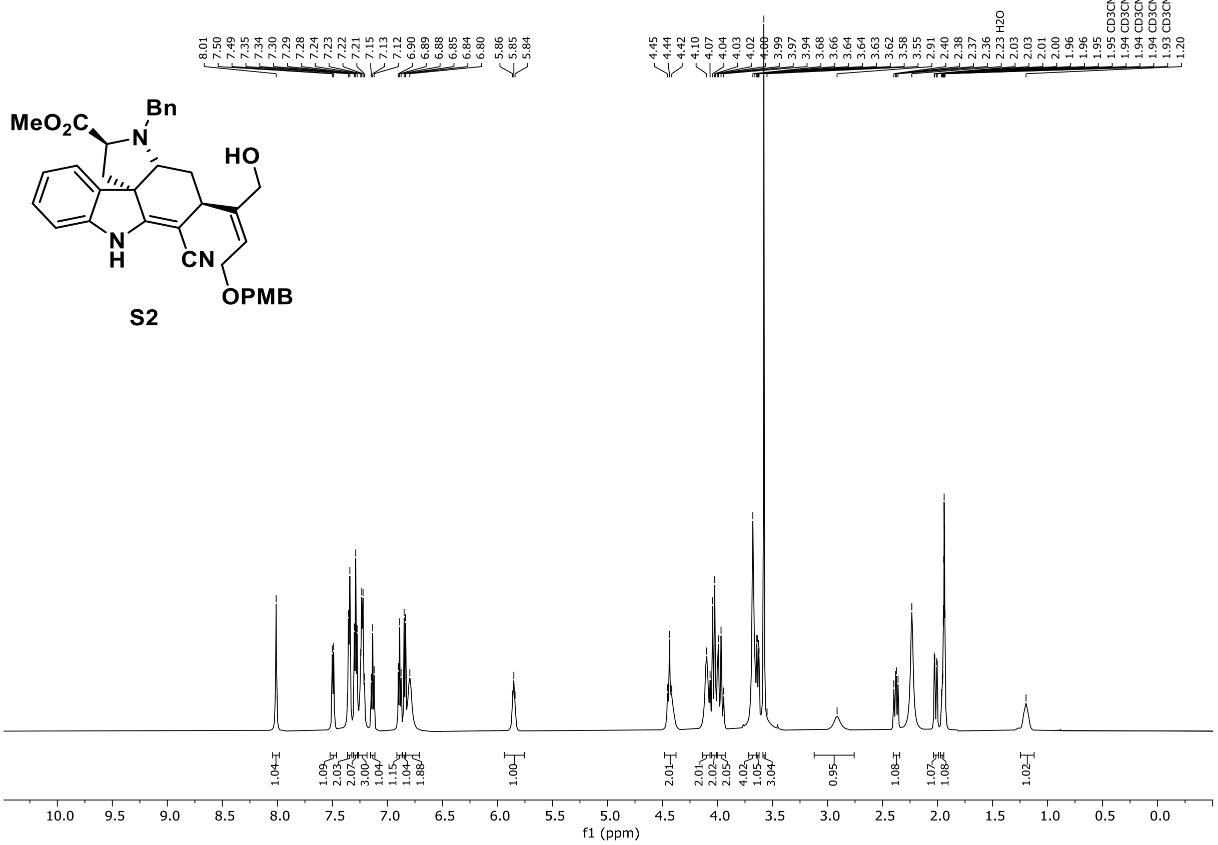

Figure S 32: ^{1}H NMR (CD_3CN, 600 MHz, 323 K) of compound **S2**.

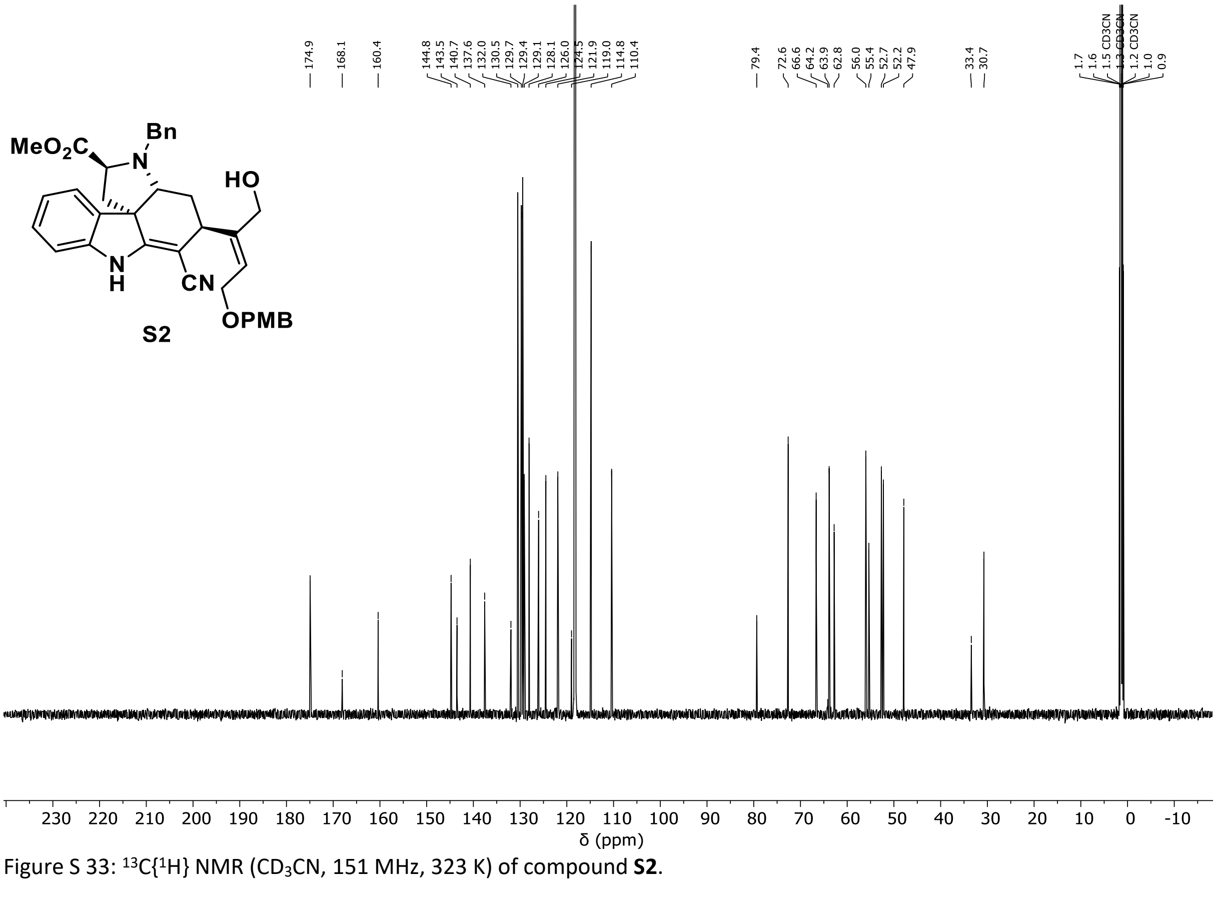

Figure S 33: $^{13}C\{^{1}H\}$ NMR (CD_3CN, 151 MHz, 323 K) of compound **S2**.

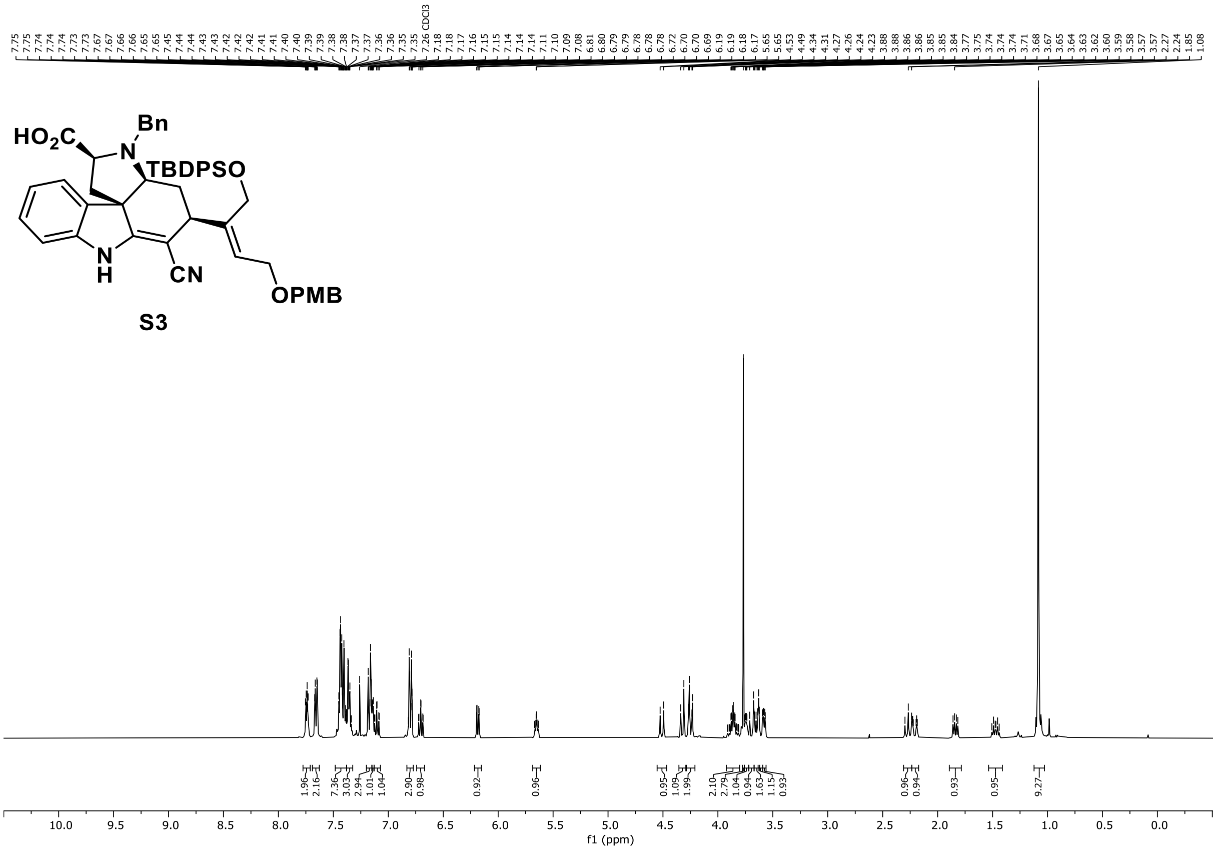

Figure S 34: ^{1}H NMR ($CDCl_3$, 400 MHz, 296 K) of compound **S3**.

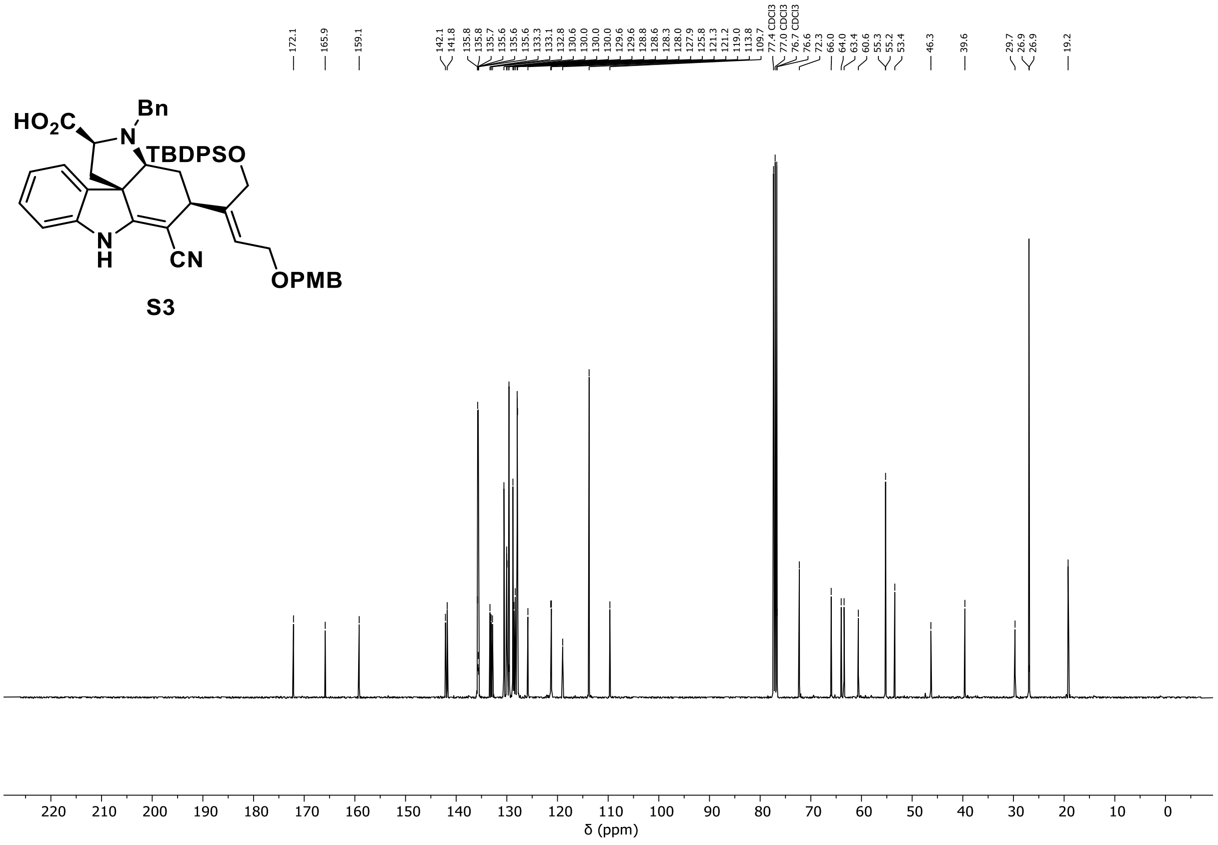

Figure S 35: $^{13}C\{^{1}H\}$ NMR ($CDCl_3$, 101 MHz, 296 K) of compound **S3**.

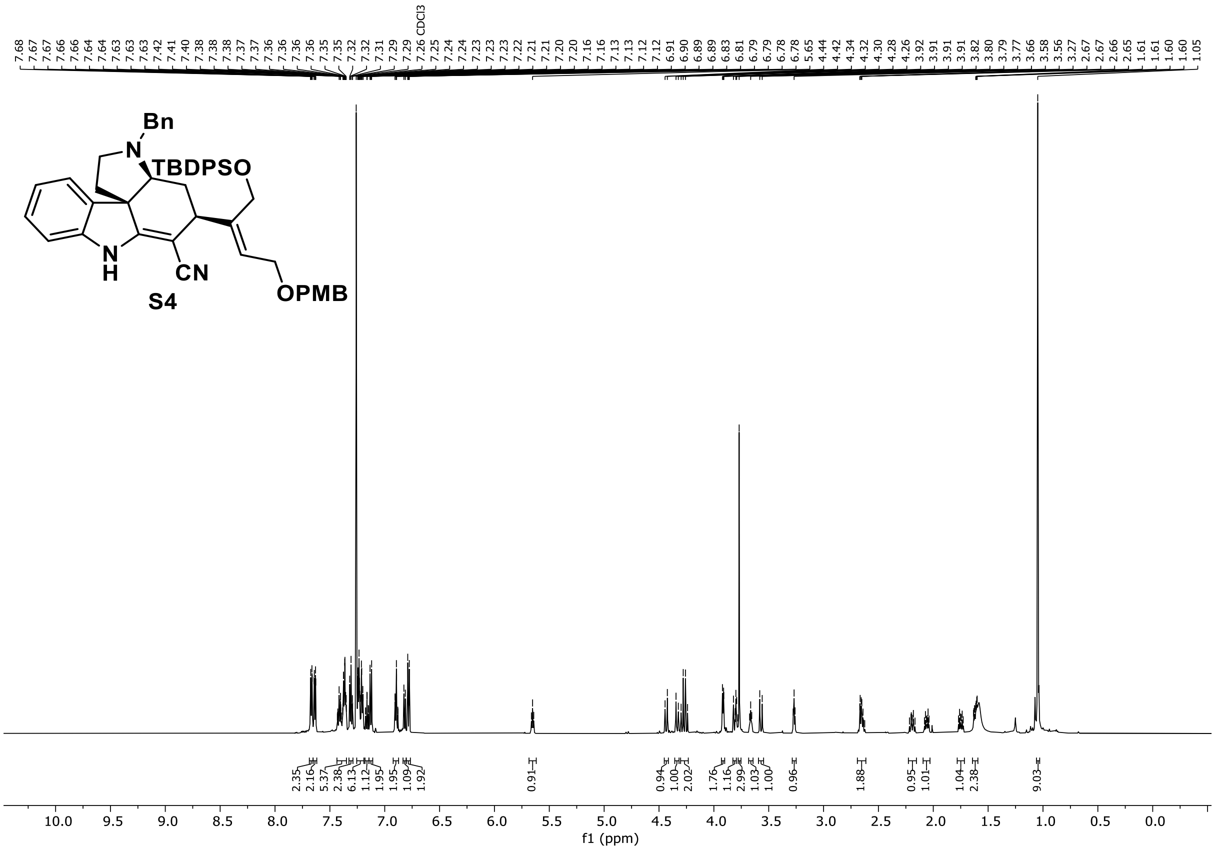

Figure S 36: ^{1}H NMR ($CDCl_3$, 600 MHz, 296 K) of compound **S4**.

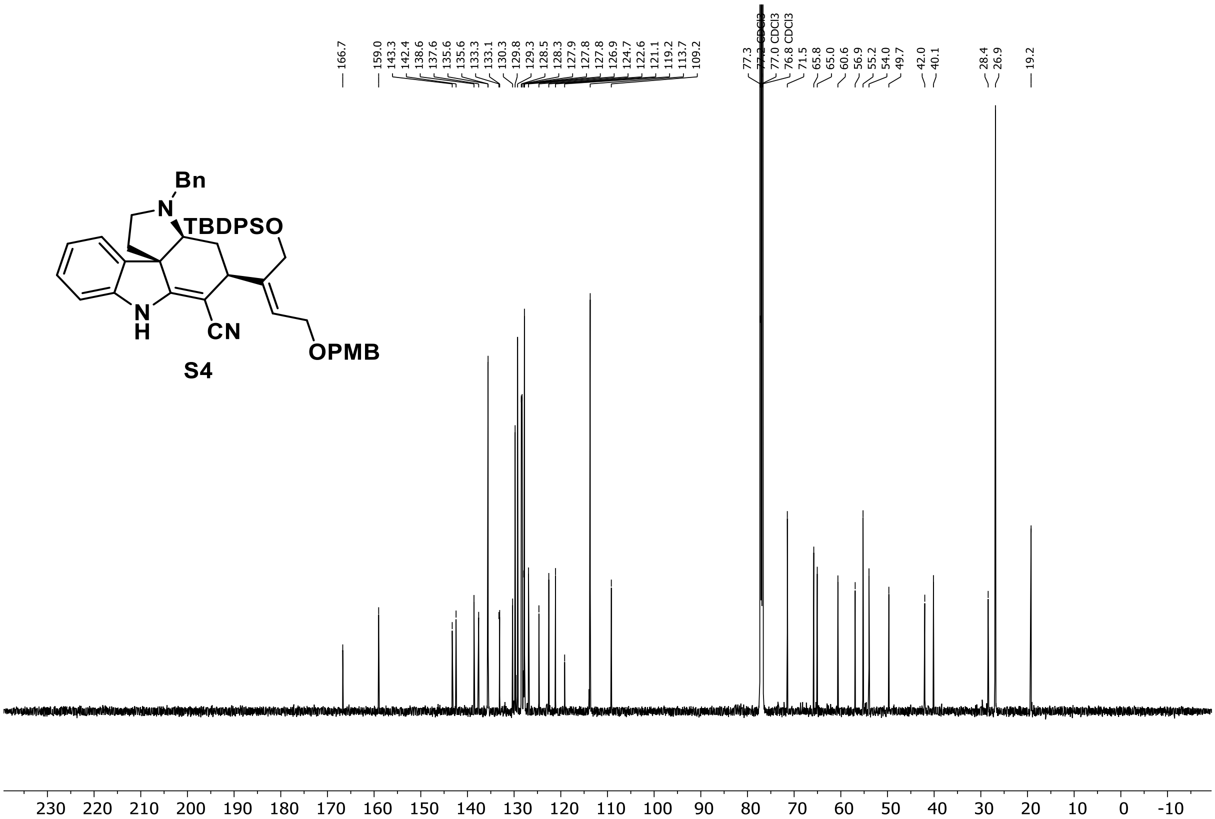

Figure S 37: $^{13}C\{^{1}H\}$ NMR ($CHCl_3$, 151 MHz, 296 K) of compound **S4**.

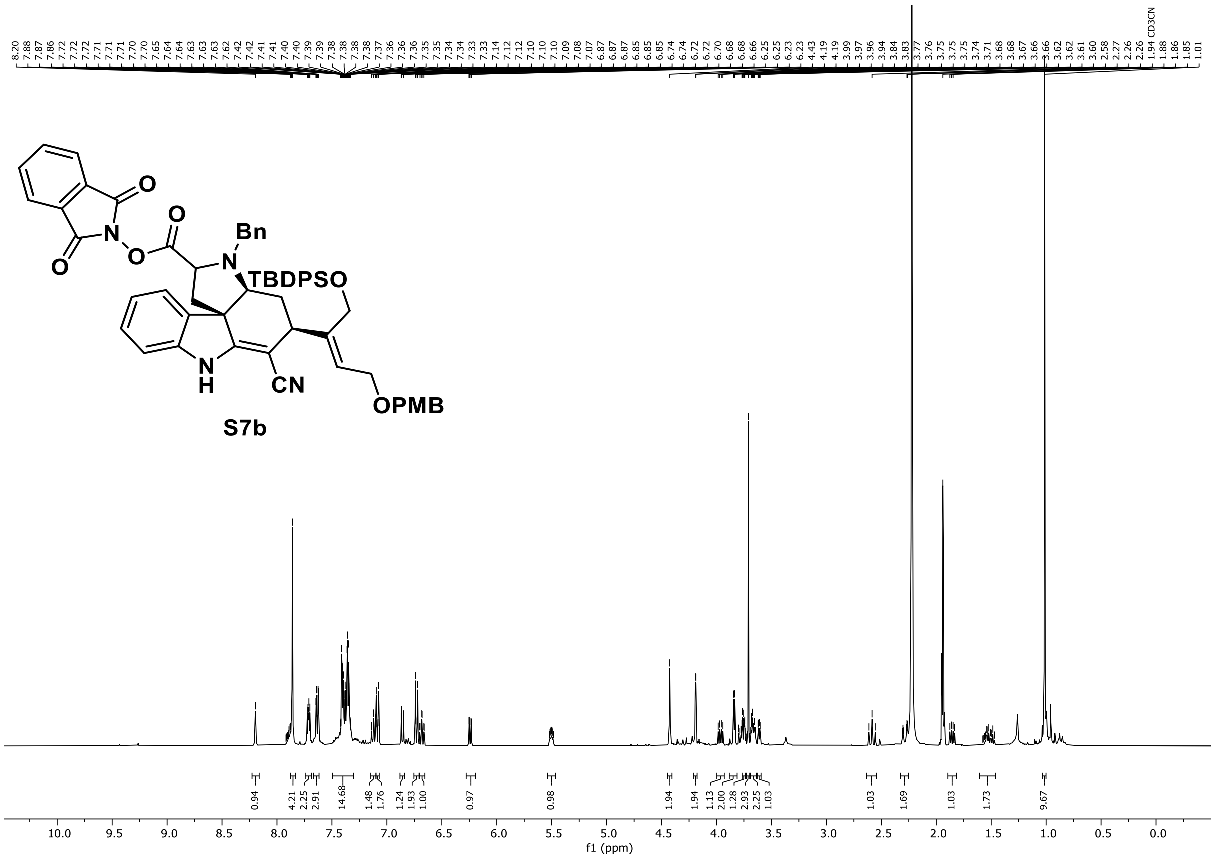

Figure S 38: ^{1}H NMR (CD_3CN, 400 MHz, 296 K) of compound **S7b**.

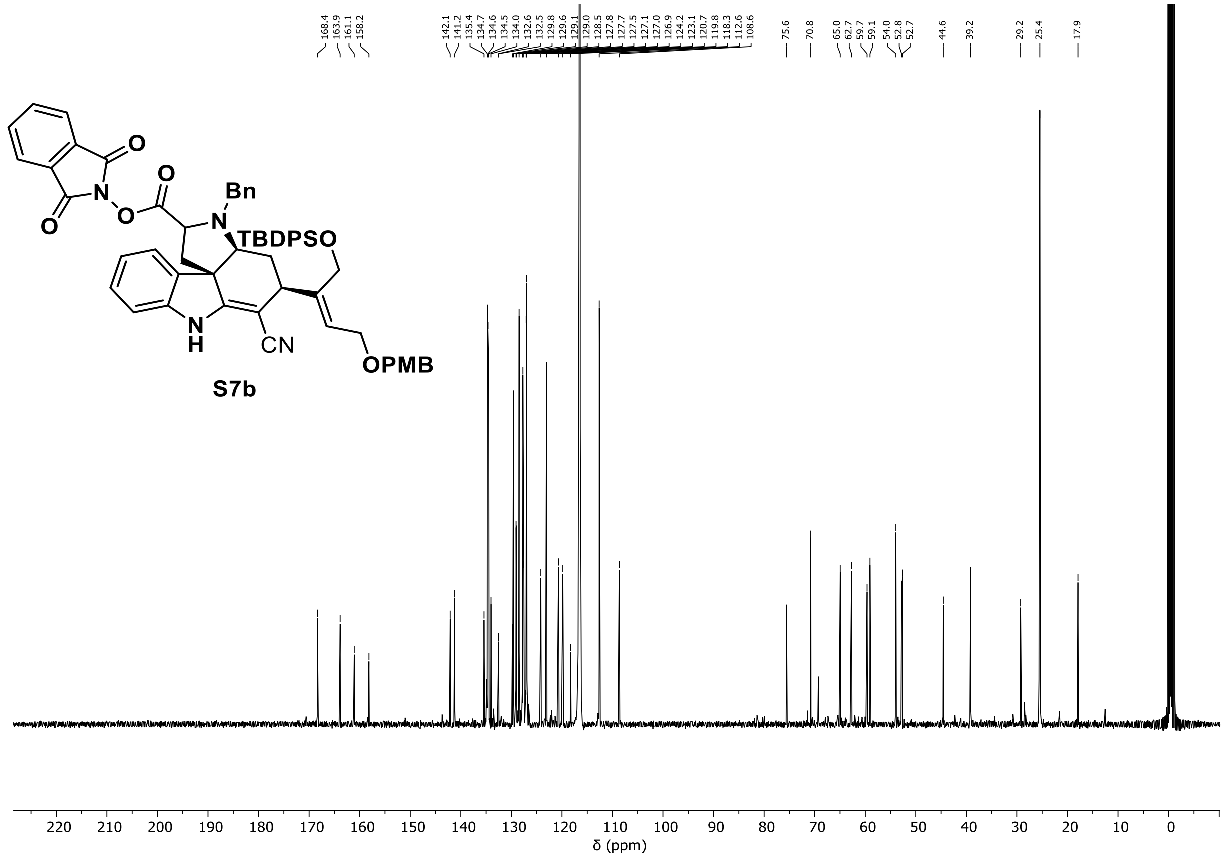

Figure S 39: $^{13}C\{^1H\}$ NMR (CD_3CN, 101 MHz, 296 K) of compound **S7b**.

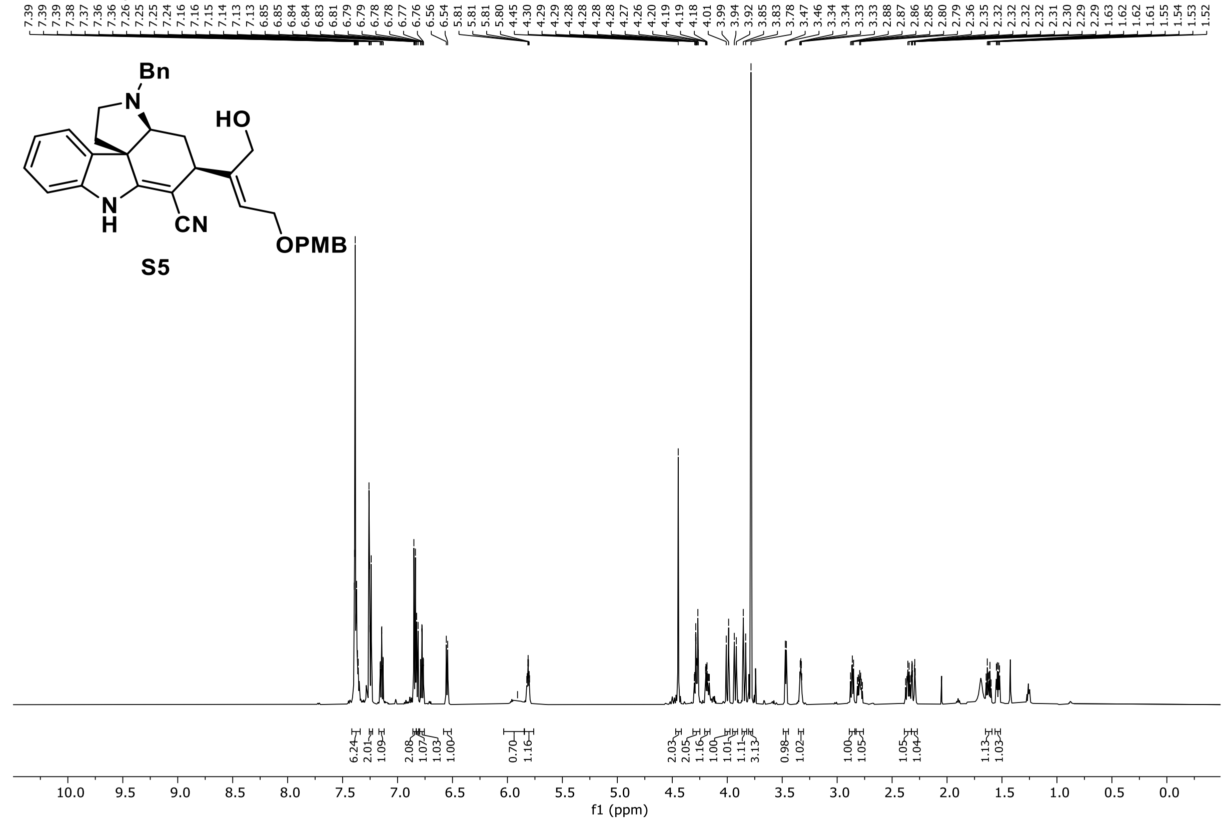

Figure S 40: ^1H NMR ($CDCl_3$, 600 MHz, 296 K) of compound **S5**.

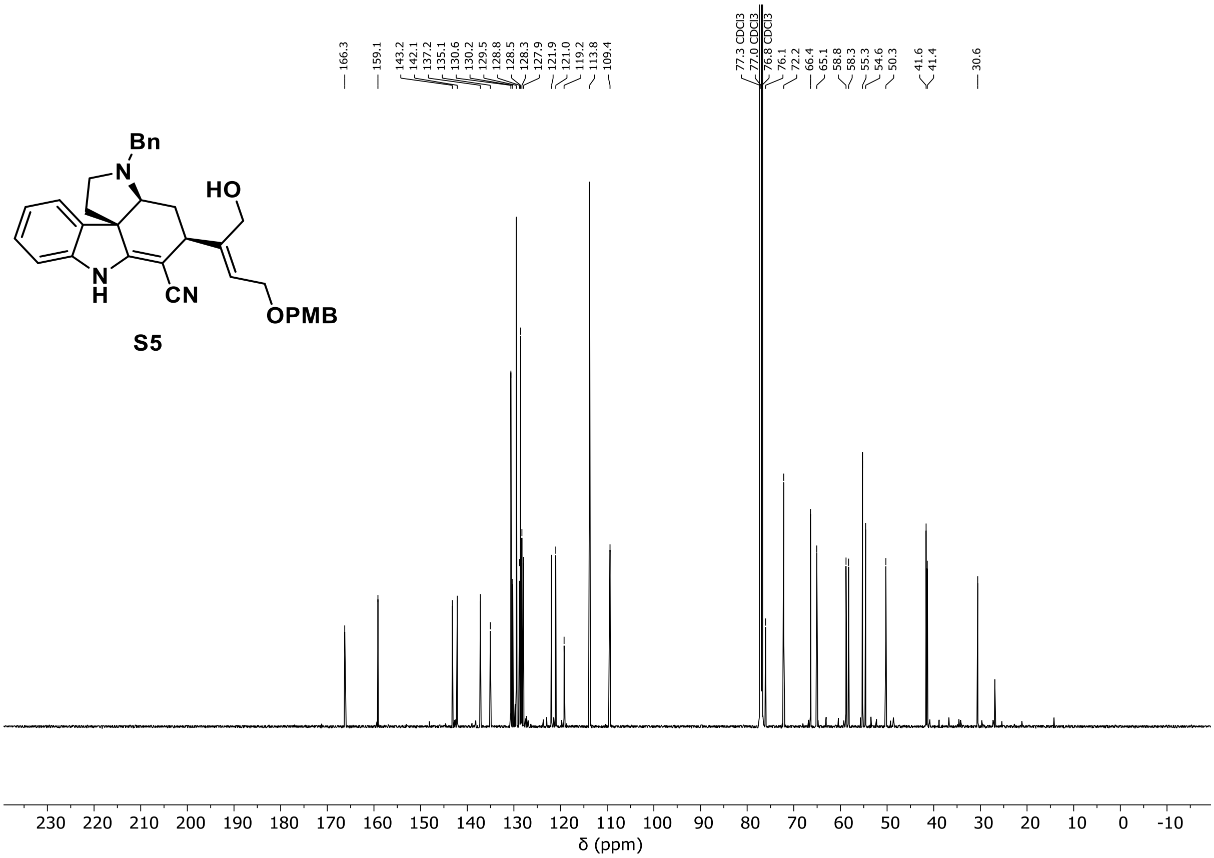

Figure S 41: $^{13}C\{^{1}H\}$ NMR ($CDCl_3$, 151 MHz, 296 K) of compound **S5**.

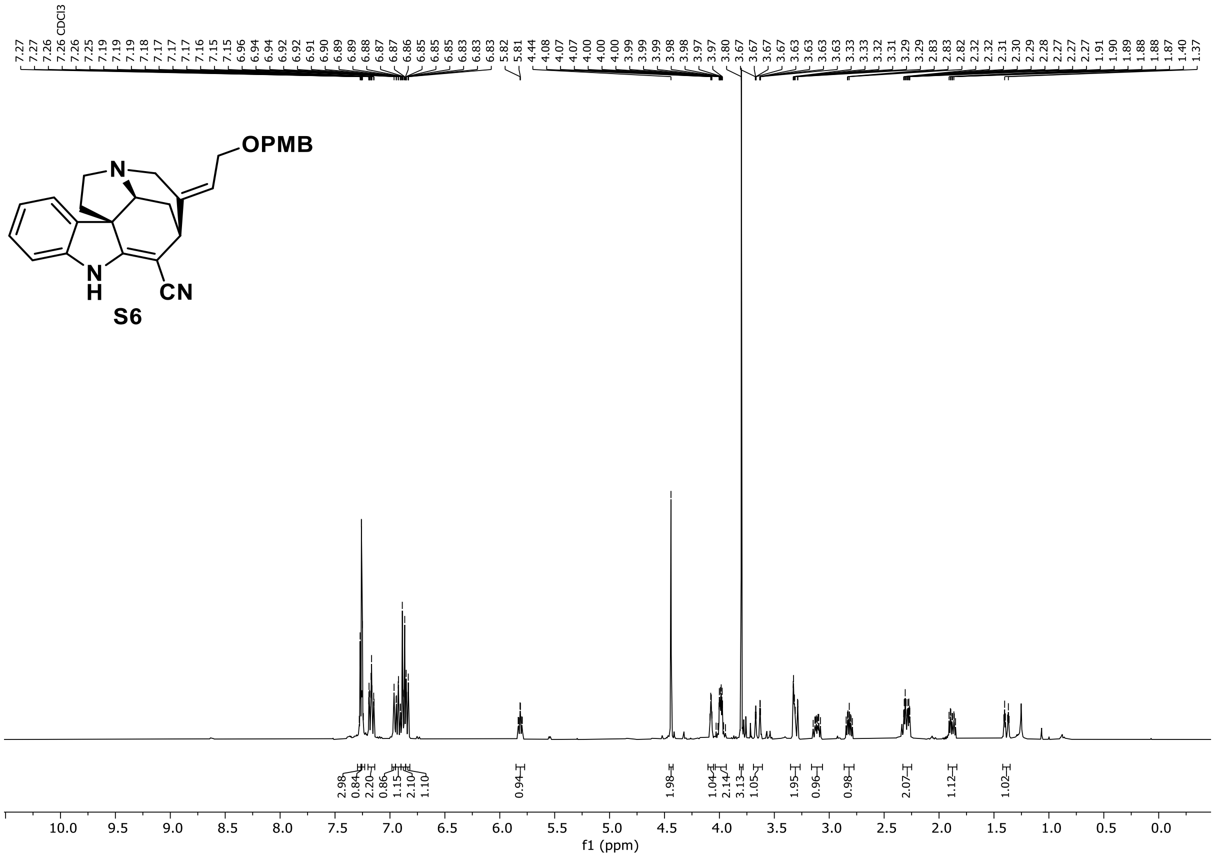

Figure S 42: ^{1}H NMR ($CDCl_3$, 400 MHz, 296 K) of compound **S6**.

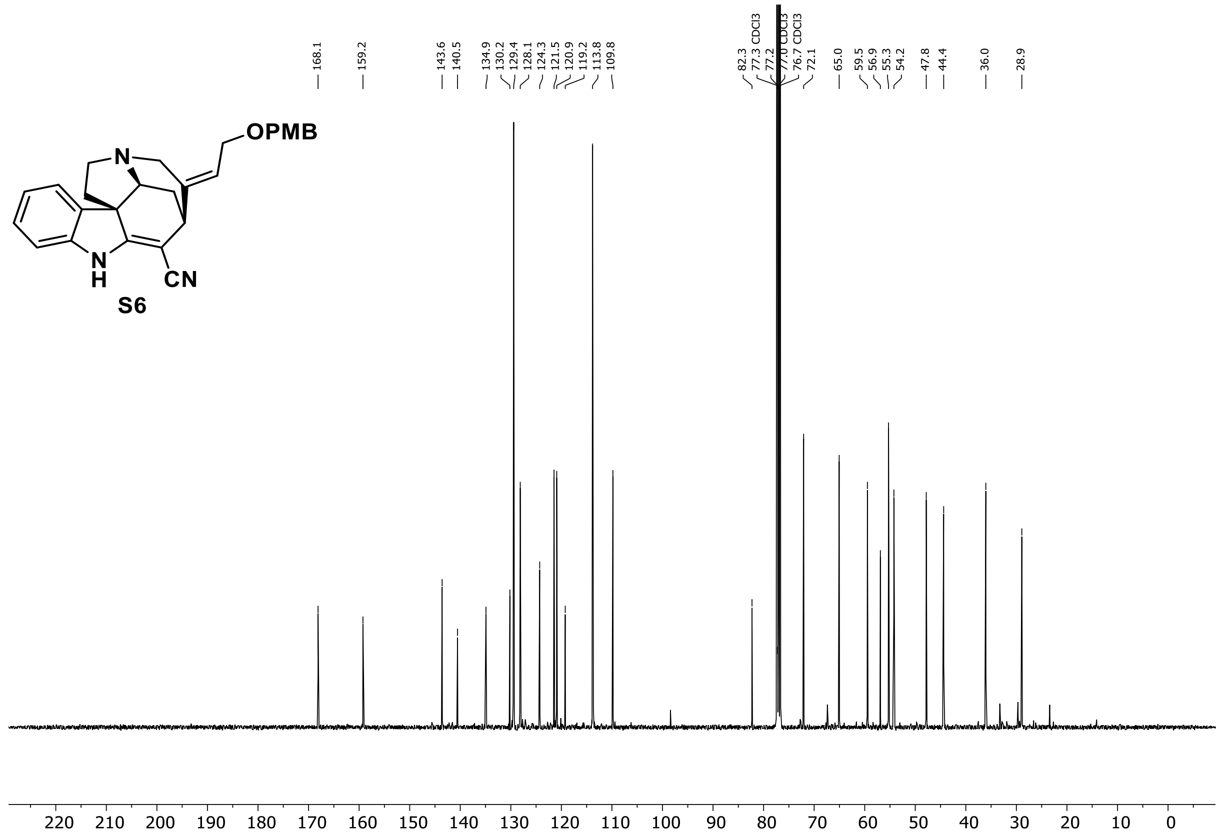

Figure S 43: $^{13}C\{^{1}H\}$ NMR ($CDCl_3$, 101 MHz, 296 K) of compound **S6**.

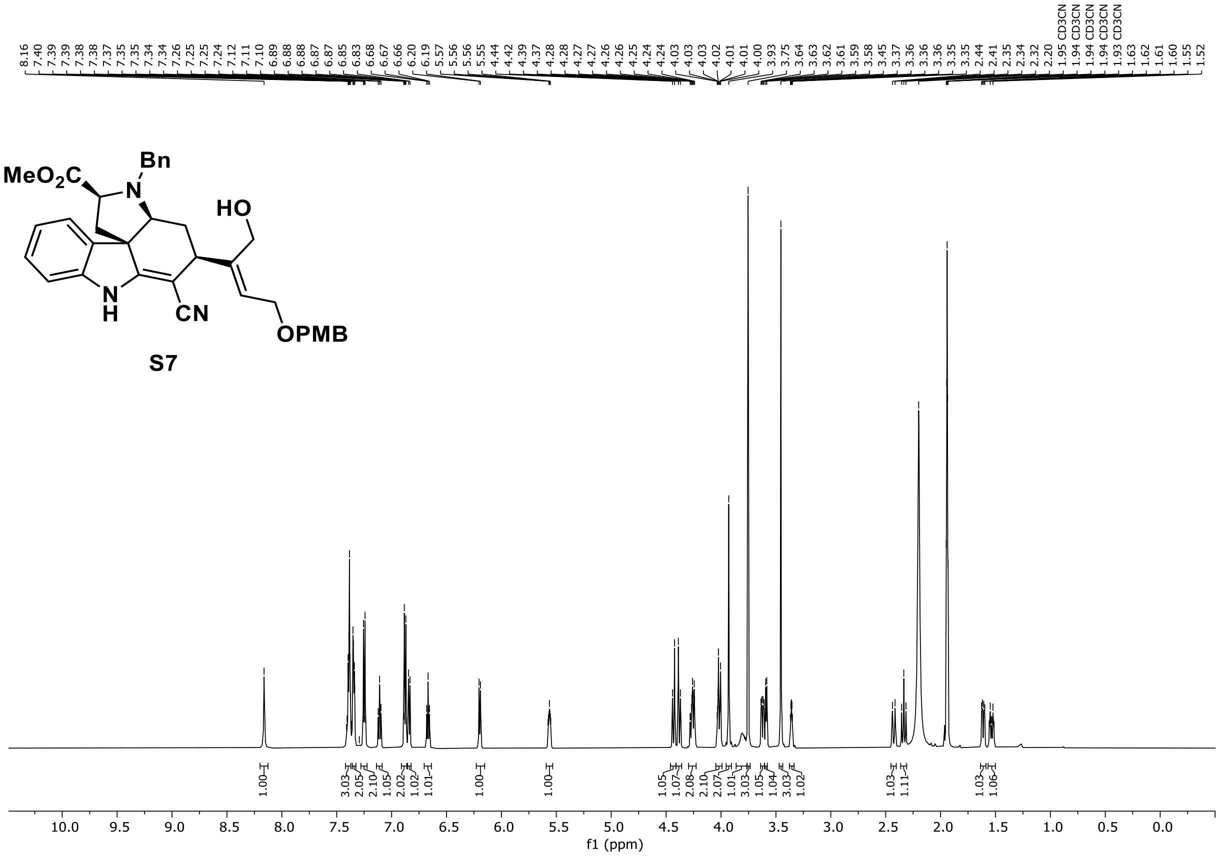

Figure S 44: ^{1}H NMR (CD_3CN, 600 MHz, 296 K) of compound **S7**.

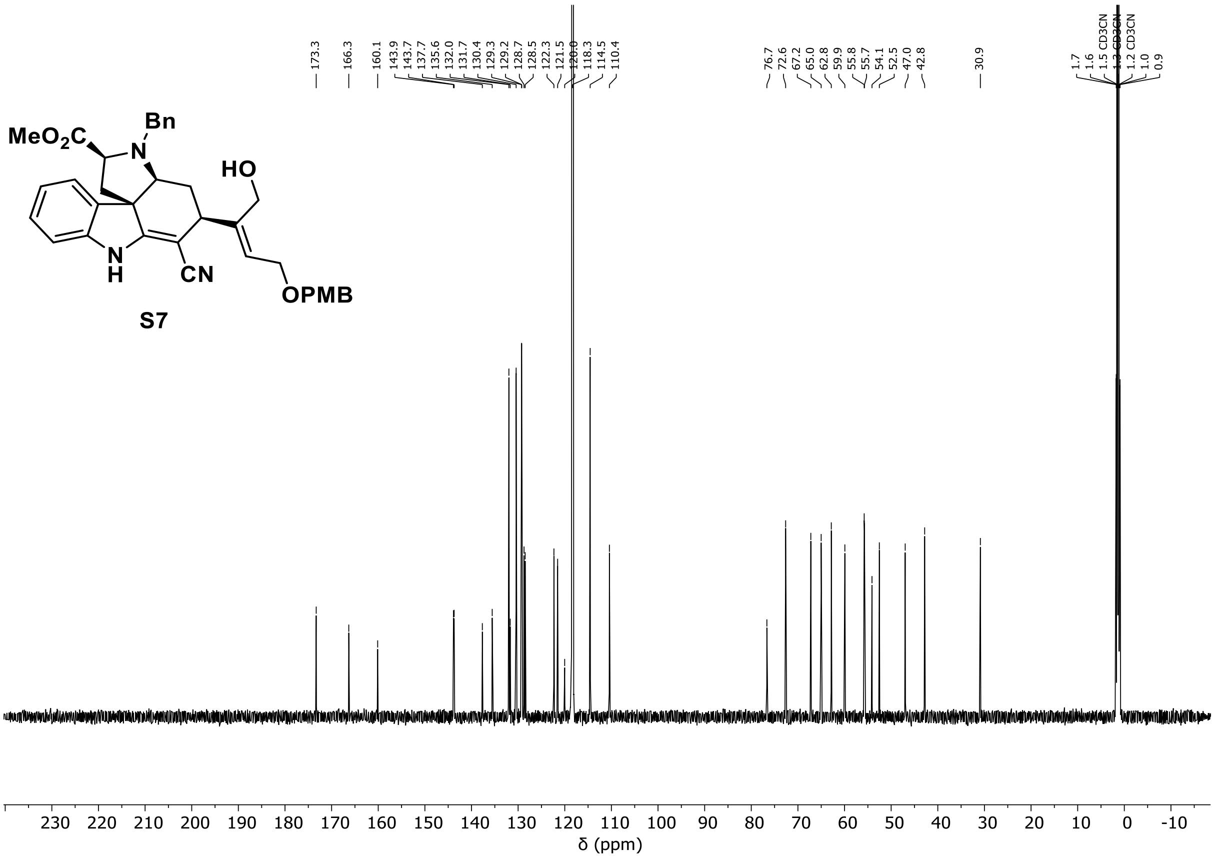

Figure S 45: $^{13}C\{^{1}H\}$ NMR (CD_3CN, 151 MHz, 296 K) of compound **S7**.

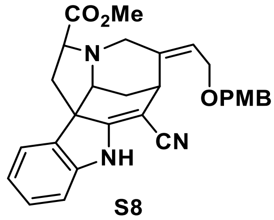

S8

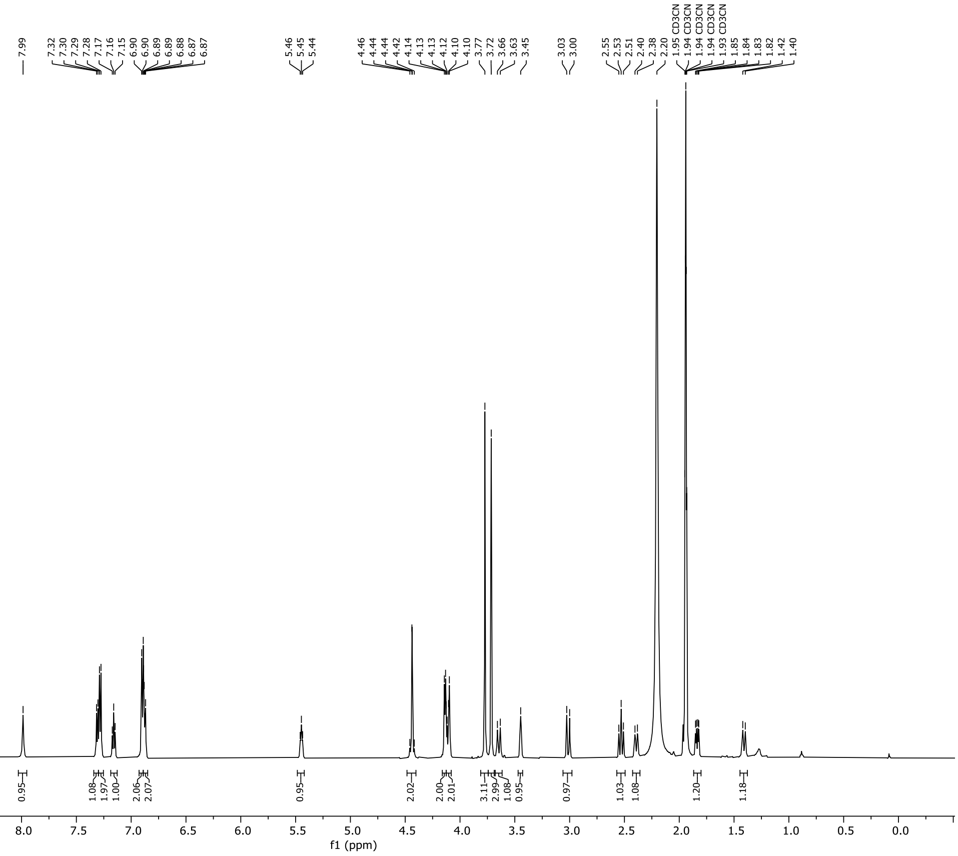

Figure S 46: ^{1}H NMR (CD_3CN, 600 MHz, 296 K) of compound **S8**.

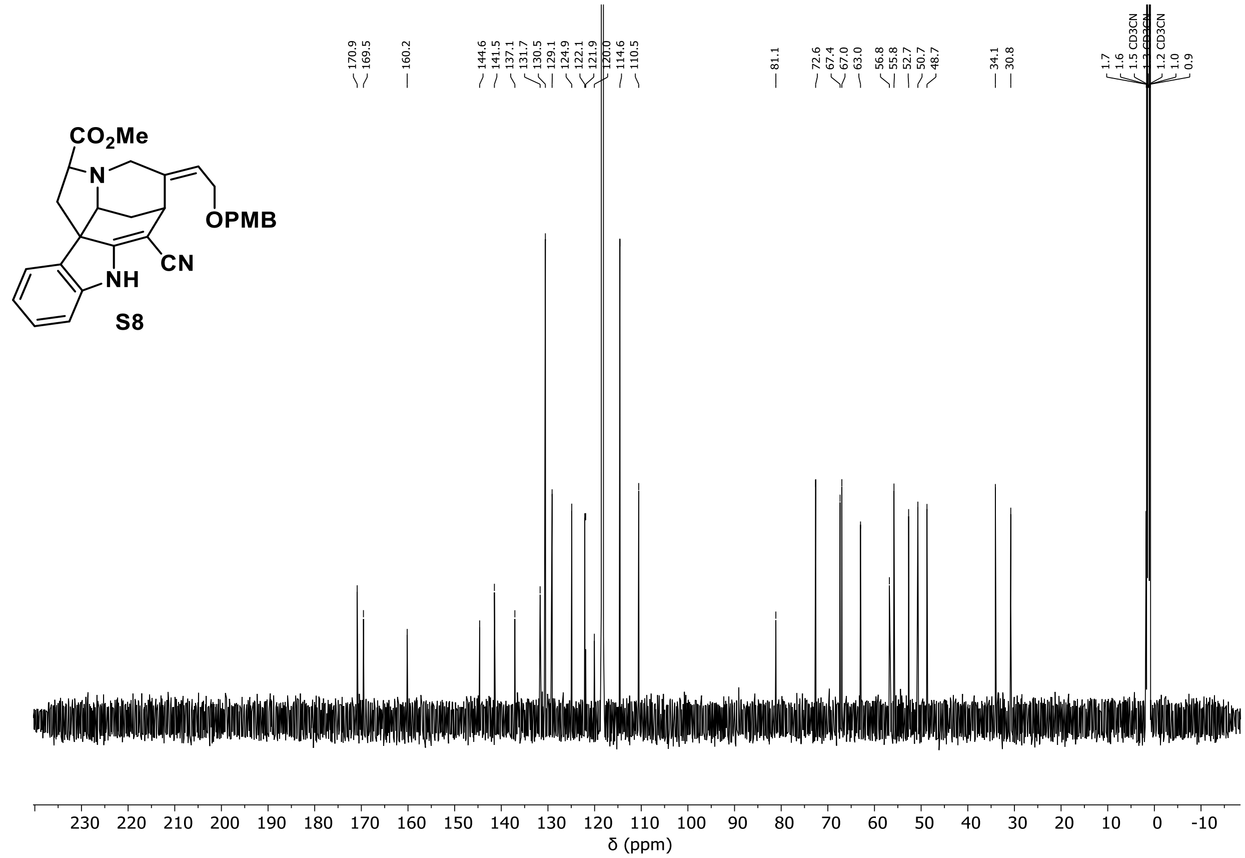

Figure S 47: $^{13}C\{^{1}H\}$ NMR (CD_3CN, 151 MHz, 296 K) of compound **S8**.

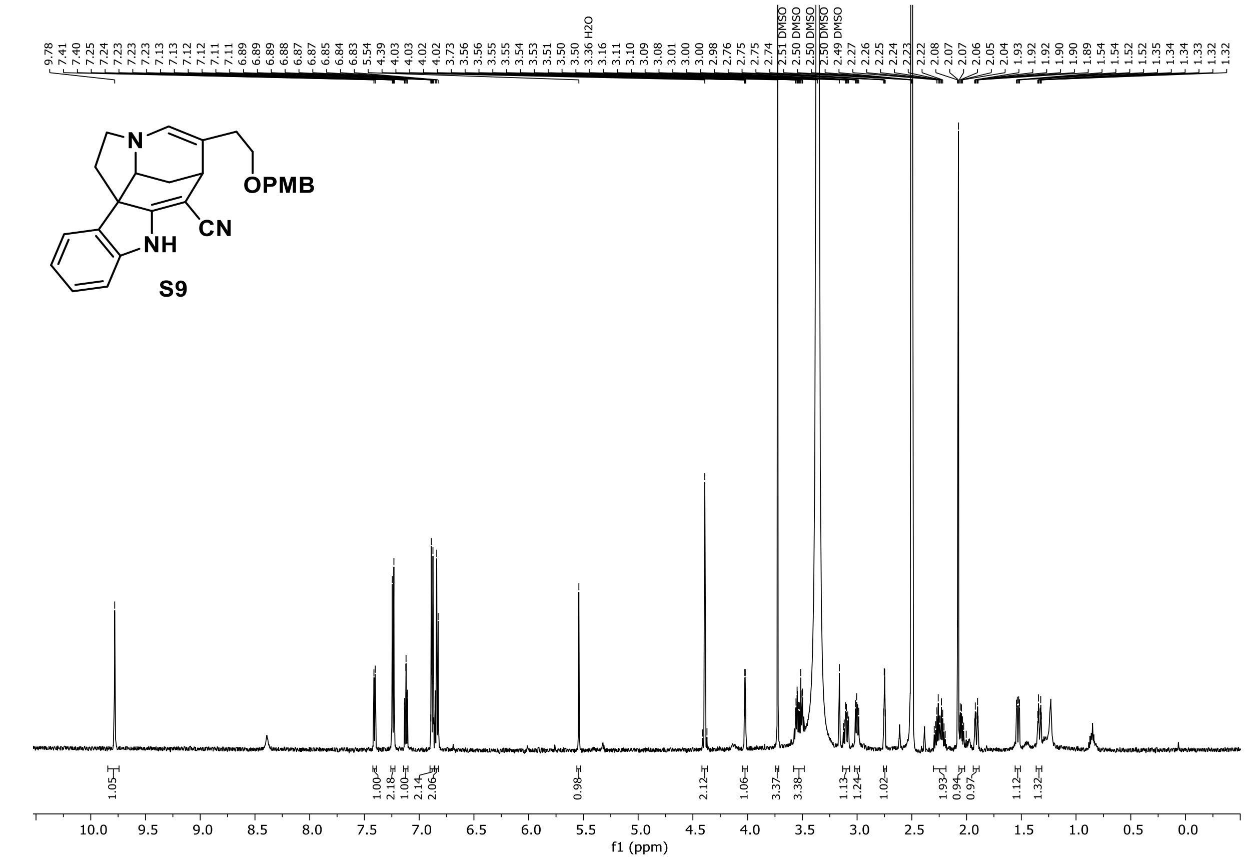

Figure S 48: ^{1}H NMR (CD_3CN, 600 MHz, 296 K) of compound **S9**.

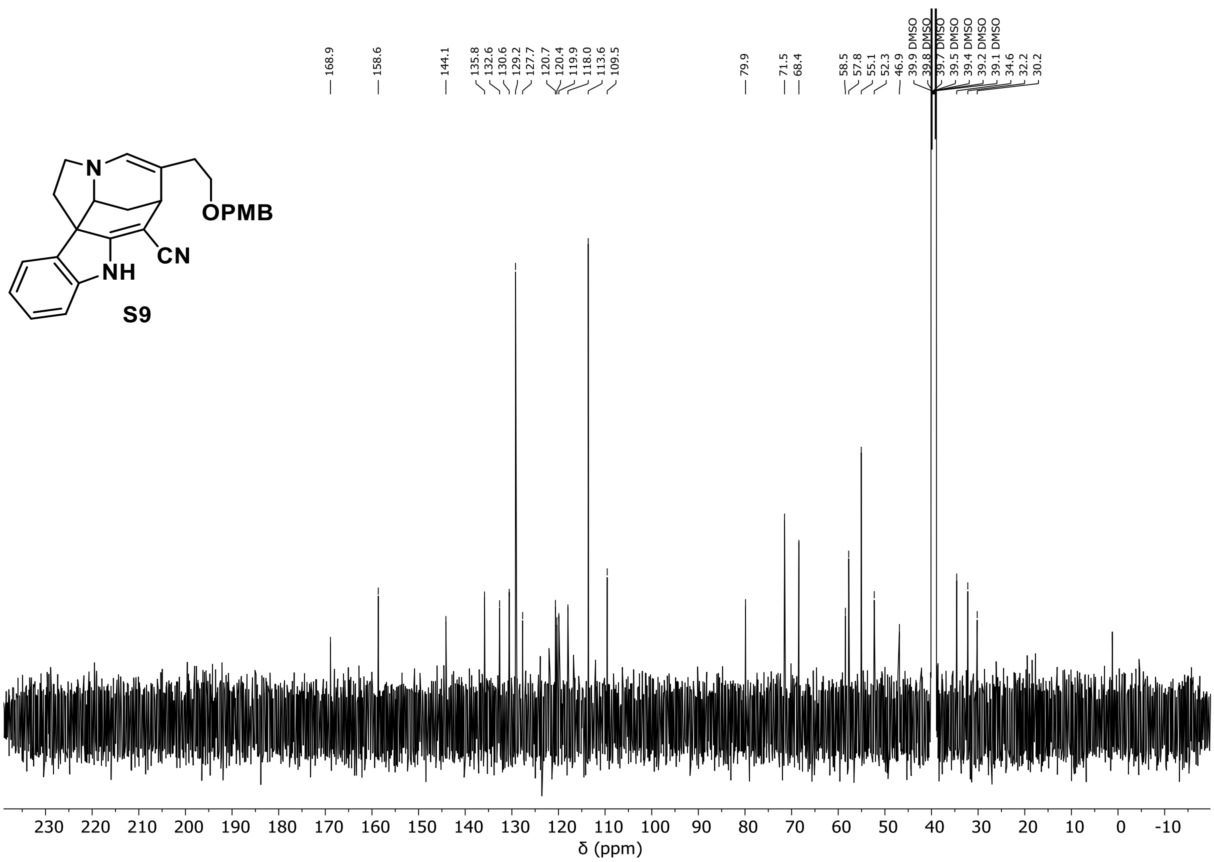

Figure S 49: $^{13}C\{^{1}H\}$ NMR (CD_3CN, 151 MHz, 296 K) of compound **S9**.

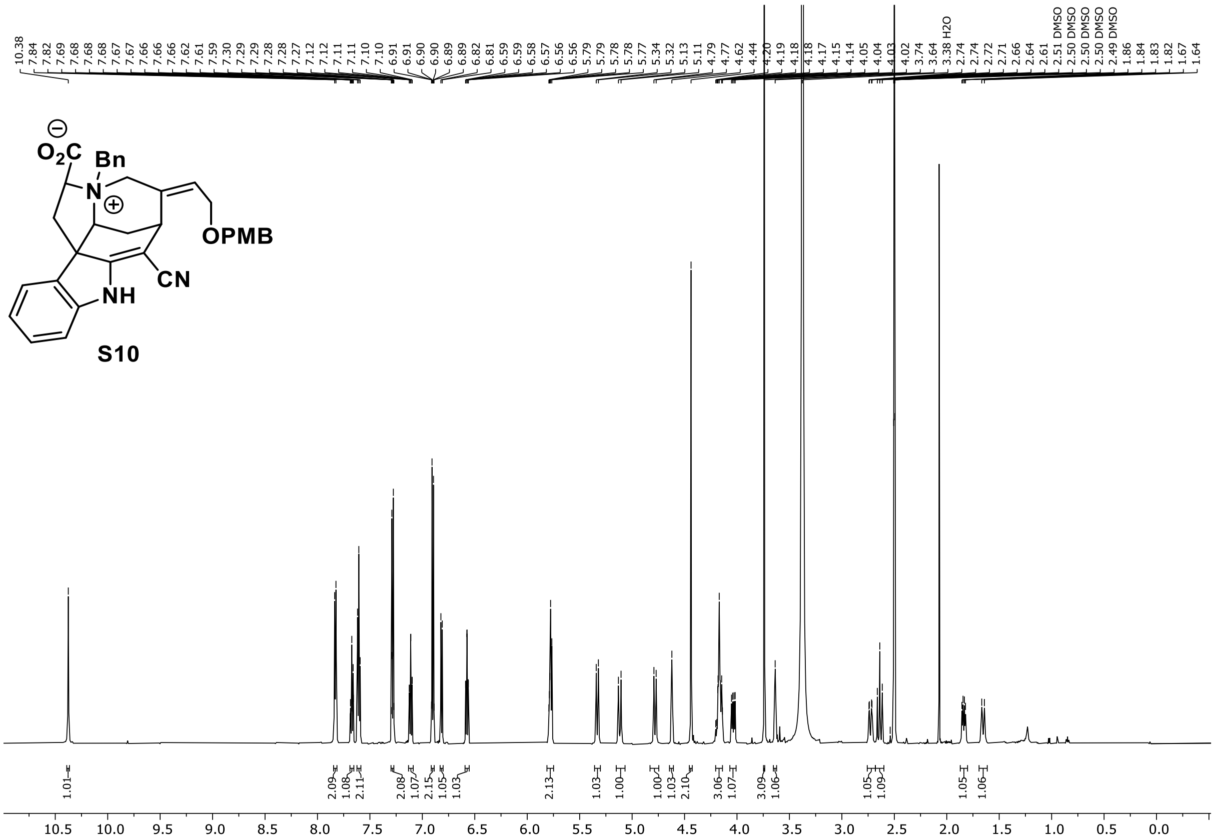

Figure S 50: ^{1}H NMR (DMSO-d_6, 600 MHz, 296 K) of compound **S10**.

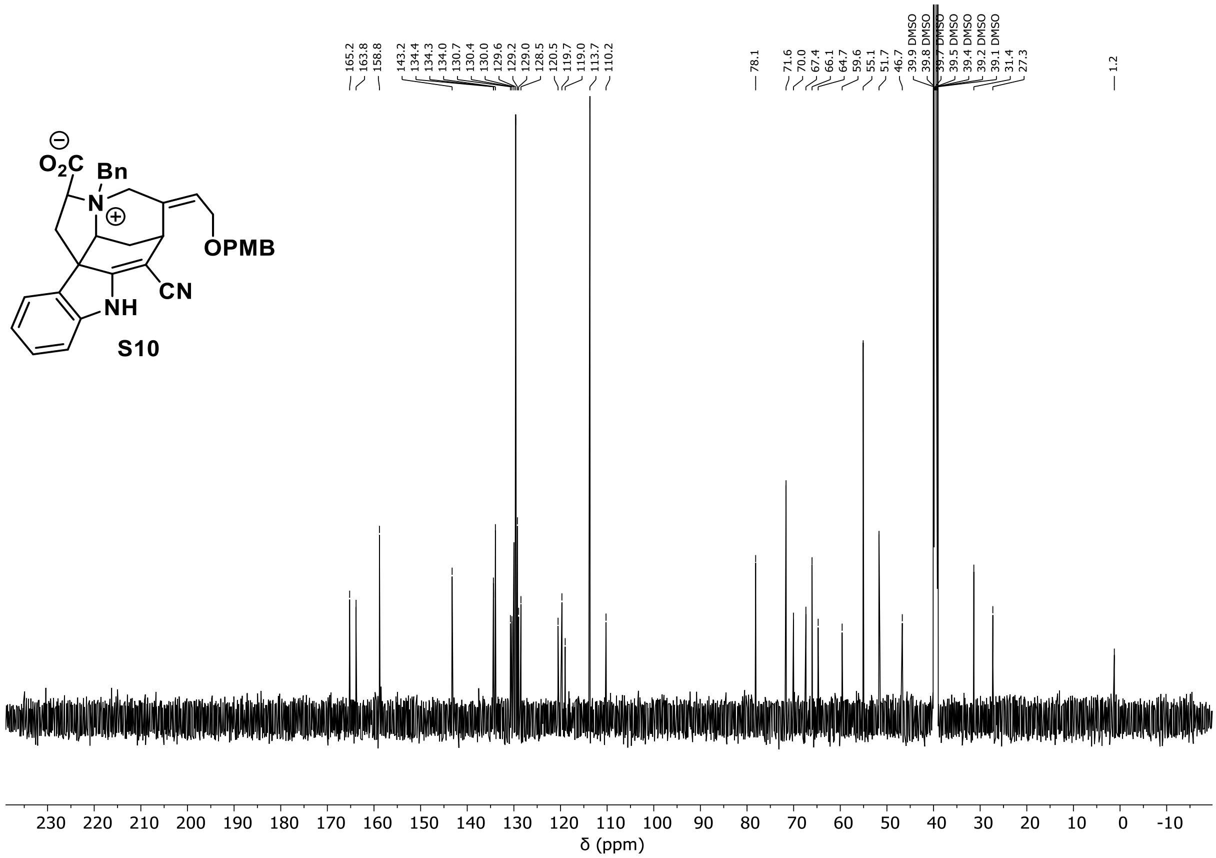

Figure S 51: $^{13}C\{^{1}H\}$ NMR (DMSO-d_6, 151 MHz, 296 K) of compound **S10**.

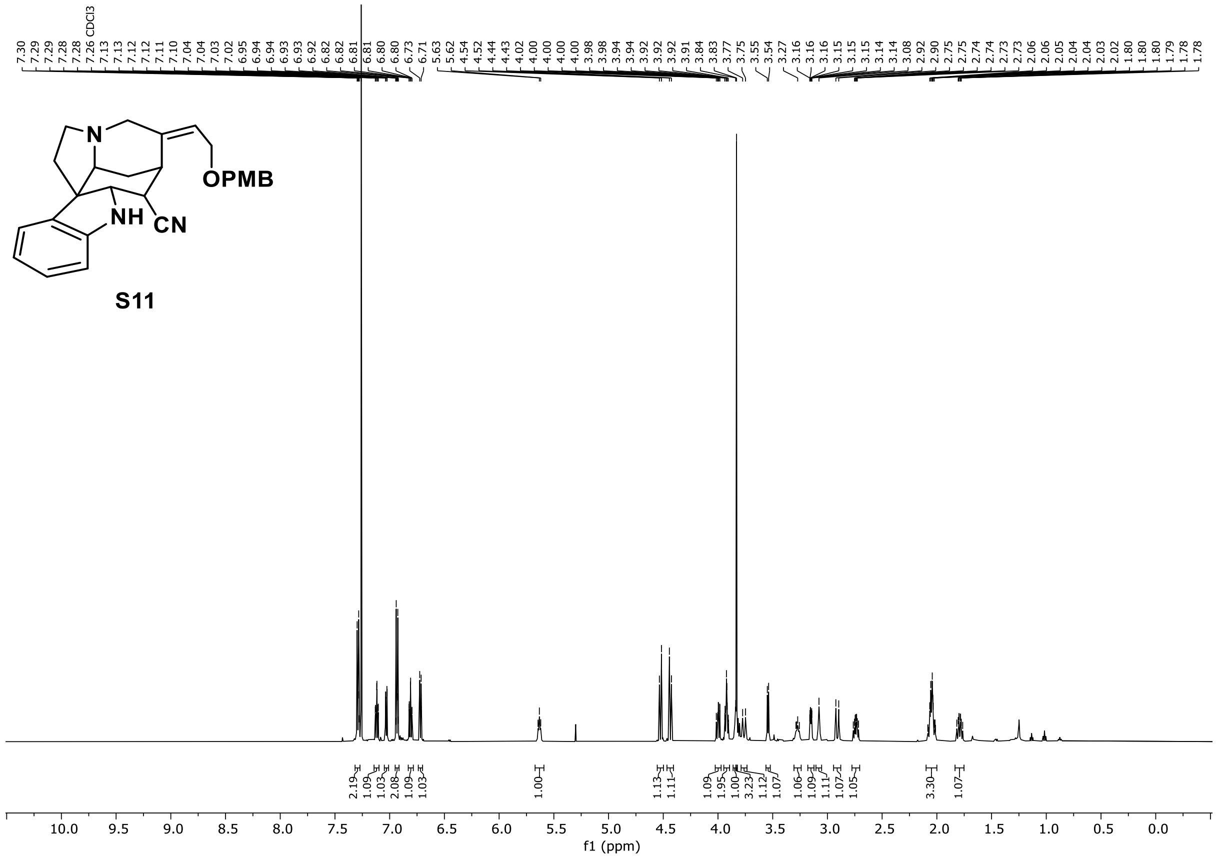

Figure S 52: ^{1}H NMR ($CDCl_3$, 600 MHz, 296 K) of compound **S11**.

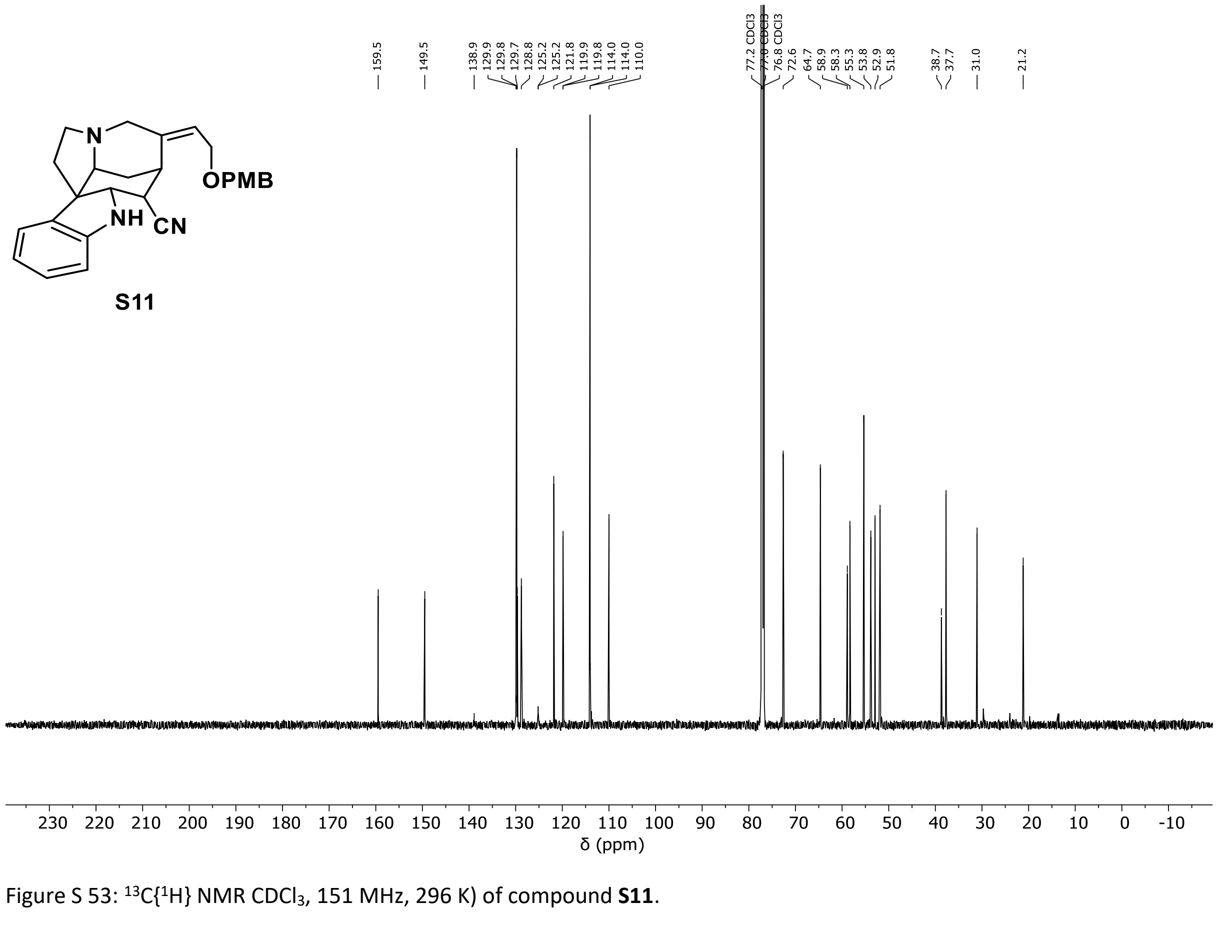

Figure S 53: $^{13}C\{^{1}H\}$ NMR $CDCl_3$, 151 MHz, 296 K) of compound **S11**.

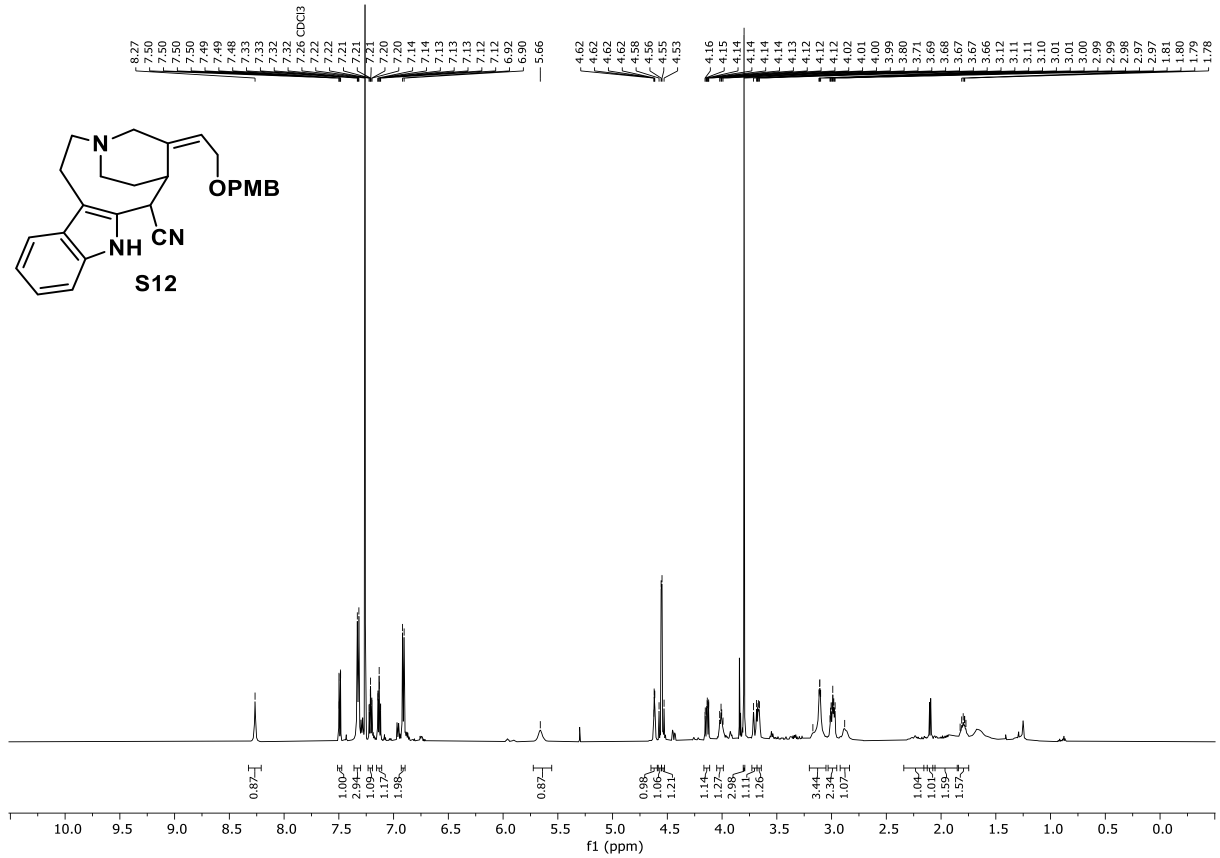

Figure S 54: ^1H NMR ($CDCl_3$, 600 MHz, 296 K) of compound **S12**.

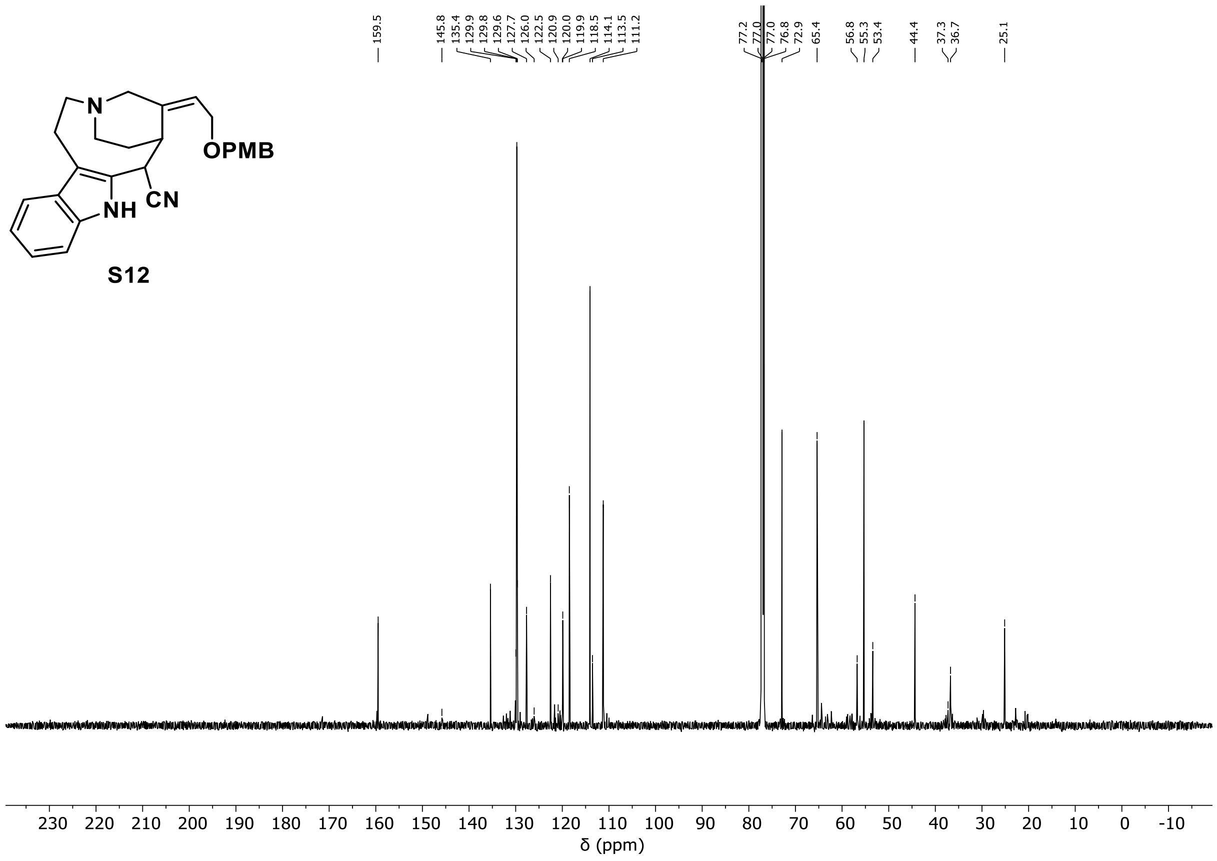

Figure S 55: $^{13}C\{^{1}H\}$ NMR $CDCl_3$, 151 MHz, 296 K) of compound **S12**.

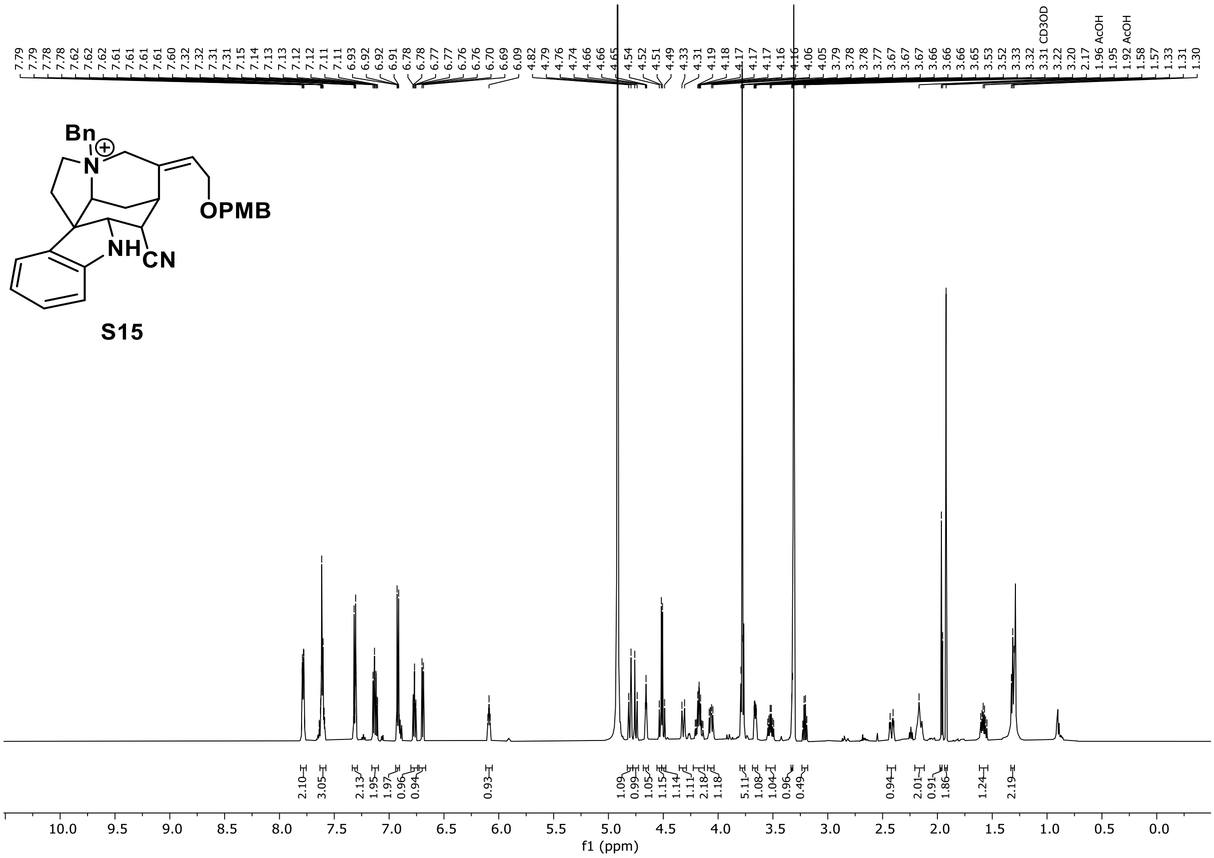

Figure S 56: ^{1}H NMR (CD_3OD, 600 MHz, 296 K) of compound **S15**.

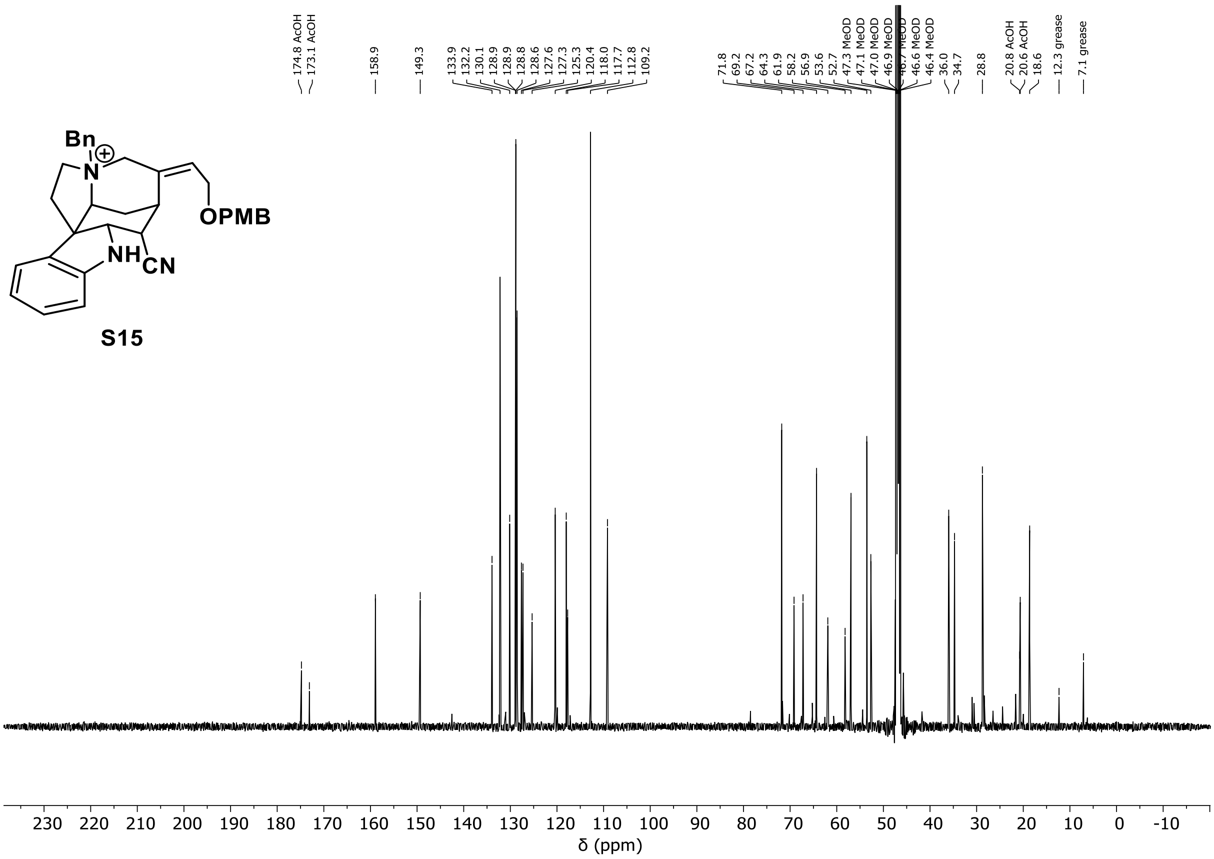

Figure S 57: $^{13}C\{^{1}H\}$ NMR [CD_3OD, 151 MHz, 296 K) of compound **S15**.

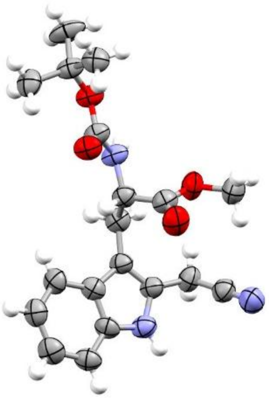

Table S 6: Crystal data and structure refinement for compound **10**

Identification code	**10**	
Empirical formula	$C_{19}H_{23}N_3O_4$	
moiety formula	$C_{19}H_{23}N_3O_4$	
Formula weight	357.40	
Temperature	120(2) K	
Wavelength, radiation type	1.54178Å, CuK_α	
Diffractometer	STOE IPDS 2T	
Crystal system	Monoclinic	
Space group name, number	C 2, (5)	
Unit cell dimensions	a = 25.771(3) Å	α = 90°
	b = 5.1629(4) Å	β = 95.468(9)°
	c = 14.4494(16) Å	γ = 90°
Volume	1913.8(3) $Å^3$	
Number of reflections	13828	
and range used for lattice parameters	3.07° <=θ<= 67.96°	
Z	4	
Density (calculated)	1.240 Mg/m^3	

Absorption coefficient	0.723 mm^{-1}
Absorption correction	Integration
Max. and min. transmission	0.9898 and 0.8118
F(000)	760
Crystal size, colour and form needle	0.010 x 0.020 x 0.580 mm^3, colourless
Theta range for data collection	3.072 to 67.929°.
Index ranges	-27<=h<=30, -5<=k<=5, -16<=l<=17
Number of reflections:	
collected	10797
independent	3347 [R(int) = 0.2324]
observed [I>2sigma(I)]	2283
Completeness to theta = 67.7°	98.0 %
Refinement method	Full-matrix least-squares on F^2
Data / restraints / parameters	3347 / 1 / 238
Goodness-of-fit on F^2	1.085
Final R indices [I>2sigma(I)]	R1 = 0.1460, wR2 = 0.3403
R indices (all data)	R1 = 0.1816, wR2 = 0.4066
Absolute structure parameter	-1.4(10)
Largest diff. peak and hole	0.676 and -0.630 eÅ^{-3}

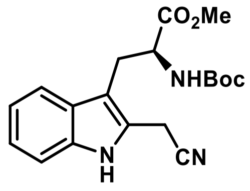

10

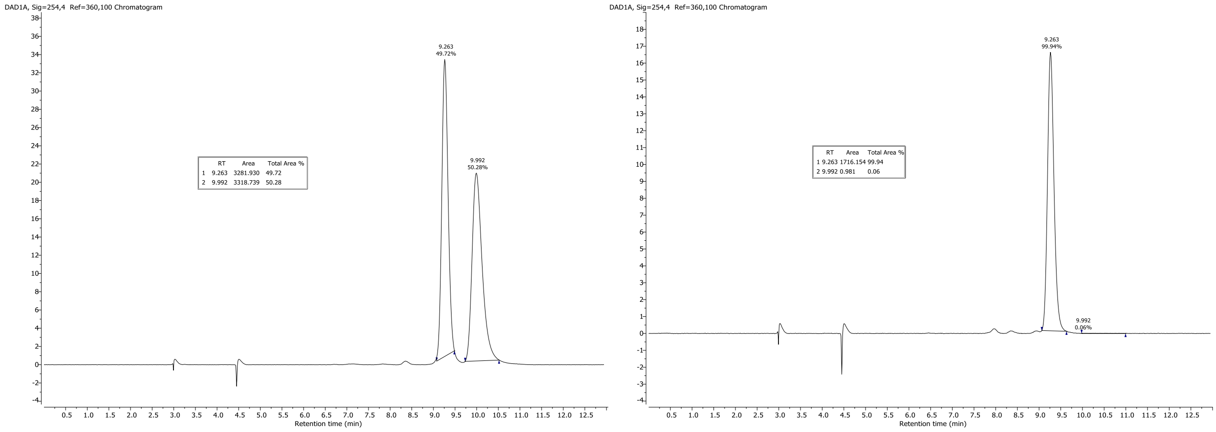

Figure S 58: HPLC chromatogram of compound **10**. (Chiralpak IF-3, *n*Hex/EtOH 90:10, 1 mL/min, 40 °C at 254 nm.

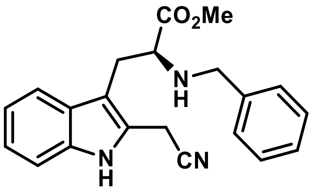

4

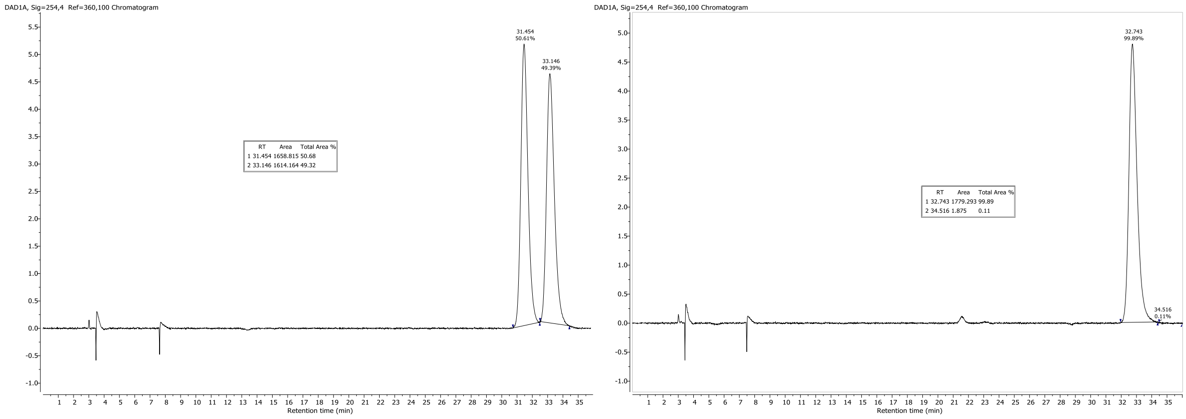

Figure S 59: HPLC chromatogram of compound **4**. (Chiralpak IF-3, *n*Hex/EtOH 97:3, 1 mL/min, 40 °C at 254 nm.

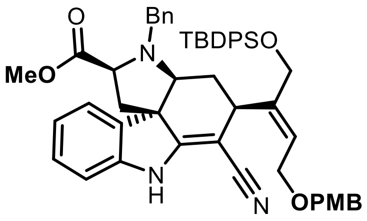

(*E*)-14

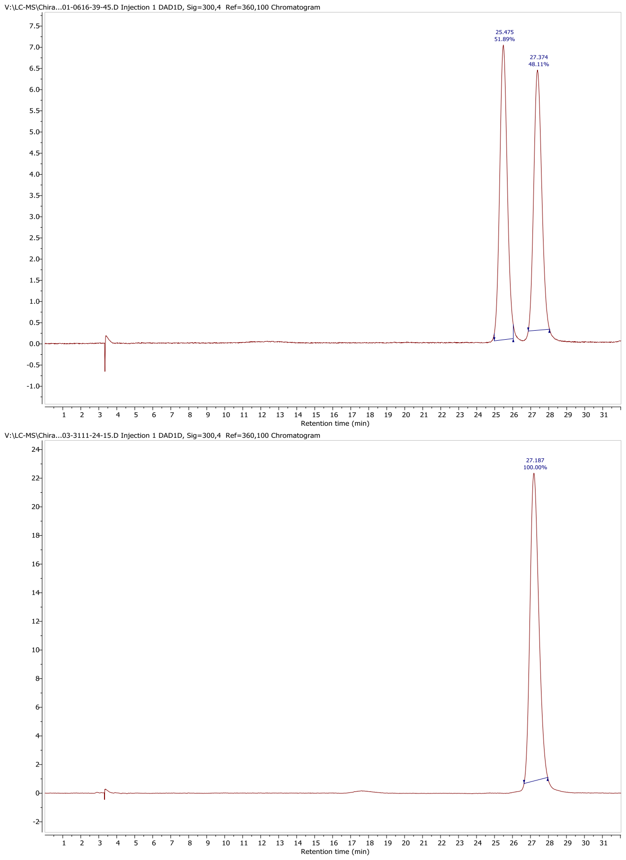

Figure S 60: HPLC chromatogram of compound **(*E*)-14**. (Chiralpak IF-3, *n*Hex/EtOH 97:3, 1 mL/min, 40 °C at 300 nm.
